# Proteasome-guided haem signalling axis contributes to T cell exhaustion

**DOI:** 10.1038/s41586-026-10250-y

**Published:** 2026-03-18

**Authors:** Yingxi Xu, Yangtao Shangguan, Yu-Ming Chuang, Tzu-Hsuan Chang, Wenbing Liu, Jhan-Jie Peng, Josep Garnica, Leling Xie, Pei-Chun Hsueh, Mei-Chun Lin, Yi-Hao Wang, Karina Lobo Hajdu, Yibo Wu, Maryam Akrami, Chen Wang, Anna Kohl, Alfred Zippelius, Wei Qi, Min Wang, Bugi Ratno Budiarto, Shih-Yu Chen, Zhengtao Xiao, Panagiota Vardaka, Rahul Roychoudhuri, Zhiliang Bai, Rong Fan, Santiago Carmona, Yi-Ru Yu, Christoph Scheiermann, Jianxiang Wang, Ping-Chih Ho

**Affiliations:** 1 Department of Oncology, University of Lausanne, Lausanne, Switzerland; 2 Ludwig Institute for Cancer Research Lausanne branch, Lausanne, Switzerland; 3 State Key Laboratory of Experimental Hematology, National Clinical Research Center for Blood Diseases, Institute of Hematology and Blood Diseases Hospital, Chinese Academy of Medical Sciences and Peking Union Medical College, Tianjin, China; 4 Tianjin Institutes of Health Science, Tianjin, China; 5 Graduate Institute of Clinical Medical Sciences, Chang Gung University, Taoyuan, Taiwan; 6 Center for Molecular and Clinical Immunology, Chang Gung University, Taoyuan, Taiwan; 7 Swiss Institute of Bioinformatics, Lausanne, Switzerland; 8 Department of Otolaryngology, National Taiwan University Hospital, Taipei, Taiwan; 9 Chemical Biology Mass Spectrometry Platform (CHEMBIOMS), Faculty of Sciences, University of Geneva, Geneva, Switzerland; 10 Department of Biomedical Sciences, University of Lausanne, Lausanne, Switzerland; 11 Laboratory of Cancer Immunology, Department of Biomedicine, University of Basel and University Hospital of Basel, Basel, Switzerland; 12 Department of Pathology and Immunology, Faculty of Medicine, University of Geneva, Geneva, Switzerland; 13 Geneva Centre for Inflammation Research, Geneva, Switzerland; 14 National Key Laboratory of Immunity and Inflammation, Suzhou Institute of Systems Medicine, Chinese Academy of Medical Sciences & Peking Union Medical College, Suzhou, China; 15 Medical Oncology, University Hospital Basel, Basel, Switzerland; 16 Novogene Co. Ltd, Beijing, China; 17 Institute of Biomedical Sciences, Academia Sinica, Taipei, Taiwan; 18 Taiwan International Graduate Program in Molecular Medicine, National Yang Ming Chiao Tung University and Academia Sinica, Taipei, Taiwan; 19 School of Basic Medical Sciences, Xi’an Jiaotong University, Xi’an, China; 20 Department of Pathology, University of Cambridge, Cambridge, UK; 21 Laboratory of Lymphocyte Signaling and Development, Babraham Institute, Cambridge, UK; 22 Department of Biomedical Engineering, Yale University, New Haven, CT USA; 23 College of Medical Science and Technology, Taipei Medical University, Taipei, Taiwan

**Keywords:** Immunology, Cancer therapy, Adaptive immunity

## Abstract

The accumulation of depolarized mitochondria commits T cells to exhaustion^1–3^, yet the precise mechanism remains unclear. Here we find that exhausted CD8^+^ T cells increase proteasome activity owing to the accumulation of depolarized mitochondria, which drives the selective degradation of mitochondrial proteins and the release of regulatory haem through haemoprotein breakdown. In turn, increased regulatory haem disrupts BACH2-mediated transcriptional regulation, thereby exacerbating T cell exhaustion and compromising stemness-like properties. Inhibition of nuclear import of regulatory haem prevents BACH2 degradation and enhances the anti-tumour efficacy of antigen-specific T cells. We find that the therapeutic efficacy of human CD19^+^ chimeric antigen receptor (CAR)-T cells in patients with B cell acute lymphoblastic leukaemia negatively correlates with the proteasome gene signature in their CAR-T cells. Manufacturing CAR-T cells in the presence of bortezomib, an FDA-approved proteasome inhibitor, prevents T cell exhaustion and improves therapeutic efficacy. Our findings identify a proteasome-guided haem signalling axis, governed by mitochondrial integrity, as a regulator of CD8^+^ T cell exhaustion and propose innovative therapeutic strategies that exploit this pathway to optimize adoptive cellular immunotherapy.

## Abstract (web summary)

Exhausted CD8^+^ T cells increase proteasome activity due to the accumulation of depolarized mitochondria, which drives the selective degradation of mitochondrial proteins and releases of regulatory haem through haemoprotein breakdown.

## Main

In response to tumours and chronic viral infections, CD8^+^ T cells undergo differentiation to form terminally exhausted T (T_TEX_) cells, which are characterized by declined effector functions, diminished responsiveness to immune checkpoint blockades, reduced proliferative ability and sustained expression of inhibitory receptors^4–6^. By contrast, progenitor exhausted T (T_PEX_) cells arise and function as a major reservoir for the expansion of effector T (T_eff_) cells, which can further differentiate into T_TEX_ cells. The commitment to T cell exhaustion is governed by dynamic transitions of transcription factor (TF) network that orchestrates the transcriptional reprogramming^7–10^. TFs, including BACH2 (ref. ^11^), T cell factor 1 (TCF-1, encoded by *Tcf7*)^12^ and forkhead box O1 (FOXO1)^13,14^, are vital for T_PEX_ cell formation and the preservation of their stem-like properties. By contrast, T_TEX_ cells downregulate stemness-associated TFs and instead express high levels of BLIMP1 (encoded by *Prdm1*)^15^ and BATF^16^, which promote terminal differentiation. Despite these observations, the specific regulatory mechanisms that modulate the transitional expression pattern of TFs that drive exhaustion commitment in tumour-reactive T cells remain unclear.

Beyond chronic antigen exposure, the tumour microenvironment (TME) exposes infiltrating T lymphocytes to severe metabolic stress, including hypoxia, glucose deprivation and immunosuppressive metabolites^17,18^. Tumour-infiltrating lymphocytes (TILs) then adapt their metabolic programs and preferences to match their bioenergetic demands. Notably, tumour-specific CD8^+^ TILs accumulate depolarized mitochondria under metabolic crises and fail to eliminate them through mitophagy^1^. Emerging evidence suggests that the accumulation of depolarized mitochondria actively instructs gene transcription and chromatin remodelling, thereby promoting terminal differentiation of CD8^+^ T cells^1–3^. Nevertheless, how accumulation of depolarized mitochondria contributing to exhaustion and impairing stemness remains largely unclear. Here we integrated proteomics and transcriptomics to investigate the effects of accumulating depolarized mitochondria on driving differentiation and engaging homeostatic mechanisms in CD8^+^ T cells. We demonstrate that the accumulation of depolarized mitochondria enhances proteasome activity, leading to the preferential degradation of mitochondrial haemoproteins. This process causes an aberrant increase in regulatory haem (RH) levels, which in turn rewires TF expression and reshapes the transcriptome, ultimately enforcing terminal exhaustion in CD8^+^ T cells. Importantly, an elevated proteasome signature in CD19 chimeric antigen receptor (CAR)-T cells correlates with poor therapeutic outcomes in patients with B cell acute lymphoblastic leukaemia (B-ALL). Furthermore, we provide proof-of-concept evidence that the proteasome inhibition can enhance CAR-T cell resistance to exhaustion commitment. Together, these findings reveal a link between mitochondrial dysfunction, proteasome-mediated regulation and transcriptional programming during T cell exhaustion, offering insights into exploit metabolic rewiring to improve T-cell-based anti-tumour immunity.

## The proteasome controls T cell exhaustion

**Fig. 1:**
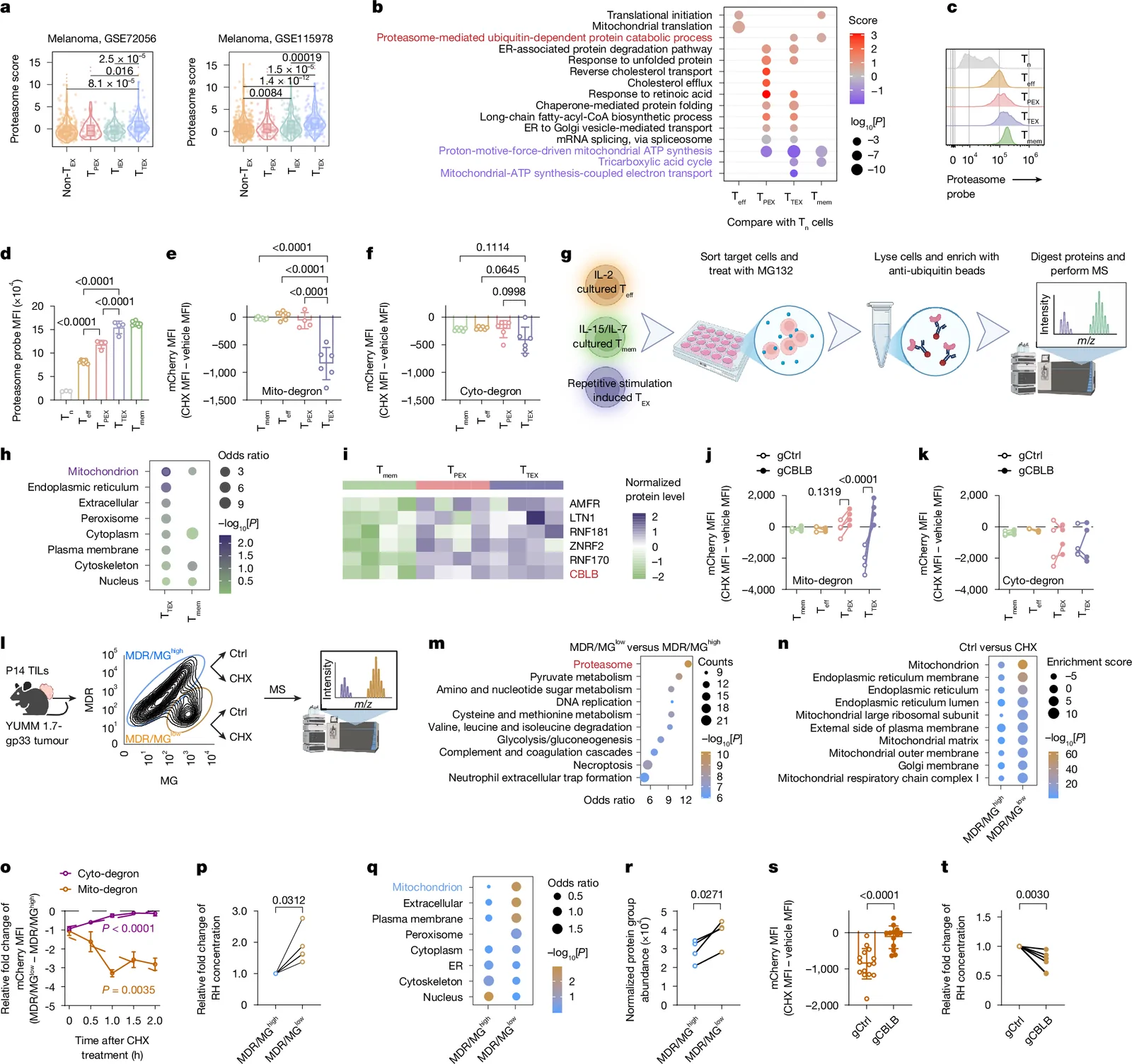
Proteasome-mediated mitochondrial protein degradation. **a**, Proteasome scores of T cell subsets from melanoma TIL scRNA-seq data. **b**, Enrichment analysis comparing the indicated cell subtypes with naive T (T_n_) cells, based on differentially expressed proteins. The red labels indicate proteasome-related genes. Purple labels indicate the mitochondrial activity-related genes. ER, endoplasmic reticulum. **c**,**d**, Representative flow plots (**c**) and the proteasome probe fluorescence intensity in human T cell subsets (**d**): T_n_ (*n* = 3), T_eff_ (*n* = 8), T_PEX_ (*n* = 4), T_TEX_ (*n* = 4) and T_mem_ (*n* = 8). **e**,**f**, Differences in mCherry mean fluorescence intensity (MFI) between control and CHX-treated human T cell subsets using the Mito-degron (**e**) or Cyto-degron (**f**) reporters. *n* = 6, except for T_PEX_ with Mito-degron, for which *n* = 4. **g**, The workflow for proteomic profiling of ubiquitinated proteins in the T_eff_, T_mem_ and T_TEX_ cell subsets. **h**, The predicted subcellular localization of ubiquitinated proteins in T_mem_ versus T_eff_ cell and T_TEX_ versus T_eff_ cell comparisons. **i**, E3 ligase expression in T_mem_, T_PEX_ and T_TEX_ cells, highlighting upregulation in T_TEX_ cells, based on proteomics data in **b**. **j**,**k**, mCherry MFI differences between control and CHX-treated *CBLB-*knockout human T cell subsets (gCBLB) using the Mito-degron reporter (**j**) or Cyto-degron reporter (**k**). *n* = 4. **l**, The workflow for proteomic analysis of MDR/MG^low^ and MDR/MG^high^ P14 TILs from YUMM1.7-gp33-melanoma-bearing mice. **m**,**n**, Pathway enrichment of proteins upregulated in MDR/MG^low^ versus MDR/MG^high^ P14 TILs, based on KEGG (**m**) or GO Cellular Component (**n**) gene sets. Proteasome-related pathways (**m**) and mitochondria-related pathways (**n**) are highlighted. **o**, The relative degradation of Cyto-degron or Mito-degron in MDR/MG^low^ versus MDR/MG^high^ TILs. *n* = 4, except for Mito-degron at 0.5 h and Cyto-degron at 2 h, for which *n* = 3. **p**, RH concentrations in MDR/MG^high^ and MDR/MG^low^ subsets of mouse T cells treated with the mitochondrial modulators Mdivi-1, antimycin A and oligomycin A. *n* = 6. **q**, The predicted subcellular localization of ubiquitinated proteins in MDR/MG^high^ and MDR/MG^low^ cells. **r**, *CBLB* expression based on proteomics data in **l**. *n* = 4. **s**, Mito-degron-mCherry MFI differences in CBLB-deficient MDR/MG^low^ human T cells after cycloheximide (CHX) treatment. *n* = 15 (gCtrl) and *n* = 13 (gCBLB). **t**, RH concentrations in *CBLB*-deficient MDR/MG^low^ human T cells under hypoxic tumour-conditioned stimulation. *n* = 6. The diagrams in **g** and **l** were created using BioRender; Xu, Y. https://BioRender.com/eep4h0q (**g**) and https://BioRender.com/gwq7cgp (**l**) (2026). Data represent two independent experiments (**d**–**f**) or were pooled from three (**o**) or four independent experiments (**j**,**k**,**p**,**r**–**t**). Each symbol represents one individual. Data are mean ± s.d. Statistical analysis was performed using one-way analysis of variance (ANOVA) (**d**–**f**), two-way ANOVA (**j**,**k**), simple linear regression (**o**), Wilcoxon rank-sum test (**p**), paired two-tailed Student’s *t*-tests (**r**) or unpaired two-tailed Student’s *t*-tests (**s**,**t**). T_EX_, exhausted T cells; T_IEX_, intermediate exhausted T cells. Source data

**Extended Data Fig. 1:**
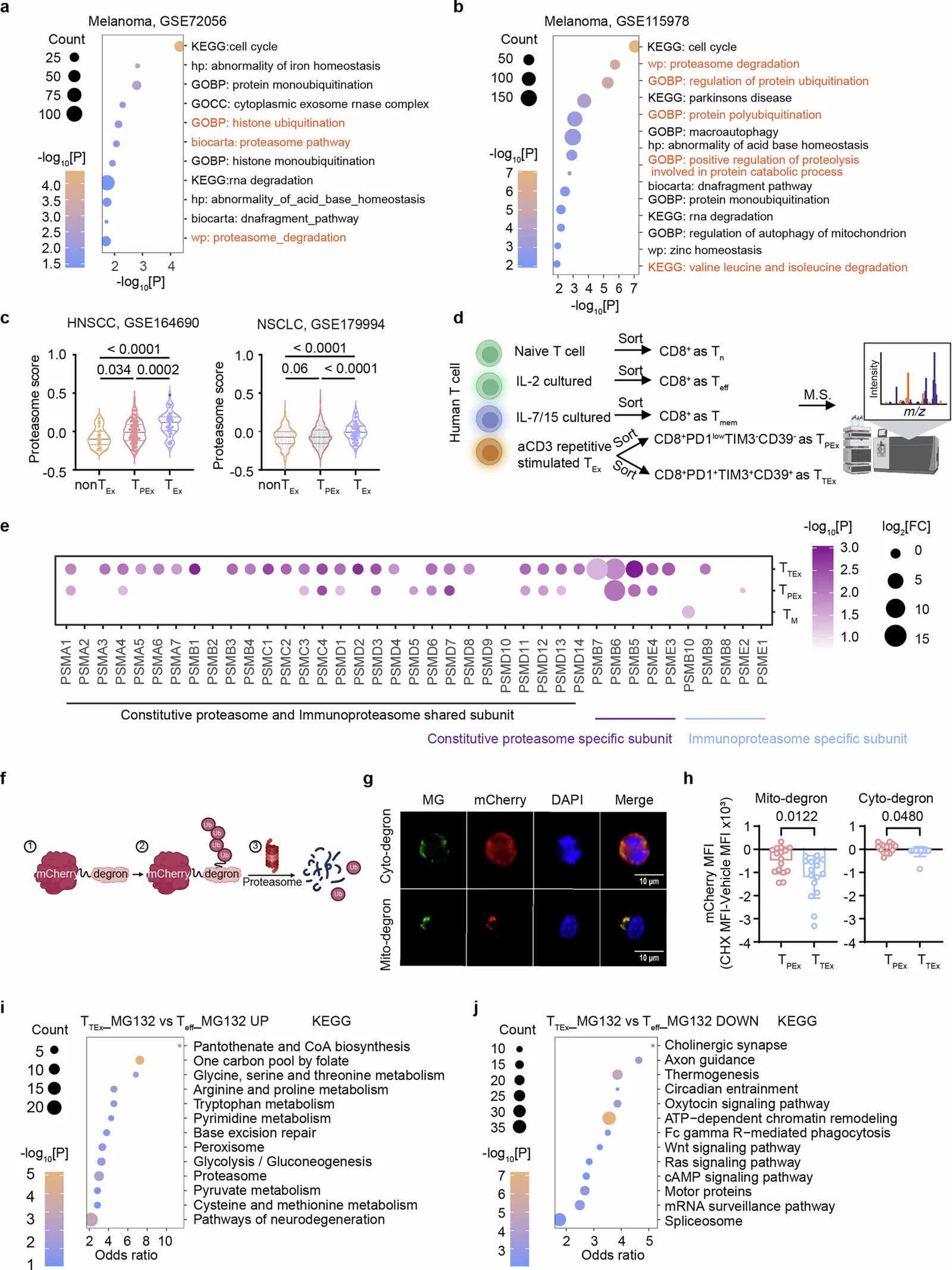
Terminally exhausted T cells engage proteasome-mediated degradation of mitochondrial proteins. **a**,**b**, Enriched MSigDB gene sets in T_TEx_ versus non-T_Ex_ melanoma TILs; proteasome-related genes are highlighted in red. **c**, Proteasome score of non-T_Ex_, T_PEx_ and T_Ex_ cells from the indicated datasets. **d**, Workflow for proteomic analysis of the indicated human T cell subsets. Created in BioRender Xu, Y. (2026), https://BioRender.com/68bs810. **e**, Fold change of proteasome subunits in T_PEx_, T_TEx_ and T_mem_ relative to T_n_, based on proteomics in (**d**). **f**, Schematic of mCherry-degron reporters. Created in BioRender Xu, Y. (2026), https://BioRender.com/9jlksue. **g**, Confocal images showing Cyto-mCherry-degron and Mito-mCherry-degron localization in CD8^+^ T cells. Scale bars, 10 μM. **h**, Difference in mCherry MFI between TCF1-eGFP^+^PD-1^+^TIM3^−^ (T_PEx_) and TCF1-eGFP^−^PD-1^+^TIM3^+^ (T_TEx_) TILs expressing Mito-degron (left) or Cyto-degron (right) reporters (Mito-degron, n = 16; Cyto-degron, n = 15). **i**, **j**, KEGG pathway enrichment of upregulated (**i**) or downregulated (**j**) proteins in T_TEx_ versus T_E_ from MG132 treated cells after ubiquitinated-protein enrichment. Each symbol represents one individual. Data are means ± s.d. and were analysed by Wilcoxon rank-sum test (**c**) or two-tailed, unpaired Student’s t test (**h**). MSigDB, Molecular Signatures Database; TIL, tumour-infiltrating lymphocyte; non-T_Ex_, non-exhausted T; T_TEx_, Terminal exhausted T; T_PEx_, Progenitor exhausted T; T_Ex_, Exhausted T. T_n_, naïve T; T_m_, memory T, T_eff_, effector T, CHX, cycloheximide. Source data

Given that terminally exhausted TILs exhibit impaired mitophagy activity^1^ and that proteins within depolarized mitochondria remain biochemically active and potentially causing detrimental effects on cells, we hypothesized that terminally exhausted CD8^+^ TILs engage alternative cellular homeostatic pathways to mitigate these harmful effects. To investigate this, we analysed publicly available single-cell RNA-sequencing (scRNA-seq) datasets of CD8^+^ TILs from patients with melanoma^19,20^ focusing on genes involved in cellular homeostasis (Supplementary Table 1). Gene Ontology (GO) analysis revealed that pathways associated with protein degradation, including the proteasome and regulation of protein ubiquitination, were significantly enriched in CD8^+^ T_TEX_ cells compared with in T_PEX_ cells (Extended Data Fig. 1a,b). To quantify this phenomenon, we computationally derived a proteasome score based on the global expression of proteasome-related genes across exhausted TIL subsets. This analysis demonstrated a progressive increase in proteasome score correlating with the severity of terminal exhaustion (Fig. 1a). Notably, this trend was consistent in TILs from human head and neck squamous cell carcinoma and non-small cell lung cancer (Extended Data Fig. 1c). To further confirm this observation, we acquired human naive T cells, generated multiple differentiated human CD8^+^ T cell subsets in vitro, including T_eff_, memory T (T_mem_), T_PEX_ and T_TEX_ cells, and analysed their proteomes (Extended Data Fig. 1d). Enrichment analysis of differentially expressed proteins revealed that both T_TEX_ and T_mem_ cell subsets were enriched for proteins involved in proteasome-mediated protein catabolic processes. However, T_TEX_ cells markedly reduced the expression of proteins related to tricarboxylic acid cycle and mitochondrial ATP synthesis, consistent with previous findings that T_TEX_ cells lose their mitochondrial activity^1,3^ (Fig. 1b and Supplementary Table 2). Notably, T_TEX_ cells showed increased expression of the constitutive proteasome subunits, including PSMB5, PSMB6, PSMB7, PSME3 and PSME4, compared with the T_PEX_ and T_mem_ cell subsets (Extended Data Fig. 1e). Consistently, both T_TEX_ and T_mem_ cells exhibited higher proteasome activity compared with T_PEX_ cells, as measured using a proteasome activity probe^21,22^ (Fig. 1c,d). We next investigated whether enhanced proteasome activity in T_TEX_ cells preferentially targets proteins in specific cellular compartments. We used a degron fusion strategy^23^ to track the degradation of the mCherry fluorescent protein localized either to the cytoplasm (Cyto-degron-mCherry) or mitochondria (Mito-degron-mCherry) (Extended Data Fig. 1f,g). After overexpression of these constructs in human T cells and treatment with cycloheximide to block protein synthesis, we assessed mCherry stability over time. T_TEX_ cells exhibited a significantly higher degradation rate of Mito-degron-mCherry, whereas degradation of cytoplasmic mCherry was comparable across all subsets (Fig. 1e,f). These findings indicate that T_TEX_ cells selectively enhance mitochondrial protein degradation rather than global protein turnover. We next expressed Mito-degron-mCherry or Cyto-degron-mCherry in mouse P14 T cells with the TCF-1–GFP reporter^12^ and transferred them into melanoma-bearing mice, enabling us to trace T_PEX_ cells based on GFP expression and the degradation of degron in vivo. Consistent with human T cells, mouse T_TEX_ cells (characterized by GFP^−^PD-1^+^TIM3^+^) exhibited increased degradation of Mito-degron-mCherry, but not Cyto-degron-mCherry, compared with T_PEX_ cells (characterized by GFP^+^PD-1^+^TIM3^−^) (Extended Data Fig. 1h).

To assess whether protein ubiquitination profiles are distinct between T cell subsets, we treated human T_eff_, T_mem_ and T_TEX_ cells with the proteasome inhibitor MG132, followed by enrichment of ubiquitinated proteins and proteomic analysis (Fig. 1g). Compared with T_eff_ cells, T_TEX_ cell subsets displayed greater levels of ubiquitinated mitochondrial proteins, whereas T_mem_ cells preferentially accumulated ubiquitinated cytoplasmic proteins (Fig. 1h). Notably, the mitochondrial proteins targeted in T_TEX_ cells were enriched in pathways related to amino acid and nucleotide metabolism (Extended Data Fig. 1i,j). To identify potential regulators of this selective ubiquitination process, we examined E3 ligases expression across T cell subsets. Among ligases significantly upregulated in T_TEX_ and T_PEX_ cells, CBLB, which was previously implicated in regulating mitochondrial potential^24^, emerged as a candidate mediator (Fig. 1i). Deletion of *CBLB* selectively stabilized the mito-mCherry-degron reporter in T_TEX_ cells, without affecting cytoplasmic reporter degradation or other T cell subsets (Fig. 1j,k). Collectively, these results indicate that T_TEX_ cells preferentially engage proteasome-mediated mitochondrial protein degradation as a compensatory homeostatic mechanism in response to impaired mitophagy and mitochondrial dysfunction.

## CBLB governs mitochondrial protein loss

**Extended Data Fig. 2:**
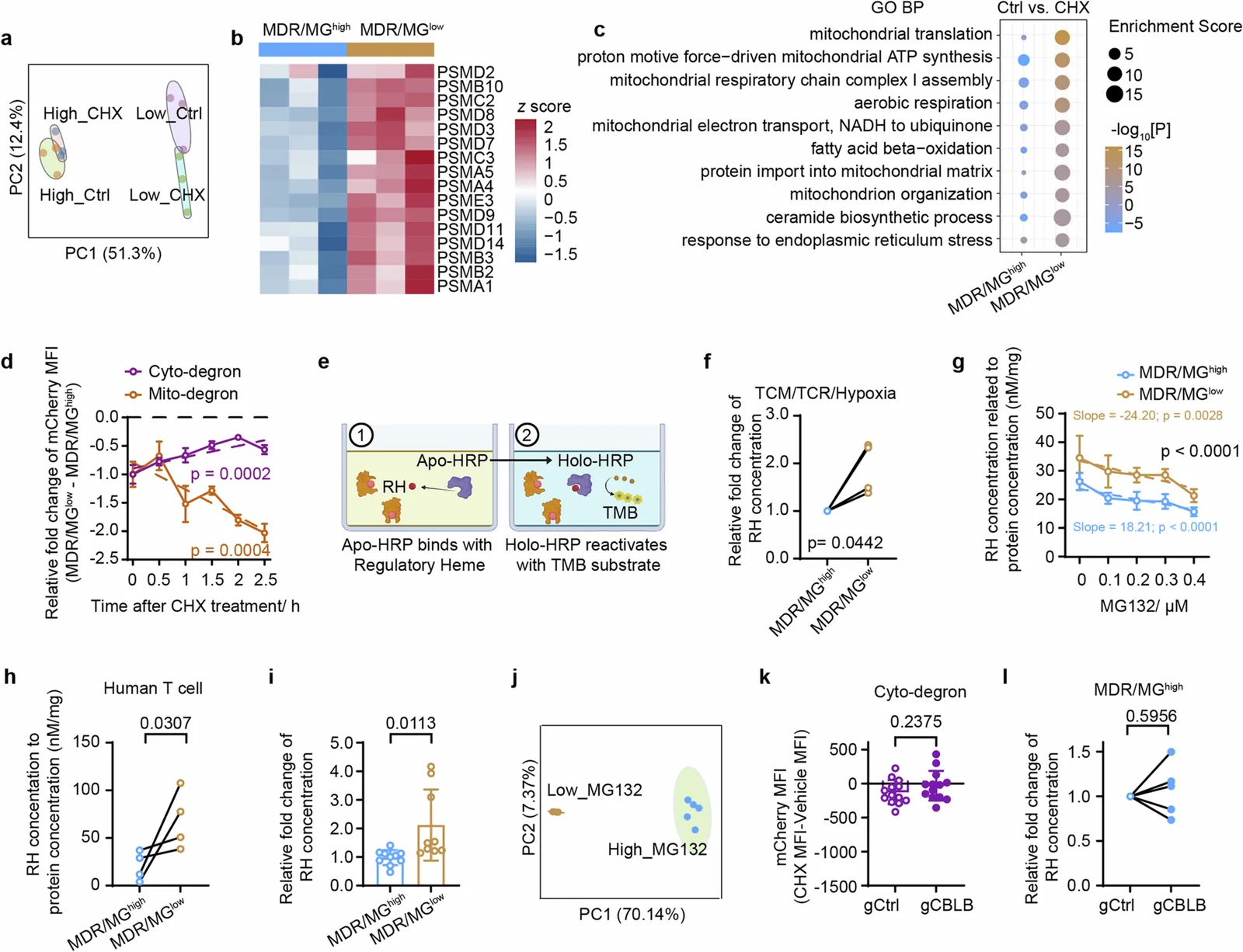
Mitochondrial depolarization induces CBL-B–dependent mitochondrial protein degradation. **a**, PCA of proteomes from the indicated groups. **b**, Heatmap of proteasome subunits differentially expressed between MDR/MG^high^ and MDR/MG^low^ T cells. **c**, Pathway enrichment of proteins upregulated in MDR/MG^low^ vs MDR/MG^high^ P14 TILs, based on GO Biological Process gene sets. **d**, Relative degradation of Cyto-degron or Mito-degron in MDR/MG^low^ vs MDR/MG^high^ (n = 3; except cyto-degron at 2 h, n = 2). **e**, Schematic of regulatory haem quantification. Created in BioRender Xu, Y. (2026), https://BioRender.com/ryadgvd. **f**, Relative RH concentration in MDR/MG^high^ and MDR/MG^low^ subsets from hypoxia tumour-conditioned stimulation (n = 4). **g**, RH concentration in MDR/MG^high^ and MDR/MG^low^ after metabolic perturbation with indicated concentration of MG132 treatment (MDR/MG^high^, 0 μM, n = 3; 0.1 μM, n = 8; 0.2 μM, n = 8; 0.3 μM, n = 12; 0.4 μM, n = 6; MDR/MG^low^, 0 μM, n = 3; 0.1 μM, n = 6; 0.2 μM, n = 6; 0.3 μM, n = 6; 0.4 μM, n = 3). **h**, Regulatory haem in MDR/MG^high^ and MDR/MG^low^ subsets from human CD8^+^ T cells expressing an NY-ESO-1-specific TCR following repeated antigen stimulation (n = 4). **i**, Regulatory haem in MDR/MG^high^ and MDR/MG^low^ TILs isolated from YUMM1.7-gp33 melanomas (three mice pooled per sample; MDR/MG^high^, n = 10; MDR/MG^low^, n = 9). **j**, PCA of proteomes from the indicated groups. **k**, Cyto-degron-mCherry MFI differences in CBLB-deficient MDR/MG^low^ human T cells following cycloheximide treatment (n = 13). **l**. Regulatory haem concentrations in CBLB-deficient MDR/MG^high^ human T cells under hypoxic tumour-conditioned stimulation (n = 5). Data are representative of two independent experiments with similar results (**d**) or pooled from two (**g**, **h**), three (**i**) or four (**f**, **k**, **l**) independent experiments. Each symbol represents one individual. Data are means ± s.d. and were analysed by Wilcoxon signed-rank test (**f,l**), simple linear regression (**d**, **g**), paired two-tailed Student’s t-test (**h**), or unpaired two-tailed Student’s t-test (**i,k**). PCA, Principal component analysis; TIL, tumour-infiltrating lymphocyte; RH, regulatory haem; MDR, MitoTracker Deep Red; MG, MitoTracker Green; T_PEx_, Progenitor exhausted T; T_IEx_, Intermediate exhausted T; T_TEx_, Terminal exhausted T. Source data

Our findings show that T_TEX_ cells engage proteasome-mediated degradation of mitochondrial proteins, leading us to hypothesize that mitochondrial depolarization initiates this process. To test this, we co-stained TILs from melanoma-bearing mice with MitoTracker Deep Red (MDR) to assess the mitochondrial membrane potential and MitoTracker Green (MG) to measure the mitochondrial mass. This enabled us to distinguish cells with depolarized mitochondria (MDR/MG^low^) from those with functional mitochondria (MDR/MG^high^)^1^. We sorted MDR/MG^high^ TILs and MDR/MG^low^ TILs for proteomic analysis and found that MDR/MG^low^ TILs increased expression of proteasome-related proteins (Fig. 1l,m, Extended Data Fig. 2a,b and Supplementary Table 3). We further analysed protein degradation by proteomic analysis. Comparison of pre- and post-MG132 treatment proteomes showed that MDR/MG^low^ TILs primarily degraded mitochondrial proteins (Fig. 1n) involved in protein importation, electron transport, structural organization and translation (Extended Data Fig. 2c and Supplementary Table 3), supporting the notion that MDR/MG^low^ TILs engage proteasome-dependent degradation of mitochondrial proteins to mitigate cellular stress. Using mCherry-degron reporters, we confirmed that MDR/MG^low^ T cells exhibited a higher degradation rate of Mito-degron-mCherry, but a lower rate of Cyto-degron-mCherry compared with MDR/MG^high^ T cells derived from their counterparts (Extended Data Fig. 2d). This preferential degradation of Mito-degron-mCherry was also observed in tumour-infiltrating P14 T cells accumulating depolarized mitochondria (Fig. 1o).

As many haemoproteins within the electron-transport chain complexes were preferentially degraded in T_TEX_ cells (Supplementary Table 4), we postulated that this process leads to a robust release of RH. We quantified RH levels in mouse and human T cells using a horseradish peroxidase (HRP)-based assay^25^ (Extended Data Fig. 2e), and found that MDR/MG^low^ T cells contained higher RH levels (Fig. 1p and Extended Data Fig. 2f). Notably, treatment with MG132 reduced RH levels, confirming that RH production results from proteasome-mediated protein degradation (Extended Data Fig. 2g). Similarly, MDR/MG^low^ T cells derived from human T cells expressing the NY-ESO-1-specific T cell receptor (TCR) after repeated antigen stimulation exhibited elevated RH levels (Extended Data Fig. 2h), as did TILs from melanoma-bearing mice (Extended Data Fig. 2i). These results suggest that T cells with depolarized mitochondria preferentially degrade mitochondrial proteins to maintain cellular homeostasis.

We further defined the specificity of protein degradation by performing proteomic profiling of MDR/MG^high^ and MDR/MG^low^ T cells after MG132 treatment, coupled with ubiquitin-enriched protein isolation (Extended Data Fig. 2j). Ubiquitinated proteins were most enriched in the mitochondrial compartment in MDR/MG^low^ T cells (Fig. 1q). We also found that CBLB was increased in MDR/MG^low^ T cells (Fig. 1r), and genetic ablation of *CBLB* reduced the degradation rate of Mito-degron-mCherry without effecting Cyto-degron-mCherry degradation (Fig. 1s and Extended Data Fig. 2k). Moreover, *CBLB* ablation reduced RH accumulation in MDR/MG^low^ T cells, but not in MDR/MG^high^ T cells (Fig. 1t and Extended Data Fig. 2l). These results demonstrate that mitochondrial depolarization in exhausted T cells triggers a CBLB-dependent, proteasome-mediated degradation of mitochondrial proteins, which is crucial for cellular homeostasis.

## Haem instructs terminal exhaustion

**Extended Data Fig. 3:**
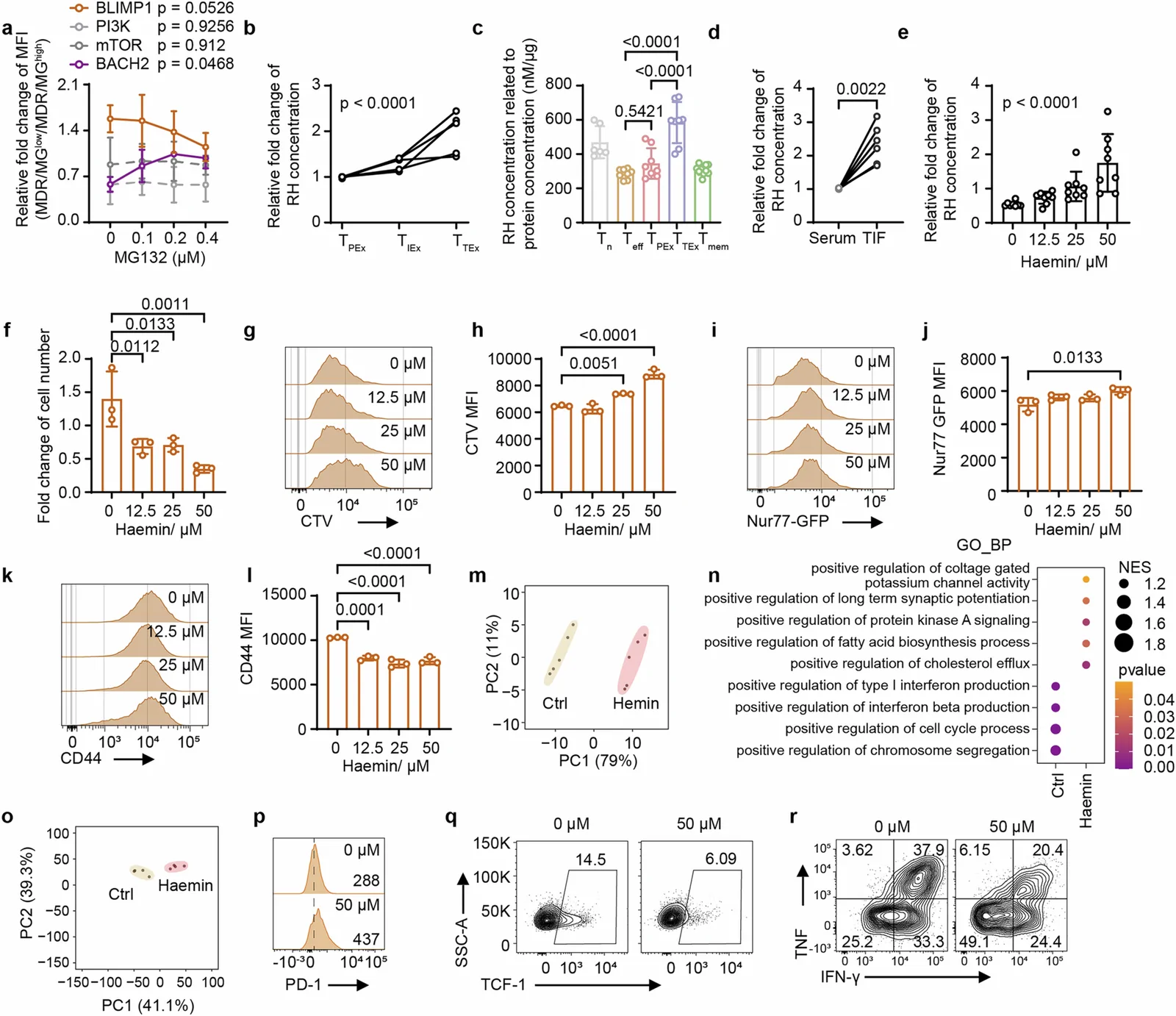
Haem released due to the mitochondrial protein degradation instructs terminal differentiation. **a**, Protein abundance of BACH2, BLIMP1, PI3K and mTOR in MDR/MG^high^ and MDR/MG^low^ subsets treated with Oligomycin A, Antimycin A, and Mdivi-1 along with indicated concentration of MG132 (n = 3, except BACH2, 0.2 μM, n = 2; 0.4 μM, n = 2). **b**, Relative RH concentration in TCF1-eGFP^+^PD-1^+^TIM3^−^ (T_PEx_), TCF1-eGFP^−^PD-1^+^TIM3^+^ (T_IEx_) and TCF1-eGFP^−^PD-1^+^TIM3^+^ (T_TEx_) P14 TILs (n = 5). **c**, RH concentration in indicated human T cell subsets (T_N_, n = 6; T_E_, n = 8; T_PEx_, n = 8; T_TEx_, n = 8; T_M_, n = 10). **d**, Relative RH concentration in TIF and paired serum from tumour-bearing mice (n = 6). **e**, Relative RH concentration in repetitively stimulated CD8^+^ T cells treated with indicated haemin concentration (n = 8). **f**, Cell expansion of repetitively stimulated CD8^+^ T cells treated with indicated haemin concentrations (n = 3). **g-l**, Representative flow plots of CTV (**g**), Nurr77-GFP (**i**) and CD44 (**k**) fluorescence intensity and the quantitative results of CTV (**h**), Nurr77-GFP (**j**) and CD44 (**l**) in repetitively stimulated CD8^+^ T cells treated with haemin (n = 3). **m**, PCA of bulk RNA-seq profiles from the indicated groups (n = 5). **n**, GO Biological Process gene set enrichment analysis of bulk RNA-seq from haemin-treated versus control cells. **o**, PCA of bulk ATAC-seq profiles from the indicated groups (n = 4). **p-r**, Representative flow plots of PD-1 (**p**), TCF-1 (**q**) and cytokine production (**r**) in expanded P14 cells from LCMV Armstrong-rechallenge. Data are representative of two (**b**, **c**) or three (**f, h, j, l**) independent experiments with similar results or are the cumulative results from three (**a, d, e**) independent experiments. Each symbol represents one individual. Data are means ± s.d. and were analysed by Wilcoxon signed-rank test (**d**), simple linear regression (**a**), or One-way ANOVA (**b, c, e, f, h, j, l**). TIF, tumour interstitial fluid; T_PEx_, Progenitor exhausted T; T_IEx_, Intermediate exhausted T; T_TEx_, Terminal exhausted T. Source data

Given that RH levels are increased in T cells with depolarized mitochondria, and mitochondrial depolarization promotes T cell exhaustion, we hypothesized that RH accumulation orchestrates the engagement of terminal exhaustion. We first examined transcriptional regulators associated with exhaustion in T cells experiencing mitochondrial stress. In MDR/MG^low^ T cells treated with MG132, BACH2 expression was restored and BLIMP1 was suppressed, whereas mTOR and PI3K levels remained unchanged (Extended Data Fig. 3a). Moreover, RH levels progressively increased with exhaustion severity in mouse CD8^+^ TILs (Extended Data Fig. 3b). Similarly, ex vivo induced human T_TEX_ cells exhibited higher levels of RH compared with T_PEX_ cells (Extended Data Fig. 3c). Notably, despite T_mem_ cells having increased proteasome activity (Fig. 1d), their RH concentration remained low, supporting the idea that T_mem_ cells do not preferentially degrade mitochondrial proteins. Moreover, tumour interstitial tissue contained higher RH levels than serum (Extended Data Fig. 3d), indicating that TILs experience increased RH in the TME. These findings reveal that mitochondrial depolarization drives mitochondrial protein degradation, increasing RH levels and altering TF expression patterns in T cells.

**Fig. 2:**
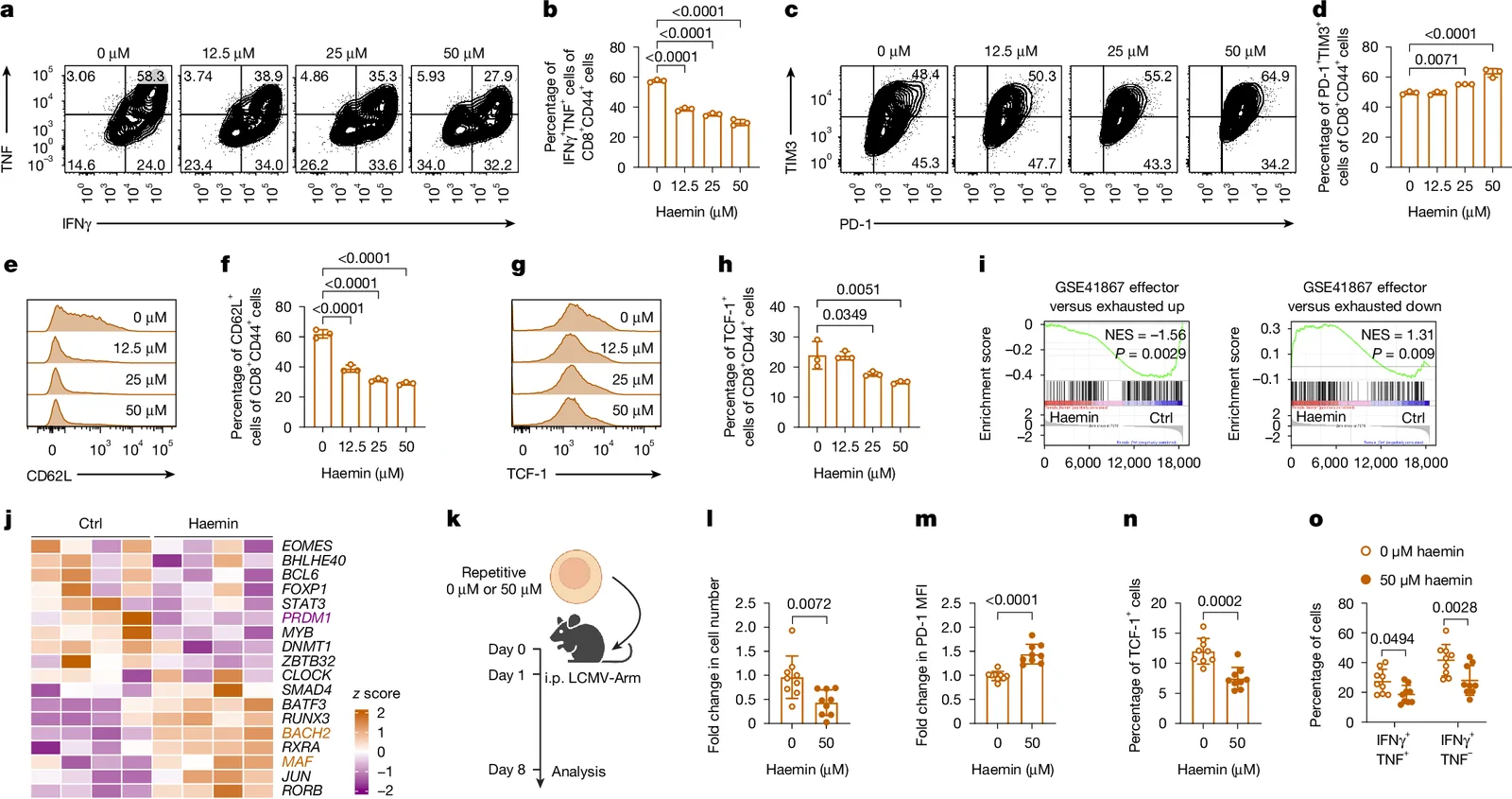
Haem exacerbates terminal differentiation of CD8^+^ T cells. **a**,**b**, Representative flow plots (**a**) and quantification (**b**) of cytokine production in repetitively stimulated T cells treated with the indicated concentrations of haemin. *n* = 3. **c**,**d**, Representative flow plots (**c**) and quantification (**d**) of PD-1 and TIM3 expression in repetitively stimulated T cells treated with the indicated concentrations of haemin. *n* = 3. **e**–**h**, Representative flow plots (**e**,**g**) and quantification (**f**,**h**) of CD62L (**e**,**f**) and TCF-1 (**g**,**h**) expression in repetitively stimulated T cells treated with haemin. *n* = 3. **i**, GSEA of exhausted T cell signature in repetitively stimulated T cells treated with 50 µM haemin compared with the control (Ctrl). **j**, Taiji analysis integrating RNA-seq and ATAC-seq data to infer transcription regulator activity in CD8^+^ T cell treated with 50 µM haemin versus the control. TFs associated with memory/stemness are shown in red, and exhaustion-associated factors are shown in blue. **k**, The experimental workflow for assessing in vivo exhaustion characteristics of CD8^+^ T cells. The diagram was created using BioRender; Xu, Y. https://BioRender.com/4uqj4i0 (2026). **l**–**o**, Total cellularity (**l**), PD-1 (**m**) and TCF-1 (**n**) expression, and cytokine-producing subsets (**o**) of expanded P14 T cells after rechallenge with LCMV-Arm (*n* = 9). Data are representative of three (**b**,**d**,**f**,**h**) independent experiments with similar results or are the cumulative results from three (**l**–**o**) independent experiments. Each symbol represents one individual. Data are mean ± s.d. Statistical analysis was performed using one-way ANOVA (**b**,**d**,**f**,**h**), two-tailed unpaired Student’s *t*-tests (**l**–**n**) and two-way ANOVA (**o**). Source data

RH modulates diverse cellular programs through haemoprotein activity^26^, leading us to hypothesize that increased RH rewires the transcriptional network governing T cell exhaustion. To test this, we treated T cells with haemin, a haem analogue that raises intracellular RH levels (Extended Data Fig. 3e). Repeatedly stimulated T cells exposed to haemin exhibited reduced proliferation and viability (Extended Data Fig. 3f–h) without impairing TCR signalling, as assessed using the Nur77–GFP reporter^27^ (Extended Data Fig. 3i,j). Notably, haemin treatment effectively suppressed IFNγ and TNF production while enhancing PD-1 and TIM3 expression (Fig. 2a–d), and concurrently downregulated CD62L, TCF-1 and CD44 (Fig. 2e–h and Extended Data Fig. 3k,l), hallmark features of progressive T cell exhaustion. Transcriptomic profiling revealed that haemin induced a gene-expression signature characteristic of T_TEX_ cells (Fig. 2i and Extended Data Fig. 3m). Pathway analysis revealed that haemin suppressed proliferation and cytokine production pathways, alongside altered metabolic processes, including cholesterol efflux^28^ and fatty acid biosynthesis^29^ (Extended Data Fig. 3n). Haemin-treated T cells also exhibited distinct chromatin accessibility patterns compared to controls, as assessed by assay for transposase-accessible chromatin sequencing (ATAC-seq) (Extended Data Fig. 3o). Integrating ATAC-seq with transcriptomic data using the Taiji analytic algorithm^30^ revealed that haemin modulated the activities of TFs central to exhaustion differentiation, including *BACH2*, *MYB*, *MAF*,* BATF*,* RUNX3* and *PRDM1* (Fig. 2j). To validate functional features, we transferred haemin-treated or control P14 T cells into mice followed with infection of Armstrong strain of lymphocytic choriomeningitis virus (LCMV-Arm) (Fig. 2k). After rechallenge, haemin-treated T cells showed reduced re-expansion, sustained exhaustion markers, declined TCF-1 expression and impaired cytokine production—features of terminal exhaustion^31^ (Fig. 2l–o and Extended Data Fig. 3p–r). These findings demonstrate that increased RH levels promote exhaustion differentiation and suppress stem-like properties in CD8^+^ T cells.

## Haem directs T cell fate through BACH2

**Fig. 3:**
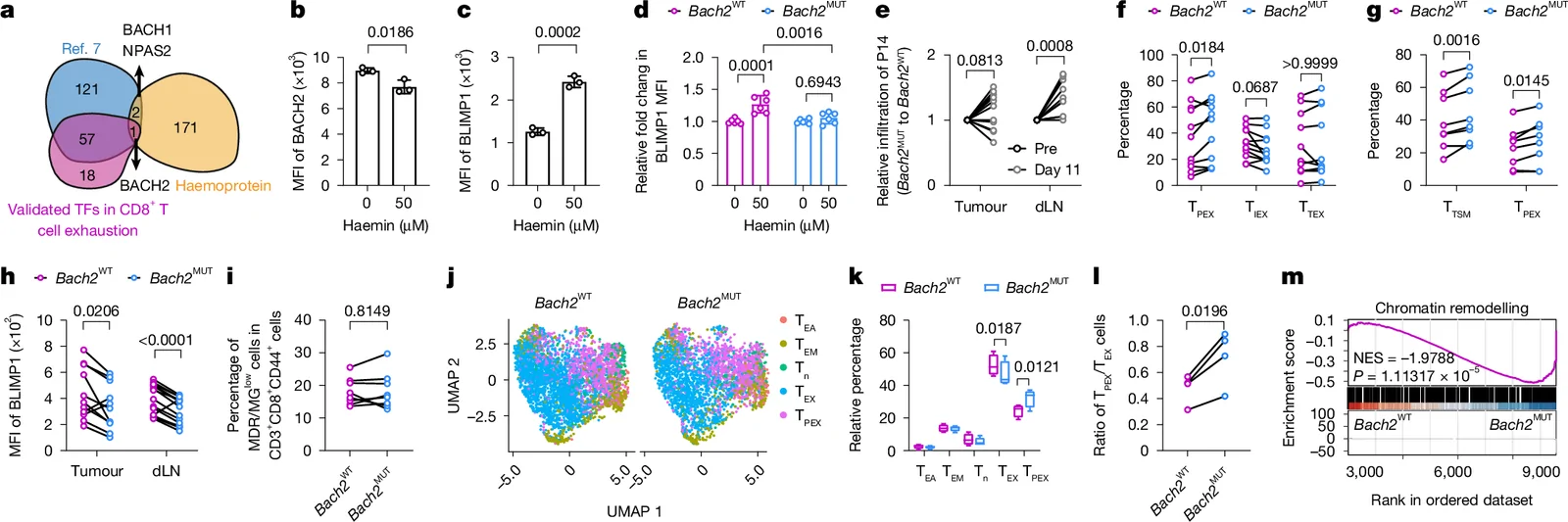
Haem drives terminal exhaustion through the BACH2–BLIMP1 axis. **a**, Overlap among haemoproteins, validated TFs and CRISPR-identified regulators of T cell exhaustion^7^. The diagram was created using BioRender; Xu, Y. https://BioRender.com/9spscv2 (2026). **b**,**c**, BACH2 (**b**) or BLIMP1 (**c**) expression in repetitively restimulated CD8^+^ T cells treated with or without haemin. *n* = 3. **d**, The relative BLIMP1 expression in repetitively restimulated P14 cells derived from P14 *Cd4*^*cre*^*Bach2*^*fl/fl*^ mice overexpressing WT (*Bach2*^WT^, *n* = 6) or mutant (*Bach2*^MUT^, *n* = 6) *Bach2*. **e**, Cellularity of *Bach2*^MUT^ versus *Bach2*^WT^ P14 cells in tumours and dLNs before (Pre) and 11 days after T cell transfer. *n* = 12 (tumour) and *n* = 11 (dLN). **f**,**g**, Differentiation states of *Bach2*^MUT^ versus *Bach2*^WT^ P14 cells in tumours and dLNs. *n* = 11 (tumour) and *n* = 8 (dLN). **h**, BLIMP1 expression in P14 TILs. *n* = 11 (tumour) and *n* = 15 (dLN). **i**, MDR/MG^low^ population frequencies in P14 TILs. *n* = 8. **j**,**k**, UMAP visualization (**j**) and cluster frequencies (**k**) of *Bach2*^MUT^ and *Bach2*^WT^ P14 TILs. *n* = 4. **l**, The ratio of T_PEX_ cells to exhausted T (T_EX_) cells (*n* = 4). **m**, GSEA of chromatin remodelling pathways. Data are representative of three (**b**–**d**) independent experiments with similar results, or pooled from two (**e**–**h**) or three (**i**) independent experiments. Each symbol represents one individual. Data are mean ± s.d. Statistical analysis was performed using two-tailed unpaired Student’s *t*-tests (**b**,**c**), two-way ANOVA (**d**), two-tailed paired Student’s *t*-tests (**i**,**l**) or multiple paired *t*-tests (**e**–**h**,**k**). T_EA_, early activated T cells; T_EM_, effector memory T cells; T_TSM_, tumour-specific memory T cells. Source data

**Extended Data Fig. 4:**
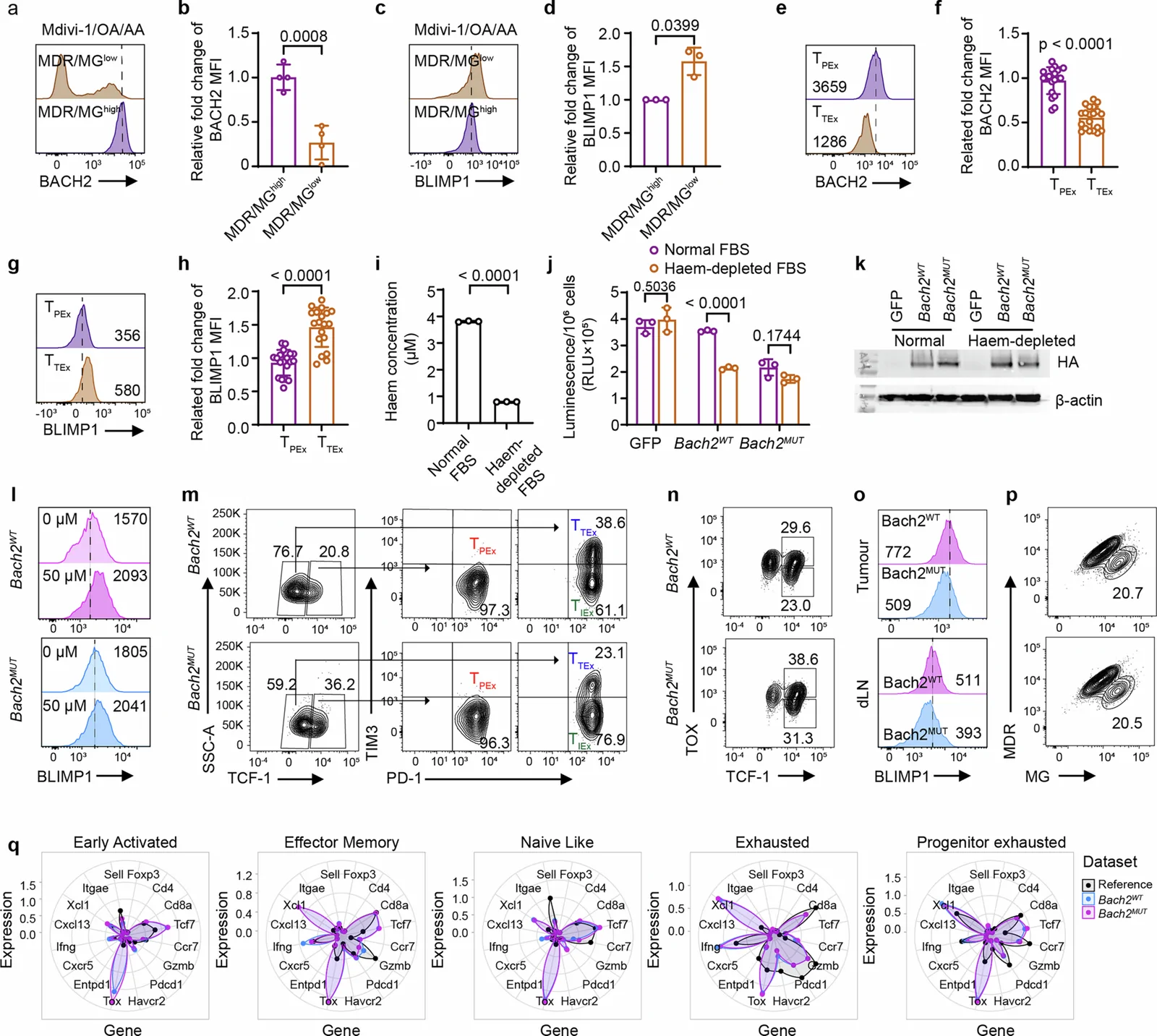
Haem drives terminal differentiation of CD8^+^ T cells via the BACH2-BLIMP1 axis. **a-d**, Representative flow plots (**a, c**) and quantitative results (**b, d**) of BACH2 and BLIMP1 in MDR/MG^low^ and MDR/MG^high^ subsets under treatment with Oligomycin A, Antimycin A, and Mdivi-1 (BACH2, n = 4; BLIMP1, n = 3). **e-h**, BACH2 (**e**, **f**) and BLIMP1 (**g**, **h**) expression in T_PEx_ and T_TEx_ P14 TILs (BACH2, n = 20; BLIMP1, n = 19). **i**, Regulatory haem concentration in normal FBS and haem-depleted FBS (n = 3). **j**, Luminescence of the indicated Jurkat reporter cells cultured in normal or haem-depleted FBS under PMA and Ionomycin stimulation (n = 3). **k**, Western blot plots of HA-tagged BACH2 and β-actin in the indicated Jurkat reporter cells cultured in normal or haem-depleted FBS. HA-tagged BACH2 and β-actin were run on the same gel as loading controls. For gel source data, see Supplementary Fig. 1. **l**, Representative flow plots show BLIMP1 in *Bach2*^*WT*^ or *Bach2*^*MUT*^ overexpressed- *Bach2*^*CKO*^ P14 cells treated with or without haemin under repetitive stimulation. **m,n**, Representative flow plots of TCF-1^+^PD-1^+^TIM3^−^ cells (T_PEx_), TCF-1^+^PD-1^+^TIM3^+^ cells (T_IEx_), and TCF-1^−^PD-1^+^TIM3^+^ cells (T_TEx_) in tumours (**m**) and TCF-1^+^TOX^−^ cells (T_TSM_) and TCF-1^+^TOX^+^ cells (T_PEx_) in dLN (**n**). **o**, Representative flow plots of BLIMP1 in *Bach2*^*WT*^ or *Bach2*^*MUT*^ P14 cells in tumours and dLNs. **p**, Representative flow plots of MDR and MG staining in *Bach2*^*WT*^ or *Bach2*^*MUT*^ P14 TILs. **q**, Average expression of subset markers across P14 TIL clusters and ProjecTILs reference datasets used for annotation. Data are representative of two (**j, k**) or three (**i**) independent experiments with similar results or are the cumulative results from three (**b, d, f, h**) independent experiments. Each symbol represents one individual. Data are means ± s.d. and were analysed by two-tailed, unpaired student’s t test (**b, d, f, h, i**) or two-way ANOVA (**j**). TIL, tumour-infiltrating lymphocyte; dLN, draining lymph node; CKO, conditional knockout; T_PEx_, Progenitor exhausted T; T_IEx_, Intermediate exhausted T; T_TEx_, Terminal exhausted T; T_TSM_, tumour specific memory T. Source data

To determine how haem promotes T cell exhaustion, we cross-referenced TFs previously reported to regulate T cell exhaustion (Supplementary Table 5) with known haemoproteins (Supplementary Table 6) and TFs identified in a CRISPR screening as modulators of exhaustion^7^. Among all candidates, BACH2 emerged as the only TF shared across these datasets (Fig. 3a), aligning with our observation that haemin treatment alters BACH2 transcriptional activity (Fig. 2j). Previous studies have reported that haem binding induces conformational changes in BACH2 that reduce its DNA-binding and transcriptional repression activity, leading to its degradation and subsequent upregulation of BLIMP1 in B cells^32^. Consistent with this, T cells accumulating depolarized mitochondria (MDR/MG^low^) exhibited reduced BACH2 and increased BLIMP1 expression compared with MDR/MG^high^ cells (Extended Data Fig. 4a–d). Similarly, T_TEX_ cells expressed lower BACH2 but higher BLIMP1 levels compared with T_PEX_ cells (Extended Data Fig. 4e–h). Haemin treatment also reduced BACH2 expression while increasing BLIMP1 expression in T cells (Fig. 3b,c). Given that the transition from T_PEX_ to T_TEX_ cells is characterized by declining BACH2 and increasing BLIMP1^11,15,33^, we speculated that RH released through mitochondrial protein degradation promotes exhaustion by disturbing the BACH2–BLIMP1 axis. To verify this hypothesis, we engineered a mutant variant of BACH2 with inactive haem-binding sites (*Bach2*^MUT^)^34^ and assessed its transcriptional repression using a Jurkat cell line containing an *Ifng*-promoter-driven luciferase reporter^35^. Under haem-depleted conditions, both *Bach2*^MUT^ and wild-type *Bach2* (*Bach2*^WT^) equally suppressed luciferase activity (Extended Data Fig. 4i,j). However, in haem-containing conditions, only *Bach2*^MUT^ retained repression activity, confirming resistance to haem-mediated inhibition. *Bach2*^MUT^ also remained stable in the presence of haem, whereas *Bach*^WT^ underwent degradation (Extended Data Fig. 4k). Consistently, haemin induced BLIMP1 expression in T cells overexpressing *Bach2*^WT^, but not *Bach2*^MUT^ (Fig. 3d and Extended Data Fig. 4l), demonstrating that RH alleviates BACH2-mediated suppression of BLIMP1. We next investigated whether *Bach2*^MUT^ preserves stem-like T cell properties in vivo. P14 cells expressing *Bach2*^WT^ or *Bach2*^MUT^ with distinct congenic markers were co-transferred into melanoma-bearing mice. Although both populations infiltrated tumours comparably (Fig. 3e), *Bach2*^MUT^-expressing cells maintained a higher proportion of T_PEX_ cells in both tumours and draining lymph nodes (dLNs) (Fig. 3f,g and Extended Data Fig. 4m). Tumour-specific stem-like TCF-1^+^TOX^−^PD-1^low^ P14 cells (T_TSM_)^36^ were also enriched in *Bach2*^MUT^ populations in dLNs (Fig. 3g and Extended Data Fig. 4n). Moreover, *Bach2*^MUT^-expressing P14 cells reduced BLIMP1 expression (Fig. 3h and Extended Data Fig. 4o). Notably, *Bach2*^MUT^ expression did not restore mitochondrial fitness, suggesting that abolishing the haem-binding ability of BACH2 preserves T_PEX_ cell formation even in TILs that lose their mitochondrial fitness (Fig. 3i and Extended Data Fig. 4p).

**Fig. 4:**
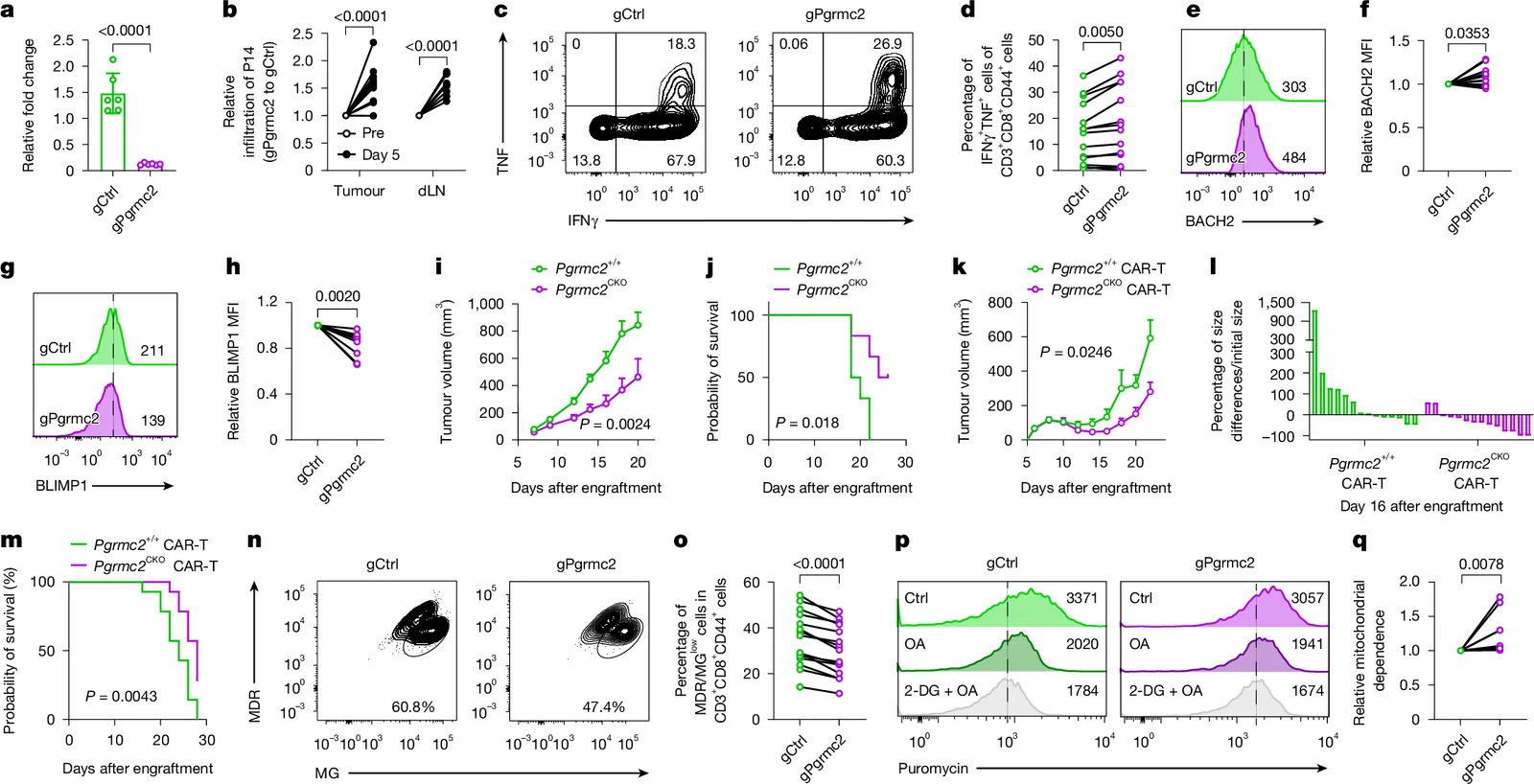
Targeting PGRMC2-guided haem redistribution restores T cell anti-tumour responses. **a**, Genomic editing efficiency of *Pgrmc2-*gRNA-transduced P14 Cas9 T cells. *n* = 6. **b**, The relative ratio of gPrgmc2 to gCtrl P14 cell infiltration into tumours (left) and dLN (right) before (Pre) and 5 days after T cell transfer; *n* = 14 (tumour) and *n* = 10 (dLN). **c**,**d**, Representative flow plots (**c**) and quantification (**d**) of IFNγ and TNF production in gCtrl and gPgrmc2 P14 TILs.* n* = 15. **e**–**h**, Representative flow plots (**e**,**g**) and quantification (**f**,**h**) of BACH2 (**e**,**f**) and BLIMP1 (**g**,**h**) expression in gCtrl and gPgrmc2 P14 TILs. *n* = 15 (BACH2) and *n* = 10 (BLIMP1). **i**,**j**, Tumour growth (**i**) and Kaplan–Meier survival curves (**j**) of YUMM1.7-gp33-melanoma-bearing mice receiving adoptive transfer of 3 × 10^6^ wild-type *Pgrmc2*^*+/+*^ or *Pgrmc2*^CKO^ P14 cells. *n* = 6. Data are mean + s.e.m. **k**–**m**, Tumour growth (**k**), tumour inhibition ratio (**l**) and Kaplan–Meier survival curves (**m**) of YUMM1.7-HER2-melanoma-bearing mice treated with *Pgrmc2*^*+/+*^ or *Pgrmc2*^CKO^ HER2 CAR-T cells. *n* = 14. Data are mean + s.e.m. **n**,**o**, Representative flow plots (**n**) and quantification (**o**) of MDR and MG staining in gCtrl and gPgrmc2 P14 TILs.* n* = 15. **p**,**q**, Representative flow plots (**p**) and quantification (**q**) of puromycin incorporation measured by single-cell energetic metabolism by profiling translation inhibition in gCtrl and gPgrmc2 P14 TILs. OA, oligomycin A. *n* = 8. Data are pooled from two (**i**–**m**), three (**a**,**b**,**d**,**h**,**o**,**q**) or four (**f**) independent experiments. Each symbol represents one individual. Data are mean ± s.d. (**a**) and mean + s.e.m. (**i**,**k**). Statistical analysis was performed using two-tailed paired Student’s *t*-tests (**d**,**o**), Wilcoxon signed-rank tests (**f**,**h**,**q**), two-way ANOVA (**b**,**i**,**k**) and log-rank tests (**j**,**m**). Source data

Next, we performed scRNA-seq analysis of P14 TILs overexpressing either *Bach2*^WT^ or *Bach2*^MUT^ and projected the data onto reference atlases using ProjecTILs^37^. Annotated T_PEX_ cells expressed stemness-associated markers such as *Tcf7* and *Ccr7*, whereas the T_EX_ cells showed the highest expression of terminal exhaustion markers, including *Pdcd1*, *Havcr2* and *Entpd1* (Extended Data Fig. 4q). *Bach2*^MUT^ TILs contained more T_PEX_ cells and fewer T_EX_ cells compared with *Bach2*^WT^ TILs (Fig. 3j–l). Although global transcriptional profiles were similar, *Bach2*^MUT^-expressing T_PEX_ cells displayed enhanced expression of chromatin remodelling genes (Fig. 3m and Supplementary Table 7). These findings demonstrate that haem orchestrates CD8^+^ T cell exhaustion and disrupts stem-like properties maintenance by binding to BACH2 and perturbing its transcriptional networks.

## Haem distribution guides exhaustion

**Extended Data Fig. 5:**
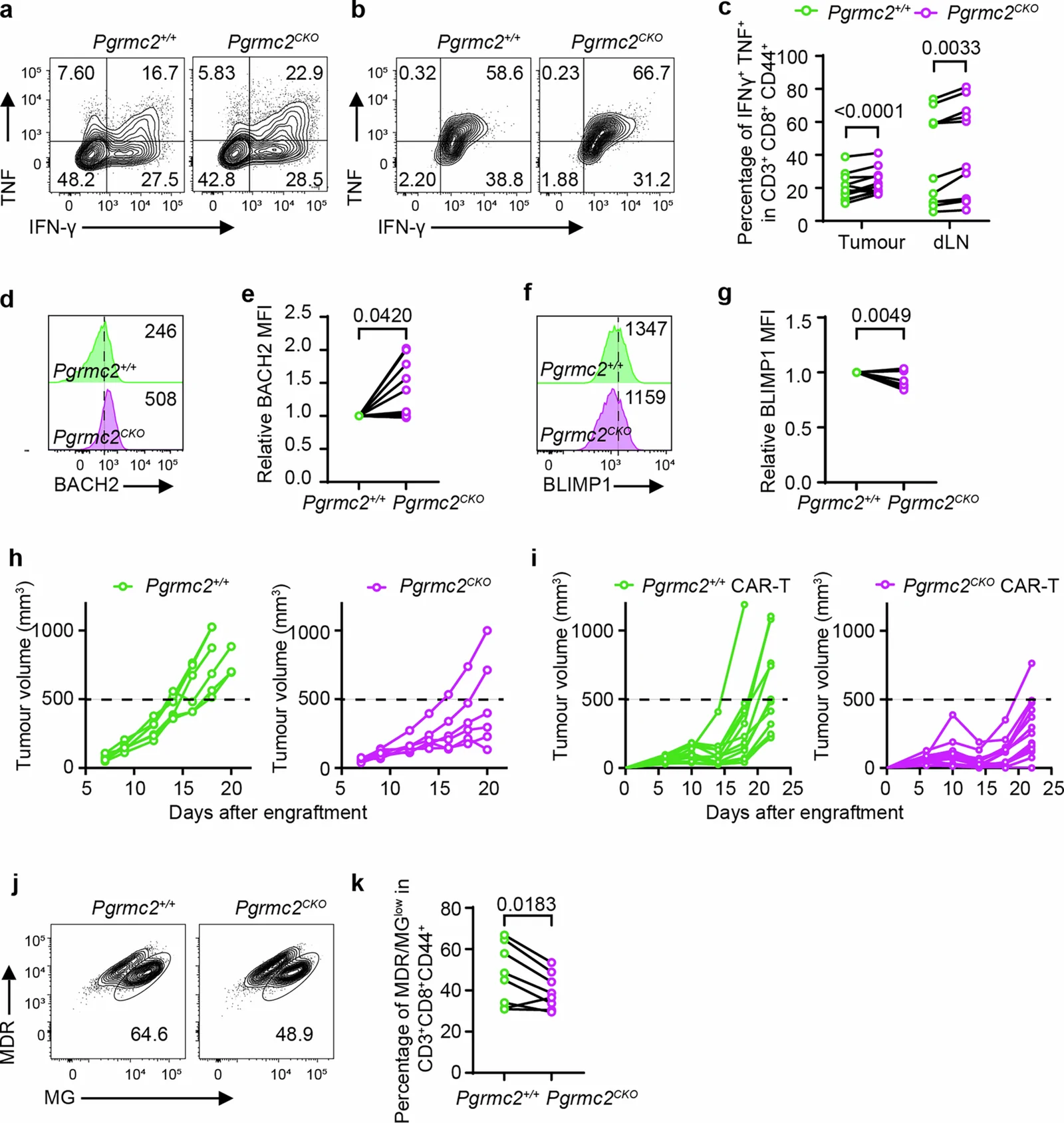
Blocking nuclear translocation of haem sustains CD8^+^ T cell effector functions. **a**, **b**, Representative flow plots of IFNγ and TNF production by *Pgrmc2*^*+/+*^ and *Pgrmc2*^*CKO*^ P14 T cells in tumours (**a**) and dLN (**b**). **c**, The quantification of IFNγ^+^TNF^+^ production in **a**, **b** (n = 11). **d-g**, Representative flow plots (**d, f**) and quantitative results (**e, g**) of BACH2 and BLIMP1 expression in *Pgrmc2*^*+/+*^ and *Pgrmc2*^*CKO*^ P14 TILs (n = 11). **h**, Tumour growth of YUMM1.7-gp33 melanoma-engrafted mice treated with *Pgrmc2*^*+/+*^ (left) or *Pgrmc2*^*CKO*^ (right) P14 T cells (n = 6). Black dotted lines indicate a tumour volume of 500 mm^3^. **i**, Tumour growth of YUMM1.7-HER2 melanoma-engrafted mice treated with *Pgrmc2*^*+/+*^ (left) or *Pgrmc2*^*CKO*^ (right) HER2 CAR T-cells (n = 14). Black dotted lines indicate a tumour volume of 500 mm^3^. **j**, **k**, Representative flow plots (**j**) and quantitative results (**k**) of MDR and MG staining in co-transferred *Pgrmc2*^*+/+*^ and *Pgrmc2*^*CKO*^ P14 TILs (n = 8). Data are pooled from two (**c, e, g-i, k**) independent experiments. Each symbol represents one individual. Data are means ± s.d. and were analysed by two-way ANOVA (**c**), Wilcoxon signed-rank test (**e, g**) or two-tailed, paired Student’s t-test (**k**). CAR, chimeric antigen receptor; TIL, tumour-infiltrating lymphocyte; dLN, draining lymph node; CKO, conditional knockout. Source data

As proteasome-mediated releases RH mainly occurs in the cytoplasm, haem translocation into the nucleus is probably required for transcriptional modulations. PGRMC2 functions as a haem chaperone that mediates haem nuclear transfer^38^, promoting us to investigate whether targeting PGRMC2 could prevent exhaustion and preserve T_PEX_ cells. To test this, P14 cells from Cas9-expressing P14 mice were transduced with either a control guide RNA (gCtrl) or a guide RNA targeting *Pgrmc2* (gPgrmc2) (Fig. 4a). Equal numbers of these cells with different congenic markers were co-transferred into melanoma-bearing mice. PGRMC2-deficient P14 cells accumulated at higher frequencies and displayed improved cytokine-producing ability compared with the controls (Fig. 4b–d), indicating improved polyfunctionality, expansion and survival of CD8^+^ TILs. Moreover, PGRMC2-deficient P14 cells displayed increased BACH2 and reduced BLIMP1 expression (Fig. 4e–h). These findings were further validated using a conditional *Pgrmc2-*knockout mouse model (*Pgrmc2*^CKO^), in which *Pgrmc2*^CKO^ P14 cells similarly enhanced cytokine production (Extended Data Fig. 5a–c), increased BACH2 and reduced BLIMP1 expression (Extended Data Fig. 5d–g). Moreover, the adoptive transfer of in vitro activated *Pgrmc2*^CKO^ P14 cells significantly delayed tumour progression and improved survival compared with wild-type P14 cells (*Pgrmc2*^*+/+*^) (Fig. 4i,j and Extended Data Fig. 5h). Likewise, HER2-specific CAR-T cells derived from *Pgrmc2*^CKO^ CD8^+^ T cells displayed superior anti-tumour efficacy compared with wild-type CD8^+^ T cells (Fig. 4k–m and Extended Data Fig. 5i). Notably, PGRMC2-deficient P14 TILs contained fewer cells with depolarized mitochondria (Fig. 4n,o and Extended Data Fig. 5j,k) and showed greater reliance on mitochondrial metabolism for ATP production, as assessed on the basis of single-cell energetic metabolism (Fig. 4p,q). Together, blocking haem nuclear translocation through *Pgrmc2* deletion restores the BACH2–BLIMP1 regulatory balance, enhances mitochondrial fitness and sustains CD8^+^ T cells effector function. Targeting the PGRMC2–haem nuclear translocation therefore represents a potential strategy to preserve T cell functionality in the TME.

**Fig. 5:**
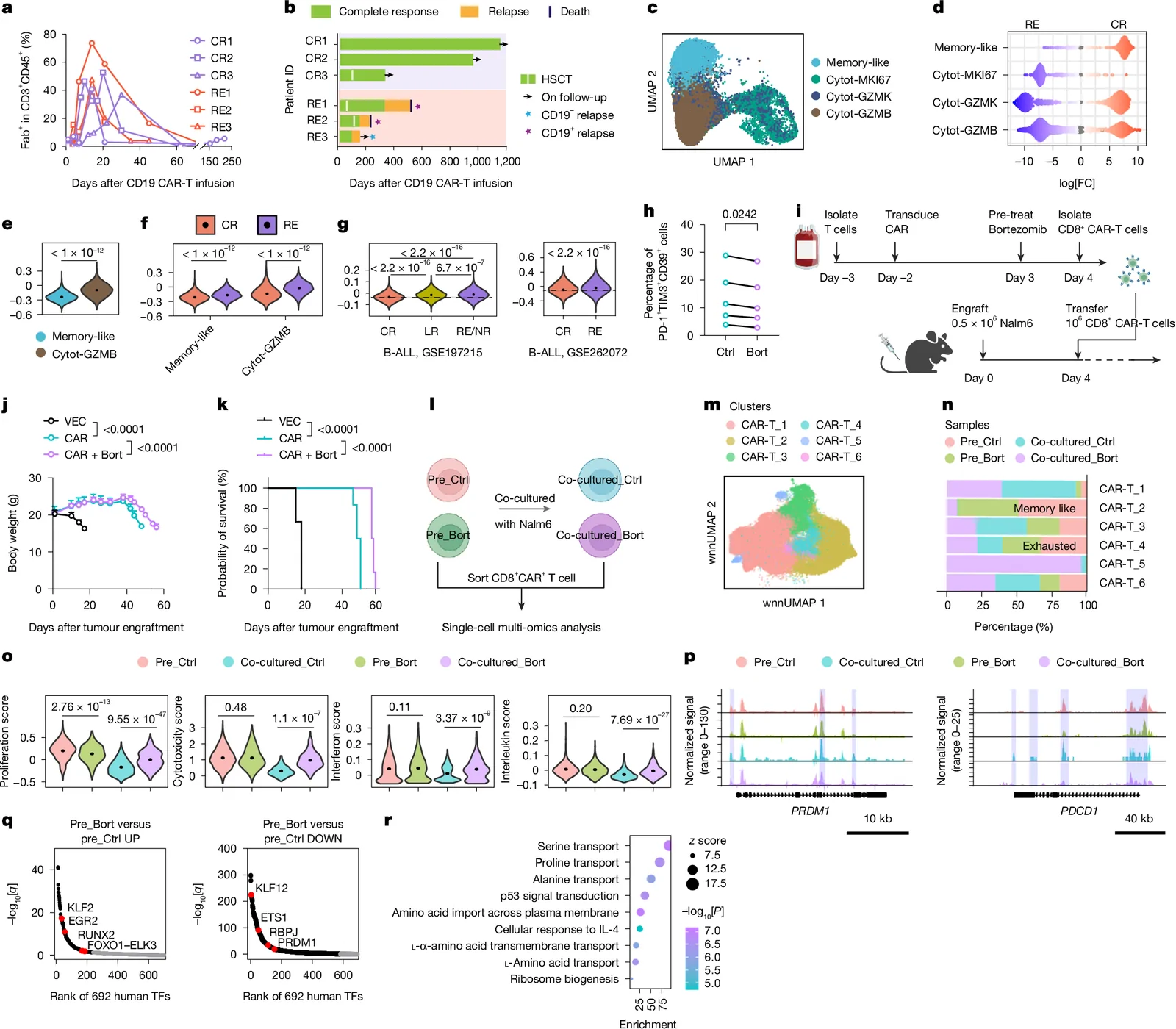
Modulation of proteasome activity enhances the therapeutic efficacy of CD19^+^ CAR-T cells. **a**, Expansion and persistence of CAR-T cells was assessed using flow cytometry. **b**, Swimmer plot showing longitudinal clinical responses. **c**, UMAP visualization of CAR-T cells isolated from peripheral blood of patients with B-ALL at days 9 and 21 after infusion. **d**, Beeswarm plot of log-transformed fold changes in cell abundance comparing complete response (CR) versus rapid relapse (RE); significant neighbourhoods at 5% false-discovery rate (FDR) are highlighted. **e**,**f**, Proteasome score in memory-like and cytotoxic (Cytot) GZMB clusters (**e**) and stratified by clinical outcome (**f**). **g**, Proteasome score of pre-infusion CAR-T cells from patients with complete response (CR), late response(LR) and rapid relapse/non-response (RE/NR) across the indicated datasets. **h**, The frequencies of PD-1^+^TIM3^+^CD39^+^ CAR-T cells after repeated rechallenge with Nalm6 cells after pretreatment with 0.1 nM bortezomib (Bort) or vehicle. *n* = 5. **i**, The experimental design for evaluating bortezomib-pretreated CAR-T cells in leukaemia-bearing mice. The diagram was created using BioRender; Xu, Y. https://BioRender.com/jev1xc8 (2026). **j**,**k**, Body weight (**j**) and Kaplan–Meier survival (**k**) of the mice treated with the indicated CAR-T cells. *n* = 6. **l**, Workflow for single-cell multi-omics analysis of CAR-T cells pretreated with bortezomib (pre_Bort) or vehicle (pre_Ctrl) and after co-culture with target cells. The diagram was created using BioRender; Xu, Y. https://BioRender.com/2e024xc (2026). **m**,**n**, Unsupervised clustering of CAR-T cells visualized by wnnUMAP (**m**) and cluster proportion (**n**). **o**, Functional gene module scores in cluster CAR-T_4. **p**, Aggregate scATAC-seq tracks for *PRDM1* and *PDCD1* in the CAR-T_4 cluster. **q**, Motif enrichment analysis of differentially accessible chromatin regions after bortezomib treatment; memory-associated (left) and exhaustion-associated (right) motifs are highlighted. **r**, GO Biological Process enrichment of cluster CAR-T_5 relative to other clusters. Data are representative of two (**j**,**k**) independent experiments with similar results, or pooled from five (**h**) independent experiments. Each symbol represents one individual. Data are mean ± s.d. Statistical analysis was performed using two-tailed, paired Student’s* t*-tests (**h**), Wilcoxon rank-sum tests (**e**–**g**,**o**), two-way ANOVA (**j**) and log-rank tests (**k**). Source data

## Proteasome targeting boosts the CAR-T cell effect

**Extended Data Fig. 6:**
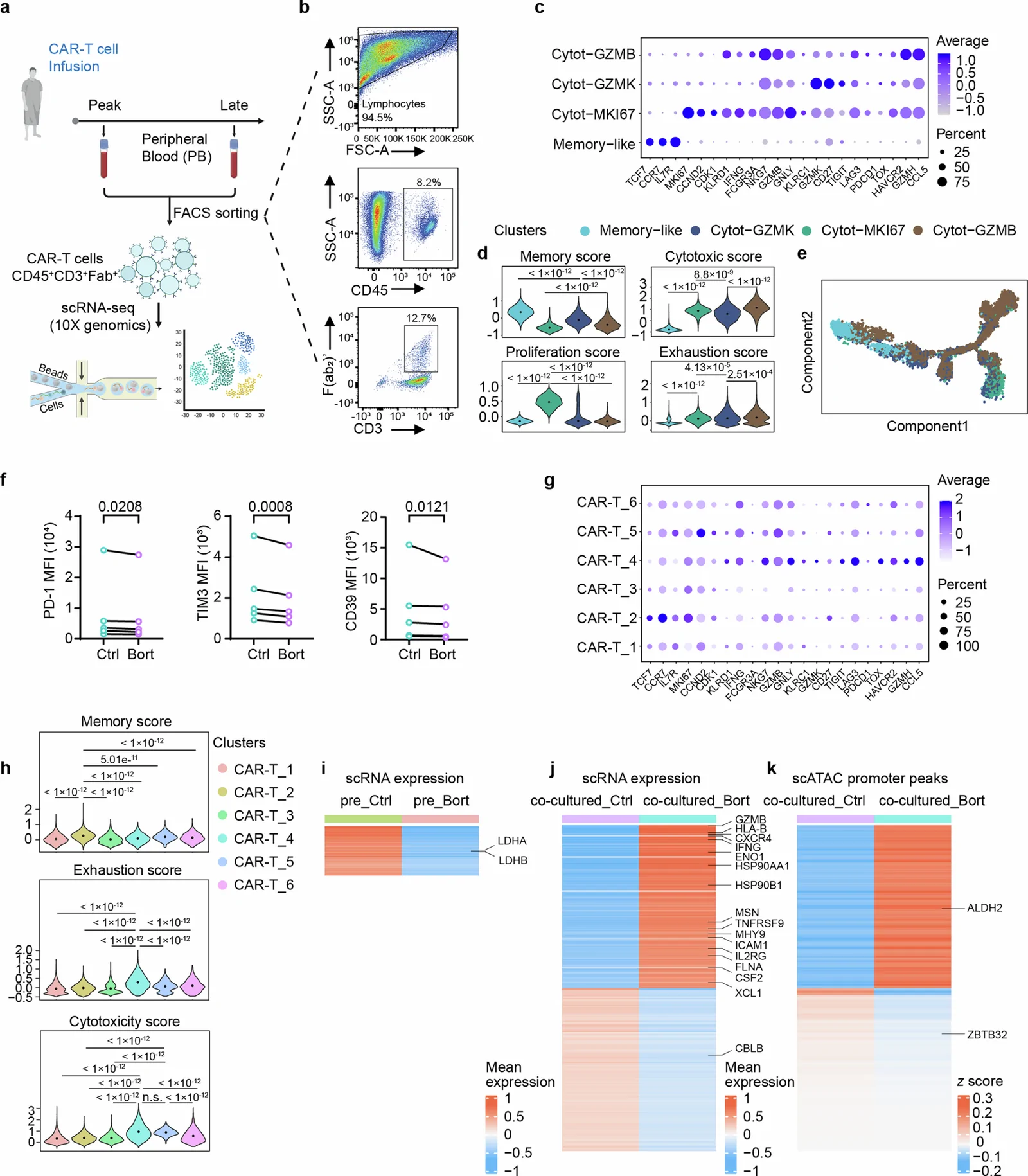
Modulation of proteasome activity enhances therapeutic efficacy of CD19 CAR T-cells. **a**, Workflow for sorting of CAR-T cells at Day 9 and Day 21 after anti-CD19 CAR-T infusion. Created in BioRender Xu, Y. (2026), https://BioRender.com/m0gieu5. **b**, Gating strategy for CAR T-cell sorting. **c**, Marker genes expression across CAR T clusters. **d**, Module scores for memory (upper left), cytotoxicity (upper right), proliferation (bottom left), and exhaustion (bottom right) across CAR T clusters. **e**, Pseudotime trajectory inferred for the indicated clusters. **f**, PD-1 (left), TIM3 (middle) or CD39 (right) expression after low-dose Bortezomib pretreatment followed by repeated Nalm6 rechallenge (n = 5). **g**-**k**, Single cell Multi-omics analysis of CAR T-cells pretreated with Bortezomib (pre_Bort) or vehicle (pre_Ctrl) and after Nalm6 co-culture (co-cultured_Bort): marker gene expression (**g**), module scores (**h**), different gene expression (**i, j**) and differential chromatin accessibility at promoters (**k**). Data are pooled from five (**f**) independent experiments. Each symbol represents one individual. Data are means ± s.d. and were analysed by two-tailed, paired student’s t test (**f**), Wilcoxon rank-sum test (**d, h**). Ctrl, Control; Bort, Bortezomib; CAR, chimeric antigen receptor. Source data

Given that proteasome-mediated degradation and subsequent haem release contribute to T cell exhaustion, we hypothesized that similar pathways may affect therapeutic outcomes of CAR-T cells. We analysed six patients with B-ALL treated with anti-CD19 CAR-T cells. No significant difference in CAR-T cell abundance within peripheral blood mononuclear cells (PBMCs) after infusion was observed between patients achieving persistent complete response and those with rapid relapse (Fig. 5a,b), suggesting that the CAR-T cell number did not correlate with the clinical outcomes. To gain deeper insights, we performed scRNA-seq analysis of 30,392 CAR-T cells isolated from patient PBMCs (Extended Data Fig. 6a,b), identifying four distinct molecular clusters (Fig. 5c). One cluster, memory-like, expressed memory-associated genes (*TCF7*, *CCR7* and *IL7R*)^19^, while the others, Cytot-MKI67, Cytot-GZMB and Cytot-GZMK, showed cytotoxic signatures (Extended Data Fig. 6c,d and Supplementary Table 8). Among these, the Cytot-GZMB cluster exhibited the highest exhaustion score (Extended Data Fig. 6d) and was positioned at a later pseudotime (Extended Data Fig. 6e), consistent with the fact that cytotoxic T cells at later differentiation stages express higher GZMB^39^. Notably, CAR-T cells from the complete response group of patients were predominately in the memory-like cluster, whereas the rapid relapse group of patients showed an enrichment in Cytot-GZMB (Fig. 5d and Supplementary Table 9), aligning with findings that memory-like T cell subsets correlate with improved therapeutic responses^40^. Similar to T_TEX_ cells (Fig. 1), the Cytot-GZMB cluster displayed a higher expression of proteasome-related genes than the memory-like cells (Fig. 5e). Both the memory-like and Cytot-GZMB clusters from the rapid relapse group of patients displayed higher proteasome activity compared with those from the complete response group of patients (Fig. 5f), suggesting that high proteasome activity may reduce CAR-T efficacy and increase terminal differentiation. Moreover, we analysed two publicly available scRNA-seq datasets of pre-infusion CD19 CAR-T from 94 patients with B-ALL and found that patients with long-term complete response had lower proteasome activity compared with patients with late relapse (complete response > 1.5 years) or patients with rapid relapse/non-response^41,42^ (Fig. 5g).

Based on these observations, we hypothesized that incorporating proteasome inhibitors during human CAR-T cell production could reduce exhaustion and enhance therapeutic efficacy. We next tested this by treating CD19 CAR-T cells with low-dose (0.1 nM) bortezomib, an FDA-approved proteasome inhibitor^43^, during the production process. CAR-T cells pretreated with bortezomib exhibited a reduced PD-1^+^TIM3^+^CD39^+^ population (Fig. 5h) and lower exhaustion markers after repeated in vitro stimulation with Nalm6 cells (Extended Data Fig. 6f). We evaluated the in vivo anti-tumour efficacy of these bortezomib-pretreated CAR-T cells in leukaemia-bearing C-NKG mice (Fig. 5i). Mice infused with bortezomib-pretreated CAR-T cells showed prolonged body weight maintenance, a key indicator of therapeutic efficacy, and significantly improved survival compared with the control group (Fig. 5j,k). To investigate the mechanism underlying the long-lasting effects of bortezomib pretreatment, we performed integrated scRNA-seq and scATAC-seq (single-cell multi-omics) analysis of bortezomib-pretreated CAR-T cells co-cultured with Nalm6 cells (Fig. 5l). We identified six distinct clusters based on their transcriptomic profiles (Fig. 5m). The ‘memory-like’ cluster (CAR-T_2) and the exhausted cluster (CAR-T_4) were defined by T-cell-related marker genes (Extended Data Fig. 6g) and scores for memory, exhaustion and cytotoxicity (Extended Data Fig. 6h). The proportion of memory-like cells was higher in the bortezomib-treated group (Fig. 5n) and, within the exhausted cluster, proliferation, cytotoxicity and cytokine scores were enhanced (Fig. 5o). Chromatin accessibility analysis showed decreased *PRDM1* and *PDCD1* chromatin accessibility in bortezomib-treated cells (Fig. 5p). While groups without co-culturing with Nalm6 cells showed minimal differences (Extended Data Fig. 6i), bortezomib-treated CAR-T cells exhibited substantial transcriptome and chromatin landscape changes after repeated antigen stimulation (Extended Data Fig. 6j,k). Integrative analysis of transcriptomic and chromatin accessibility data revealed 229 enriched TF-binding motifs, including KLF2^44^, EGR2^45^, RUNX2^46^ and FOXO1^47^, associated with memory and stem-like features were upregulated. Conversely, 587 motifs, including KLF12^48^, ETS1^7^, RBPJ^7^ and BLIMP1^33^, promoting terminal exhaustion, were downregulated (Fig. 5q). This result indicate that bortezomib pretreatment induces durable epigenetic reprogramming that enforces a memory-like transcriptional state in CAR-T cells. Notably, the CAR-T_5 cluster, a predominant cluster in bortezomib-treated group, exhibited features of amino acid import^49^ and IL-4 signalling response^42,50^, processes critical for effector function and memory formation (Fig. 5r).

Overall, we find that elevated RH, resulting from mitochondrial protein degradation, promotes exhaustion and functional decline in CD8^+^ T cells, potentially compromising the quality of CD19 CAR-T cells. These results provide proof-of-concept evidence that modulating proteasome activity during CAR-T cell production could reduce exhaustion and substantially enhance therapeutic efficacy.

## Discussion

Proteasome activity has a critical role in CD8^+^ T cell fate decisions^21^, influencing differentiation, asymmetric division and the ability to mount effective immune responses. Despite these insights, whether proteasome activity can be selectively leveraged to orchestrate T cell exhaustion in tumour or chronic infection remains unclear. Here we find that increased proteasome activity arises from the accumulation of depolarized mitochondria in CD8^+^ T cells, in which the proteasome selectively degrades mitochondrial proteins to preserve cellular homeostasis. However, this process leads to the degradation of haemoproteins, resulting in the release of RH. Increased levels of RH in turn drives T cell exhaustion by reinforcing both phenotypic and transcriptional reprogramming through modulation of the BACH2-controlled transcriptional network. Together, these findings reveal an immune regulation axis in which haem links mitochondrial health to T cell differentiation, and highlight the therapeutic potential of targeting proteasome activity and nuclear haem transport to improve CAR-T cell durability and efficacy.

We identified BACH2 as a haem-sensitive TF regulating T cell exhaustion. Ablating the haem-binding ability of BACH2 helped to sustain T cells in a progenitor-exhausted state in the TME and a stem-like state in dLNs. However, *Bach2*^MUT^-overexpressing P14 T cells did not enhance cytokine secretion and *Bach2*^MUT^ overexpression in anti-HER2 CAR-T cells did not improve survival (data not shown), suggesting that *Bach2*^MUT^ preserves stem-like features at the expense of effector function. This aligns with a recent work showing that BACH2 overexpression blocks the acquisition of cytotoxic and effector function in T cells^51^. Thus, durable tumour control requires a balance between stemness and effector differentiation rather than stemness alone^7^. Moreover, MDR has been reported to affect mitochondrial function and dynamics^52^. Thus, the intensive use of this probe may potentially introduce unrecognized caveats.

Our results suggest that reducing intracellular haem levels may help to prevent T cell exhaustion. Potential strategies include enhancing haem degradation, exporting haem into extracellular space or inhibiting haem synthesis. Haem oxygenase, an enzyme that uses NADPH and oxygen to degrade haem into biliverdin, carbon monoxide and iron, is the central mediator of haem catabolism^53^. However, the highly hypoxic conditions of the TME may impair the activity of haem oxygenase. Moreover, active haem oxygenase may facilitate the release of iron, contributing to CD8^+^ T cell dysfunction and ferroptosis^29,54^. The FLVCR1 receptor exports haem into the extracellular space following the gradient of haem^55^, but high haem levels in tumour interstitial fluids (Extended Data Fig. 3d) may limit this mechanism in TILs from expelling RH. Inhibiting haem synthesis is also plausible, as haem is critical for electron-transport chain components and cellular energy production^56^. These considerations highlight the importance of fine-tuning haem distribution rather than reducing its total abundance. Consistent with this concept, we show that limiting nuclear haem import through PRGMC2 ablation enhances mitochondrial fitness and improves the therapeutic efficacy of adoptive cellular immunotherapy. Notably, PGRMC2 deficiency, but not *Bach2*^MUT^ expression, enhanced mitochondrial function, suggesting that haem regulates additional haem-sensitive TFs or metabolic pathways beyond BACH2 that modulate mitochondrial fitness.

Integrative Taiji analysis of haemin-treated exhausted T cells revealed altered the activity of circadian rhythm regulators^57^, including BHLH40, CLOCK and RORB, which are known to couple circadian control to mitochondrial dynamics and metabolic adaptation in response to haem availability^58^. Increased RH may therefore disrupt circadian-controlled metabolic programs required for TIL survival. Supporting this notion, proteomic analysis of MG132-treated exhausted T cells revealed increased ubiquitination of proteins involved in circadian entrainment (Extended Data Fig. 1j). PD-1 expression has also been linked to intrinsic circadian regulation^59^, although the molecular underpinnings of this connection remain largely unclear. Understanding how circadian control integrates with metabolic stress and RH accumulation of RH may open new avenues for T cell-based cancer immunotherapy.

Finally, we established a strategy to modulate proteasome activity during CAR-T cell manufacturing through brief exposure to bortezomib. This short-term treatment induced durable epigenetic remodelling and long-lasting functional benefits in vivo. However, as proteasome inhibition can impair T cell memory formation^21^, careful optimization of dose and timing will be essential. Moreover, the baseline proteasome activity varies substantially among donors, potentially influencing sensitivity to proteasome inhibition and ultimately affect therapeutic outcomes. Thus, individualized adjustment of proteasome modulation parameters may be required to achieve consistent efficacy across patient-derived CAR-T products. Notably, previous studies have shown that bortezomib enhances CD8^+^ T cell tumour infiltration and effector function by modulating TCR signal and tumour antigen expression^60^. In light of our findings, combining temporally controlled proteasome inhibition with immune checkpoint blockade may represent a promising therapeutic strategy.

## Methods

### Mice

C57BL/6J, B6 CD45.1 (*B6.SJL-Ptprca Pepcb/BoyJ*), Nur77^GFP^ (*C57BL/6-Tg(Nr4a1-EGFP/cre)820Khog/J*), *Rag1*^*−/−*^ (*B6.129S7-Rag1tm1Mom/*J), *Cas9*^*fl/fl*^ (*Rosa26-LSL-Cas9* knock-in) mice^61^ were purchased from the Jackson Laboratory. P14^+^ *Cd4*^*cre*^ mice were gifts from D. Zehn. P14^+^* Cd4*^*cre*^ Cas9^+^ mice were bred from P14^+^ *Cd4*^*cre*^ and Rosa26^LSL-Cas9^ mice. Mito-QC mice^62^ were obtained from I. G. Ganley, *Pgrmc2*^*fl/fl*^ mice^63^ from E. Saez, P14^+^ TCF-1–eGFP reporter mice^12^ from W. Held and *Bach2*^*fl/fl*^ mice from M. Kurosakiand. P14^+^ *Cd4*^*cre*^*Bach2*^*fl/fl*^ mice were bred from P14^+^ *Cd4*^*cre*^ and *Bach2*^*fl/fl*^ mice. P14^+^ *Cd4*^*cre*^*Pgrmc2*^*fl/fl*^ mice were bred from P14^+^ *Cd4*^*cre*^ and *Pgrmc2*^*fl/fl*^ mice. C-NKG (*NOD*.*Cg-Prkdc*^*scid*^*Il2rg*^*em1cya*^/*Cya*) mice were purchased from Cyagen Biotechnology. The mice were housed in the animal facility of the University of Lausanne or Institute of Hematology and Blood Diseases Hospital and were kept in individually ventilated cages at 19–23 °C, under 45–65% humidity and a 12 h–12 h dark–light cycle. All of the mice used were at 5–12 weeks of age. Experimental procedures in mouse studies were approved by the Swiss authorities (Canton of Vaud, animal protocols VD3309, VD3250, VD3528, VD3765 and VD3710) or Institutional Animal Care and Use Committee of Peking Union Medical College (KT2020061-EC-1) and performed in accordance with the guidelines from the animal facility of the University of Lausanne or Institute of Hematology and Blood Diseases Hospital. Mice were euthanized when body weight loss was beyond 15%, or the tumour area reached 1,000 mm^3^ (as a predetermined end point), or when other end point criteria reached the requirements of the animal licences.

### Cell lines

The YUMM1.7 melanoma cell line was provided by M. Bosenberg^64^. The YUMM1.7-gp33 cell line was created by introducing a lentivirus carrying gp33 peptide expression cassettes into the parental cells. The YUMM1.7-HER2 cell line was created by introducing a lentivirus carrying human HER2 expression cassettes into the parental cells. These cell lines were cultured in high-glucose-supplemented DMEM (Gibco, 11965092) with 10% FBS (Gibco, 10270-106), 100 U ml^−1^ penicillin–streptomycin (Bioconcept, 4-01F00-H), and puromycin (InvivoGen), as previously described^1^. Nalm6 cells^65^ were cultured in RPMI medium (Gibco, 61870010) with 10% FBS and 1% penicillin–streptomycin. Cell lines were regularly authenticated by short-tandem-repeat profiling. All of the cell lines were verified to be free from *Mycoplasma* contamination.

### Tumour engraftment, treatment of tumour-bearing mice and rechallenge experiments

For each experiment age- and sex-matched mice were used. Mice were randomized before treatment. Fully blinded experiments were not performed owing to personnel constraints and the practical requirements for cage labelling and staffing oversight. YUMM1.7-gp33 cells (5 × 10^5^) were subcutaneously injected into C57BL/6J mice. For evaluating the function of *Bach2*^MUT^, the in vitro activated P14 T cells overexpressed with *Bach2*^WT^ or P14 T cells overexpressed with *Bach2*^MUT^ (1–3 × 10^6^ cells) were transferred into the tumour-bearing mice 7 days after tumour engraftment. For evaluating the PGRMC2 function, the in vitro activated P14 T cells or P14 *Pgrmc2*^CKO^ T cells (1–3 × 10^6^ cells) were transferred into the tumour-bearing mice 4 or 7 days after tumour engraftment. For the CRISPR–Cas9-mediated *Pgrmc2* depletion experiment, in vitro activated P14 Cas9 CD8^+^ T cells were transduced with guide RNAs (gRNAs), followed by adoptive transfer into YUMM1.7-gp33 melanoma-bearing mice on day 7 after tumour engraftment. For the in vivo rechallenge experiments, in vitro restimulated P14 T cells, either treated with or without 50 μM haemin (Sigma-Aldrich, 51280), were isolated using lymphoprep. These treated cells were then transferred into recipient mice. After cell transfer, the mice were administered with 200,000 plaque-forming units of Armstrong strain of LCMV. The transferred cells were analysed for 8 days after administration.

For the in vivo HER2 CAR-T efficacy studies, 5-week-old mice were subcutaneously injected with 5 × 10^5^ YUMM1.7-HER2 cells. Six days after engraftment, mice were randomly allocated into two treatment groups based on tumour size. The mice then received intravenous infusion of 1 × 10^6^ *Pgrmc2*^*+/+*^-HER2 CAR-T and *Pgrmc2*^CKO^-HER2 CAR-T cells, respectively. For the in vivo CD19 CAR-T efficacy studies, 8-week-old mice were intravenously injected with 5 × 10^5^ Nalm6 cells. Four days after transplantation, mice were randomly allocated into three treatment groups based on body weight: VEC-T without bortezomib pretreatment (VEC-T-Ctrl), CAR-T without bortezomib pretreatment (CAR-T) and CAR-T with 0.1 nM bortezomib pretreatment for 24 h on day 5 after CAR transduction. The mice then received intravenous infusion of 1 × 10^6^ T cells.

### Tumour digestion and TIL isolation

Tumour was minced in RPMI medium with 2% FBS, 1% penicillin–streptomycin, 1 μg ml^−1^ DNase I (Sigma-Aldrich, D5025) and 1 mg ml^−1^ collagenase (Sigma-Aldrich, C5138), followed by digestion at 37 °C for 1 h. After digestion, the sample was filtered through a 70-μm cell strainer (Thermo Fisher Scientific, 11597522). TILs were then enriched by density gradient centrifugation (800*g*, 30 min) at 25 °C with 40% and 80% Percoll (MgBCH-Milain, 41-17-0891-01). The middle layer was collected for further analysis.

### Depolarized mitochondria induction, MDR and MG staining, and RH detection

To induce mouse T cells with depolarized mitochondria, in vitro activated CD8^+^ T cells were cultured with conditioned medium from YUMM1.7 melanoma cells in the presence of Dynabeads Mouse T-Activator CD3/CD28 (Gibco, 11453D) under hypoxic conditions (1.0% O2). For experiments involving mitochondrial uncoupler and mitophagy inhibitor treatments, in vitro activated CD8^+^ T cells were cultured in medium supplemented with 100 μM Mdivi-1 (MedChem Express, HY-15886), 10 μM oligomycin A (MedChem Express, HY-16589), and 1 μM antimycin A (Sigma-Aldrich, A8674) for 4 h. In an in vivo setting, 1 × 10^6^ in vitro activated P14 T cells were transferred into YUMM1.7-gp33-tumour-bearing mice. After 6 days, tumours were digested and prepared for further analysis.

For human CD8^+^ T cells, the induction of dysfunctional mitochondria followed procedures outlined in a published paper^66^. CD8^+^ T cells were transduced with a TCR specific for the cancer-testis antigen NY-ESO-1 and repetitively stimulated with antigen presented on tumour cells. Then, 3 days after the final restimulation, CD8^+^ T cells were collected for subsequent experiments.

For MDR/MG staining, cells were collected and stained with 10 nM MDR (Thermo Fisher Scientific, M22426) and 100 nM MG (Thermo Fisher Scientific, M7514) for 15 min at 37 °C to assess mitochondrial membrane potential and mass, respectively. Then cells were stained with surface markers and Zombie Aqua dye.

The determination of RH concentration was conducted according to protocols outlined previously^25^. In total, 8,000 MDR/MG^high^ and 8,000 MDR/MG^low^ cells were sorted into 40 μl mild lysis buffer (0.1% Triton X-100 in PBS). To construct a haemin standard curve, solutions of haemin at concentrations of 1.8125 nM, 3.625 nM, 7.25 nM, 12.5 nM and 25 nM were prepared. In a flat 96-well plate, 20 μl of the indicated haemin concentration and 20 μl of cell lysate were dispensed. Subsequently, 2 μl of 50 μM apo HRP was added to the haemin or cell lysate samples. Next, 200 μl of peroxidase TMB substrate (Vector Lab, SK-4400) was added and, after several minutes, the absorbance at 652 nm was measured using a spectrophotometer.

### Luciferase reporter assay

Jurkat *Ifng-*promoter-driven luciferase cells^35^ (gift from R. Roychoudhuri) were transduced with lentivirus carrying cDNA of *Bach2*^WT^ or *Bach2*^MUT^, separately, and GFP as control. GFP^+^ cells were sorted under the same MFI of GFP. The expression of BACH2 was confirmed using anti-HA antibodies. Haem-depleted FBS was prepared by treating FBS with 20 mM l-ascorbic acid (Sigma-Aldrich, 255564) for 16 h, followed by 24 h dialysis against PBS. Haem depletion was confirmed using the RH concentration detection assay. Cells were activated by 25 ng ml^−1^ phorbol 12-myristate 13-acetate (PMA) and 500 ng ml^−1^ Ionomycin in RPMI-1640 with normal FBS or haem-depleted FBS for 24 h. The luciferase activity was then detected using the Nano-Glo luciferase assay system (Promega, N1110).

### Plasmids, virus production and detection of gene-editing efficiency

Lentivirus and retrovirus were generated by transfecting the respective plasmids, along with psPAX2 and pMD2.G for lentivirus in HEK293T cells using Turbofect (Thermo Fisher Scientific, R0532), and with pCL-ECO for retrovirus in HEK293T phoenix cells. The medium was collected at the 48 h and 72 h timepoints after transfection and underwent ultracentrifugation at 22,500 rpm for 2 h for lentivirus and at 11,500 rpm for 2 h for retrovirus. Gene deletion in activated P14^+^* Cd4*^*cre*^ Cas9^+^ T cells was accomplished through CRISPR–Cas9 using a custom gRNA retroviral vector or a dual-gRNA retroviral vector, as previously described^67^. The gRNAs were designed using the CRISPick tool, and their sequences are provided below^68^. *Pgrmc2* gRNA 1, AAGTCCCGCTTCTTCATGCG; *Pgrmc2* gRNA 2: TATGGCATCTTTGCTGGCAG; *Pgrmc2* gRNA 3, CCAGCGCACCCACAGCCGGT. Three distinct gRNAs targeting the same gene were combined during concentration using an ultracentrifuge and subsequently frozen for future use. To evaluate gene deletion efficiency, T cell genomic DNA extracted at day 6 after gRNA transduction using DNeasy Blood & Tissue Kits (Qiagen, 69504) was subjected to qPCR. The assessment used 5 ng of genomic DNA and a primer set that included the gRNA itself and a primer positioned approximately 150 bp downstream of the initial primer, according to a previously published method^69^. Genome editing of *Pgrmc2* was assessed using a pair of primers: F-2, 5′-TATGGCATCTTTGCTGGCAGG; and primer R-2, 5′-GTGGAAAGGAAAACAGACTGTGTCC.

For *Bach2*^WT^ overexpression in T cells, the retrovirus-based vector, pMIT-BACH2, was provided by R. Roychoudhuri. Three mutation points of the haem-binding site of wild-type *Bach2* (*Bach2*^MUT^)^34^ were generated by overlap PCR. The Cys369 residue was mutated to Ala using the following primers: C369A-F, 5′-CAACCCTTAGTCAGGAGCTCAGCGGCCCCTTTCAACAAGGGGATCTCTCAGG-3′ and C369-R: 5′-CCTGAGAGATCCCCTTGTTGAAAGGGGCCGCTGAGCTCCTGACTAAGGGTTG-3′. The Cys499 and Cys506 residues were both mutated to Ala using the following primers: C499A/C506A-F, 5′-CAGGAAGGATACGGCCCAACACCAGCGCCCCGGTGCCAATCAAAGTCGCTCCTCGCTCACCTCCGCTCGAGACC-3′; and C499A/C506A-R, 5′-GGTCTCGAGCGGAGGTGAGCGAGGAGCGACTTTGATTGGCACCGGGGCGCTGGTGTTGGGCCGTATCCTTCCTG-3′. For *Bach2*^WT^ and *Bach2*^MUT^ overexpression in Jurkat reporter cells, the cDNA of *Bach2*^WT^ and *Bach2*^MUT^ was subcloned into the lentivirus-based vector pCDH (System Biosciences, CD526A-1).

For protein degradation reporters, the cDNA of mCherry was subcloned into pMIT or pCDH. The degron fragment was generated by annealing from a pair of primers: Degron-F, 5′-CCGGACTCAGATCTCGAGCTCAAGCTTCGAATTCTGCTTGTAAGAACTGGTT CTCTTCTTTGTCTCACTTCGTTATCCATTTGTGAC-3′; and Degron-R, 5′-AATTGTCACAAATGGATAACGAAGTGAGACAAAGAAGAGAACCAGTTCTTACAAGCAGAATTCGAAGCTTGAGCTCGAGATCTGAGT-3′. This fragment was then ligated into the mCherry plasmid for generating Cyto-mCherry-degron. The leader sequence of mitochondria specific expressed mCherry was 5′-ATGTCCGTCCTGACGCCGCTGCTGCTGCGGGGCTTGACAGGCTCGGCCCGGCGGCTCCCAGTGCCGCGCGCCAAGATCCATTCGTTG-3′. This sequence was then fused with Cyto-mCherry-degron to generate mito-mCherry-degron. The anti-CD19 CAR was generated in our previous work^65^. The *HER2* overexpression plasmid was purchased form Addgene (16257). The anti-HER2 CAR was a gift from H. Carrasco Hope.

### CD8^+^ T cell isolation, transduction, expansion and gene editing

For mouse CD8^+^ T cells, the splenocytes were collected according to the procedures published previously^67^. Total CD8^+^ T cells were isolated using the MojoSort Mouse CD8 T Cell Isolation Kit (BioLegend, 480035) according to the manufacturer’s instructions. For T cell transduction, isolated T cells were resuspended in complete T cell culture medium (RPMI, 10% FBS, 1% penicillin–streptomycin, 1× sodium pyruvate (Gibco, 11360-093), 1× l-glutamine (Gibco, 25030-024) and 0.05 mM β-mercaptoethanol (β-ME, Sigma-Aldrich, M6250)) supplemented with 10 ng ml^−1^ mouse IL-2 (mIL-2) (Peprotech, 212-12-100 μg) at 1.0 × 10^6^ cells per ml. Dynabeads Mouse T-Activator CD3/CD28 (Thermo Fisher Scientific, 11453D) were added at a ratio of two beads per cell for transduction experiments and a ratio of one to one for in vitro differentiation experiments. CD8^+^ T cell transduction was performed 24 h after activation. Retroviruses (50 to 100 μl) were placed onto RetroNectin-precoated (TAKARA, T100B) 48-well plates and centrifuged at 32 °C for 90 min at 2,000*g*. The activated CD8^+^ T cells were then collected and seeded at a concentration of 0.5 × 10^6^ cells into the well containing the virus. After centrifuging for 10 min at 1,600 rpm, the plate was placed in the incubator. Throughout the culture period, the cells were split every day at a ratio of 1 to 2.

For human CD8^+^ T cells, blood from healthy donors was collected in accordance with ethical regulations outlined in the approved protocol of the Chinese Academy of Medical Sciences and Peking Union Medical College, China. Written informed consent was obtained from the participants. PBMCs were isolated by Lymphoprep gradient (AxonLab, 12053231). CD8^+^ T cells were then isolated using the MojoSort Human CD8 T Cell Isolation Kit (BioLegend, 480129). Isolated CD8^+^ T cells were cultured in complete T cell culture medium supplemented with 10 ng ml^−1^ human IL-2 (Peprotech, 200-02) with Dynabeads Human T-Activator CD3/CD28 (Gibco, 11131D) activation at a 1:1 ratio of beads to cells for transduction. Throughout the expansion, the cell concentration was maintained at between 0.5 and 1.0 × 10^6^ cells per ml.

For the knockout of *CBLB* in human CD8^+^ T cells, two gRNAs were used: CBLBg1, AGCAAGCTGCCGCAGATCGC; and CBLBg2, CGAGGAGGAAATCCCCGAAA. Ribonucleoprotein complexes were assembled by mixing recombinant Cas9 protein (IDT, 1081059) with sgRNA at a 1:3 molar ratio (Cas9:sgRNA) and incubating the mixture at 37 °C for 5 min before electroporation. T cells were washed twice with 1× DPBS and resuspended in P3 buffer (Lonza, V4XP-3032) at the concentration recommended by the manufacturer and electroporated using the Lonza 4D-Nucleofector system (program EO-115). After electroporation, cells were resuspended in complete T cell culture medium for further experiments.

### Fluorescence-activated cell sorting analysis

TILs or in vitro induced effector CD8^+^ T cells were incubated with TruStain FcX PLUS (anti-mouse CD16/32) antibodies (BioLegend, 156604, 1:200), Zombie Aqua Fixable Viability Kit (BioLegend, 423102, 1:1,000) and surface markers at 4 °C for 30 min. The intracellular staining procedure was then performed using the FOXP3/transcription factor staining buffer set (Invitrogen, 00-5523-00) according to the manufacturer’s instructions. Fluorescence-activated cell sorting (FACS) analyses were performed using the BD LSRFortessa flow cytometer with BD FACSDiva software (v.8.0.1). FACS data were analysed using FlowJo v.10.8.1. The following antibodies were used: anti-mouse CD3e (BioLegend, 17A2, 1:100), anti-mouse CD8a (BioLegend, 53.6.7, 1:100), anti-mouse CD44 (BioLegend, IM7, 1:100), anti-mouse CD45.1 (BioLegend, A20.1, 1:100), anti-mouse CD45.2 (BioLegend, Ali4A2, 1:100), anti-mouse TCF-1 (Cell Signaling Technology, C63D9, 1:400), anti-mouse PD-1 (BioLegend, 29F.1A12 or RMP1-30, 1:100), anti-mouse TIM3 (BioLegend, RMT3-23, 1:100), anti-mouse BLIMP1 (BioLegend, 5E7, 1:200), anti-mouse BACH2 (NOVUS, NBP2-34229, 1:200), anti-mouse PI3Kγ (Cell Signaling Technology, 4252, 1:200), anti-mouse mTOR (Cell Signaling Technology, 2972, 1:200), anti-mouse CD62L (BioLegend, Mel-14, 1:100), anti-mouse IFNγ (BioLegend, XMG1.2, 1:200), anti-mouse TNF (BioLegend, MP6-XT22, 1:200), anti-human CD8a (BioLegend, SK1, 1:100), anti-human PD-1 (BioLegend, EH12.2H7, 1:100), anti-human TIM3 (BioLegend, F38-2E2, 1:100), anti-human CD39 (BioLegend, A1, 1:100) and anti-puromycin (Merck, 12D10, 1:1,000).

For cytokine staining, TILs or in vitro induced effector CD8^+^ T cells were restimulated with 50 ng ml^−1^ PMA and 1 μg ml^−1^ ionomycin or 1 μg ml^−1^ gp33 peptide in complete T cell culture medium for 4 h within the incubator. Brefeldin A (BFA, BioLegend, 420601) was introduced after 1 h of incubation. Subsequently, cells were collected and subjected to surface and intracellular staining.

### Immunoblot analysis

Every 1 × 10^6^ cells were lysed by RIPA lysis buffer^70^ supplemented with complete Protease Inhibitor Cocktail (Roche, 11697498001). After incubation on ice for 30 min, lysates were centrifuged at 15,000 rpm for 15 min at 4 °C to remove cellular debris. The same volume of samples was then processed to immunoblotting assay. The signal was detected using Supersignal West Femto Maximum Sensitivity Substrate (Thermo Scientific Scientific, 34095) with a digital imager. The following primary antibodies were used: Direct-Blot HRP anti-HA.11 epitope tag antibody (BioLegend, 901519, 1:1,000) and monoclonal anti-β-actin (Sigma-Aldrich, A2228, 1:10,000).

### Repetitive stimulation to induce exhaustion of CD8^+^ T cells ex vivo

For mouse CD8^+^ T cells, anti-CD3ε-coated (10 μg ml^−1^; BioLegend, 145-2C11) 48-well plates were prepared 1 day before restimulation. After washing three times with PBS, 1 × 10^6^ in vitro activated CD8^+^ T cells were seeded into the plates and cultured in T cell medium with or without haemin at the indicated concentrations (0 μM, 12.5 μM, 25 μM or 50 μM). After 48 h, cells were collected and prepared for further analysis. For human CD8^+^ T cells, in vitro activated human CD8^+^ T cells (1 × 10^5^ per well) were seeded into 96-well U-bottom plates. For restimulation, Dynabeads Human T-Activator CD3/CD28 beads were added at a 1:1 bead-to-cell ratio. This restimulation procedure was repeated four times at 4 day intervals. After the final stimulation, cells were collected for subsequent analysis.

### CTV staining for evaluating T cell proliferation

Cells were washed twice with PBS and then stained with 1 μM CellTrace Violet (Thermo Fisher Scientific, C34557) or CellTrace Far Red (Thermo Fisher Scientific, C34564) for 20 min at 37 °C. After staining, cells were washed twice with T cell medium and incubated under the specified conditions. The fluorescence intensity was measured by flow cytometry after 24 or 48 h of incubation.

### Proteasome activity assays

Cells were incubated with 5 μM Me4BodipyFL-Ahx3Leu3VS, a proteasome activity probe (R&D Systems, I-190-050), for 1 h at 37 °C. After incubation, cells were washed and stained with additional antibodies. The fluorescence of the probe was then measured using excitation and emission wavelengths of 515 nm and 519 nm, respectively.

### Protein degradation reporter for evaluating mitochondrial or cytoplastic protein degradation

For the mouse in vitro assay, Cyto-mCherry-degron or Mito-mCherry-degron was transduced into mouse T cells at day 1. On day 6, cells were treated with Mdivi-1, oligomycin A and antimycin A for 4 h to induce mitochondrial dysfunction, followed by CHX for 2.5 h. mCherry MFI was measured every 0.5 h. For in vivo assay, P14 cells expressing Cyto-degron or Mito-degron were transferred into YUMM1.7-gp33-melanoma-bearing mice. After 6 days, TILs were isolated and treated with CHX for 2 h. The mCherry MFI was measured every 0.5 h. The relative degradation was calculated as the MFI difference between the MDR^low^MG^low^ and MDR^high^MG^high^ subsets.

### SCENITH assay

For the single-cell energetic metabolism by profiling translation inhibition (SCENITH) assay^71^, the samples were cultured in RPMI medium supplemented with 2% FBS. TILs were seeded at a density ranging between 0.2 and 0.7 × 10^6^ cells per well and treated with inhibitors: 1 µM oligomycin A and/or 100 mM 2-deoxy-glucose (2-DG, Sigma-Aldrich, D6134). During incubation for 45 min at 37 °C, 10 µg ml^−1^ puromycin (Sigma-Aldrich, P7255) was added in the last 30 min of incubation. Subsequently, the cells were stained with surface markers and Zombie Aqua dye. Staining with anti-puromycin APC-conjugated antibody (12D10) was conducted after fixation and permeabilization of the cells for 1 h at 4 °C. Mitochondrial dependency was assessed as previously reported^71^. In brief, the MFI of untreated cells (CO), oligomycin A-treated cells (O) and oligomycin A + 2-DG-treated cells (DGO) was determined. Mitochondrial dependency was calculated using the following formula: 100 × [(CO − O)/(CO − DGO)].

### RNA-seq and data analysis

Sorted in vitro repetitive-stimulation-induced exhausted CD8^+^ T cells from the indicated conditions were collected. The total RNA was extracted using the RNeasy Micro Kit (Qiagen, 217084). The sequencing library was prepared using the illumine Truseq Stranded RNA kit and sequenced in paired-end 150 bp format on the Element Biosciences Aviti system. TrimGalore (v.0.6.6) was used for quality control and adaptor trimming. The trimmed reads were mapped to the mm10 reference genome using STAR (v.2.7.9a). The summarizeOverlaps function from the GenomicAlignments package (v.1.30.0) in R/Bioconductor (v.3.13) was applied for determining gene expression by counting the alignments to exons. Gene sets enrichment analysis (GSEA) was performed using GSEA software (v.4.3.2). Principal component analysis (PCA) was performed in R (v.4.3.2).

### Chromatin accessibility data processing and TF motif enrichment analysis

Sorted in vitro repetitive-stimulation-induced exhausted CD8^+^ T cells from the indicated conditions were collected and chromatin accessibility mapping performed using ATAC-seq, as described previously^72^. Sequencing adapters were then removed, and low-quality reads were filtered out using fastp software (v.0.20.1). These reads were aligned to the mm10 mouse reference genome assembly using Bowtie2 (v.2.4.1) using the ‘-very-sensitive’ parameter. PCR duplicates were identified using samblaster (v.0.1.24) and reads mapped to ENCODE black-listed regions were excluded before peak calling. Open chromatin regions were identified through peak calling performed by MACS2 (v.2.2.7.1), using the following options: -n-nomodel -keep -dup auto -g 2407883318 -p e-09 -extsize 147. Peaks coinciding with blacklisted regions were discarded. A consensus peak list was then created by merging ATAC-seq peaks from all samples using the BEDTools (v.2.29.2) merge command. The identification of open chromatin regions facilitated PCA and differential accessibility was quantified using DESeq2.

### Taiji integration analysis

Taiji (v.1.2.0)^30^ with the default parameters was used to integrate our bulk RNA-seq and ATAC-seq data for key TF identification. The Cis-BP database (build 2.00)^73^ was used for mouse motifs.

### Proteomics for detecting protein degradation and data analysis

To compare T cells with healthy versus dysfunctional mitochondria, MDR/MG^high^ and MDR/MG^low^ cells were sorted from YUMM1.7-gp33 TILs and then treated with 10 μg ml^−1^ CHX for 4 h. For human T cell subset analysis, T cells were activated in vitro with 10 ng ml^−1^ IL-2 and CD3/CD28 Dynabeads at a 1:1 bead-to-cell ratio. T_eff_ cells were defined as IL-2-activated cells maintained with 10 ng ml^−1^ IL-2. T_mem_ cells were IL-2-activated but maintained with 10 ng ml^−1^ IL-15 and 5 ng ml^−1^ IL-7. T_PEX_ cells (PD-1^low^TIM3^−^CD39^−^) and T_TEX_ cells (PD-1^+^TIM3^+^CD39^+^) were sorted from ex vivo restimulated exhausted T cells. The mass spectrometry analysis was performed at the ChemBioMS Proteomics platform, University of Geneva. In brief, cell pellets (2 × 10^5^ cells for each group) were lysed and digested using the phase-transfer surfactant method. Peptide samples were analysed using the Easy Nano LC-Orbitrap Fusion System with a Nanospray Flex ion source (Thermo Fisher Scientific). Peptides were separated on a homemade C18 column (3 μm particle size, 75 μm inner diameter) using a gradient of solvent B (0.1% formic acid in 100% acetonitrile) at a flow rate of 300 nl min^−1^. The gradient comprised 114 min, starting from 2% to 25% solvent B over 100 min, before ramping up to a final concentration of 75% solvent B over the remaining 14 min. Two rounds of washing followed, each involving a switch to 98% solvent B for 1 min, then 2% solvent B for 2 min. After the second wash, the system was equilibrated with 2% solvent B for 14 min. For data-independent acquisition (DIA), 38 overlapping isolation windows were used, covering the precursor mass range of 350–1,200 *m*/*z*. Full MS was conducted at a resolution of 120,000 with AGC target set at custom (normalized to 250%) and a maximum injection time of 60 ms. DIA segments were acquired at a resolution of 30,000 with AGC target set as custom (normalized to 2000%) and dynamic maximum injection time. Higher-energy collisional dissociation (HCD) fragmentation was performed with a normalized collision energy of 27%.

Raw files from DIA measurements were analysed using the directDIA workflow in Spectronaut software (Biognosys). Identification was performed using the UniProt mouse database with the default settings. Digestion enzyme specificity was set to Trypsin/P. Modifications included carbamidomethylation of cysteine as a fixed modification, and oxidation of methionine and acetyl (protein N terminus) as variable modifications. Up to two missed cleavages were allowed. The false-discovery rate (FDR) was estimated with the mProphet approach and set to 0.01 at both peptide precursor level and protein level. Cross-run normalization was done with mean peptide and precursor quantity.

To investigate the dysfunctional mitochondria induced T cell response, differentially expressed proteins between the MDR/^low^MG^low^ and MDR/MG^high^ cells were identified using the Wilcoxon rank-sum test (cut-off of log_2_[fold change] > 0.75, *P* < 0.05) and used for gene enrichment analysis using Enrichr^74–76^. To define protein degradation, all of the quantified proteins were used to perform GO CC analysis and GO BP analysis using tidyproteomics package (v.1.8.5) (https://jeffsocal.github.io/tidyproteomics/) in R. PCA was performed using R (v.4.3.1).

To determine the difference among T_n_, T_eff_, T_mem_, T_PEX_ and T_TEX_ cells, differentially expressed proteins in each subset were compared against naive cells. The pathway enrichment analysis was performed in R by using the tidyproteomics package (v.1.8.5). The pathways with *P* < 0.001 (Wilcoxon rank-sum test) were considered to be enriched pathways.

### Proteomics for detecting protein ubiquitination and data analysis

Protein ubiquitination in MDR/MG^high^ and MDR/MG^low^ T cells was assessed by treating cells with oligomycin A, antimycin A and Mdivi-1 for 6 h, followed by sorting MDR/MG^high^ and MDR/MG^low^ CD8^+^ live T cells and incubating them with 0.4 μM MG132 for 4 h. For ubiquitination analysis in the T_mem_, T_eff_ and T_TEX_ cell subsets, cells were generated as described above, and CD8^+^PD-1^−^TIM3^−^CD39^−^ T_mem_, CD8^+^PD-1^−^TIM3^−^CD39^−^ T_eff_ and CD8^+^PD-1^+^TIM3^+^CD39^+^ T_TEX_ cells were sorted and treated with 0.2 μM MG132 for 4 h.

Cells were lysed using Cell Lysis Buffer for IP (Beyotime, P0013) and incubated with BeyoMag Anti-Ubiquitin Magnetic Beads (Beyotime, P2441) overnight at 4 °C, according to the manufacturer’s instructions. Beads were washed twice with TBST, and bound proteins were eluted, digested, and desalted.

Mass spectrometry was performed by Beijing Qinglian Biotech using a Q Exactive HF-X quadrupole Orbitrap mass spectrometer (Thermo Fisher Scientific) coupled to an EASY-nLC 1200 system via a nano-electrospray ion source. DIA and raw data processing were carried out as described above.

Differentially expressed proteins among the T_mem_, T_eff_ and T_TEX_ cell subsets were identified relative to T_eff_ cells using a Wilcoxon rank-sum test (log_2_[FC] > 1.5; *P* < 0.05). Differentially expressed proteins between MDR/MG^low^ and MDR/MG^high^ cells were similarly identified using a Wilcoxon rank-sum test (log_2_[FC] > 1.5, *P* < 0.05). Subcellular localization prediction was performed using WoLF PSORT.

### scRNA-seq and data analysis

For patient sample analysis, two processed scRNA-seq datasets^19,20^ for melanoma were obtained from the GEO under accession numbers GSE72056 and GSE115978. The data analysis was performed as described previously^1^. In brief, the deconvolution method^77^ was used in R to normalize read counts across cells. Gene expression levels were quantified as the log_2_ of the four states of CD8^+^ T cells, denoted as CD8^+^ (TPM + 1). T cells were categorized into non-exhausted (*PDCD1*^−^*PRDM1*^+^*KLRG*^+^) and exhausted (*PDCD1*^+^*TCF7*^+^) cells, with HAVCR2^−^ cells designated as progenitor exhausted cells. Exhausted cells with *TCF7*^low^ and *HAVCR2*^high^ were further ranked based on the average expression of *HAVCR2*, *LAG3* and *CTLA4*. Cells ranked between 35% and 65% were considered partially exhausted, while those above 70% were classified as fully exhausted^1^. The proteasome score was calculated by the AddModuleScore^19^, the feature genes of proteasome score were from KEGG proteasome (hsa03050) pathway.

For detecting different RNA profiles between P14 *Bach2*^MUT^ and P14 *Bach2*^WT^, CD45.1/CD45.2 P14 T cells with *Bach2*^MUT^ overexpression and CD45.1 P14 T cells with *Bach2*^WT^ overexpression were transferred to YUMM1.7-gp33 melanoma-tumour-bearing mice (CD45.2). After 9 days, CD45.1/CD45.2 and CD45.1 CD8^+^ T cells were sorted from TILs (four repeats for each group). The cell multiplexing (CellPlex reagents, 10x Genomics) and single-cell library preparation were constructed using Single Cell 3′ v3.1 kit (10x Genomics) according to the manufacturer’s protocol. The constructed libraries were sequenced on the Illumina NovaSeq 6000 platform with NovaSeq 6000 S4 Reagent Kit (300 cycles). Cell Ranger (v.3.0.2, https://support.10xgenomics.com/) was used to process scRNA-seq data and generate the matrix data containing gene counts for each cell per sample. Downstream analyses, including data normalization, scaling and dimensional reduction were conducted employing Seurat v.5 pipeline^78^. For quality-control filtering, cells with fewer than 900 and more than 6,000 detected genes, fewer than 2,400 or more than 38,000 counts, lower than 0.13 or higher than 0.33 proportion of ribosomal genes, higher than 0.07 proportion of mitochondrial genes, lower than 0.8 log_10_-transformed genes per unique molecular identifier (UMI) ratio, or higher than 0.03 proportion of haemoglobulin-related genes were discarded. As a result, in total, 3,940 cells (*Bach2*^WT^, 758.9 ± 76 cells per replicate) and 3,035 cells (*Bach2*^MUT^, 985 ± 165.7 cells per replicate) were included in the analyses. Cell type annotation was performed using ProjecTILs^37^, using a mouse reference atlas for the tumour-infiltrating T cell subset only for CD8^+^ T subtypes^79^. Differentially expressed genes for each cell type between conditions were identified using pseudobulk of each cell type per sample and using a negative binomial generalized linear model by DESeq2^80^, considering the pairing the samples in the design. Fold change results were shrunk using the apeglm method^81^. Significance was determined using the Wald test, with *P* values adjusted using the Benjamini–Hochberg method to calculate the FDR. GSEA was performed on ranked genes based on −log_10_ of the adjusted *P* value and the sign of the log_2_-transformed fold change between conditions, using clusterProfiler^82^, and using the chromatin remodelling related gene sets (Supplementary Table 7). Differences in compositional data of cell types ratios between conditions were evaluated using a paired* t*-test, with *P* values adjusted using FDR correction.

For analysis of human patient CAR-T cells, peripheral blood samples were obtained from six relapsed/refractory patients with B-ALL enrolled in the Institute of Hematology and Blood Diseases Hospital, Chinese Academy of Medical Sciences and Peking Union Medical College. All of the participants signed an informed consent in accordance with the Declaration of Helsinki. Studies using patient samples were approved by the ethical advisory board of the Institute of Hematology and Blood Diseases Hospital (NCT02975687). The CD45^+^CD3^+^Fab^+^ CAR-T cells were sorted 9 days after infusion or 21 days after infusion. scRNA-seq was performed by Novogene. In brief, single-cell library preparation was constructed using Single Cell 3′ v2 kit or 5′ PE (10x Genomics) according to the manufacturer’s protocol. The constructed libraries were sequenced on the Illumina NovaSeq 6000 platform with NovaSeq 6000 S4 Reagent Kit (300 cycles). The raw data for scRNA-seq were mapped to the reference genome (GRCh38) using the Cell Ranger (v.3.0.2, https://support.10xgenomics.com/) and then generate the matrix data containing gene counts for each cell per sample. Cells with fewer than 500 and more than 7,000 detected genes were discarded and with a high fraction of mitochondrial genes (>20%) were removed. Moreover, the proportion of ribosome-related genes was controlled below 60%. The preprocessing data were normalized and scaled using the Seurat (R package, v.4.3.0) workflow. Next, the Harmony algorithm was used to remove batch effects between samples^83^. Subsequently, PCA and uniform manifold approximation and projection (UMAP) were used for dimension reduction. The marker genes about different clusters were found using FindAllMarkers(). The trajectory analysis was performed using Monocle (v.2.30.0) based on its official documentation (https://cole-trapnell-lab.github.io/monocle-release/docs/). The different feature score was calculated using AddModuleScore^19^; the feature genes used for memory, cytotoxic, proliferation and exhaustion score were used according to a previous publication^39^; and the proteosome score used the genes from the KEGG proteasome (hsa03050) pathway. All of the feature genes are summarized in Supplementary Table 8. To identify potential differences in the cellular abundance in CAR-T cells, we performed a differential abundance analysis comparing complete response with rapid relapse using the miloR package^84^. In brief, the Seurat object was converted into a SingleCellExperiment format. The standard miloR pipeline (https://github.com/MarioniLab/miloR) was then applied to the converted object. A graph was constructed, and neighbourhoods were defined using the recommended parameters. Next, distances were calculated, cells were counted according to the experimental design and differential abundance testing was performed. Finally, a beeswarm plot was generated to visualize the log-transformed fold changes in cell abundance between complete remission and rapid relapse. Differentially abundant neighbourhoods were identified at a FDR of 5% and highlighted accordingly.

### Single-cell multi-omics sequencing and data analysis

Live CD19^+^ CAR-T cells pretreated with bortezomib (pre_Bort) or vehicle (pre_Ctrl), co-cultured with Nalm6 target cells (co-cultured_Bort), and their corresponding controls (co-cultured_Ctrl) were sorted. Single-cell multi-omics sequencing was performed using SeeKGene. The nucleus was isolated using IGEPAL CA-630 (Sigma-Aldrich, I8896). RNase inhibitors (Sigma-Aldrich, 3335399001) were added to the reagents before use. The samples were cut and transferred to a 5 ml tube containing lysate, mixed and lysed for 3 min on ice, then filtered through a 40 μm cell filter (Sigma-Aldrich, BAH136800040). Nucleus count and viability was estimated using SeekMate Tinitan Fluorescence Cell Counter (SeekGene, M002C) with AO/PI reagent.

scRNA-Seq libraries and ATAC-seq libraries were prepared using the SeekOne DD Single Cell Genome Multi-omics (ATAC+RNA) Kit according to the manufacturer’s instructions (SeekGene, K02901). In brief, the steps included (1) genomic DNA fragmentation: an appropriate number of cell nuclei were mixed with Tn5 enzyme to fragment the genomic sequences. (2) Reagent mixing: after fragmentation, the nuclei were mixed with reverse transcription reagents and DNA ligation reagents, and then added to the sample wells of the SeekOne DD Chip S3 (Chip S3). (3) Bead and oil addition: barcoded hydrogel beads and partitioning oil were dispensed into the corresponding wells of Chip S3 separately. (4) Emulsion droplet generation: the cell-containing mixed reagents and barcoded hydrogel beads were encapsulated into emulsion droplets using the SeekOne Digital Droplet System. (5) cDNA and ATAC fragment generation: immediately after transferring the emulsion droplets into PCR tubes, the mixture was at 25 °C for 30 min and then at 42 °C for 60 min to obtain barcoded cDNA and ATAC fragments. (6) Pre-amplification: the cDNA and ATAC fragments were purified from the broken droplets and preamplified by seven cycles of PCR. (7) ATAC fragment enrichment: after purifying the preamplified products, half of the purified preamplified products were mixed with Truseq Read1 SeqPrimer AND Nextera Read2 SeqPrimer and subjected to seven cycles of PCR to enrich the ATAC fragments. The indexed sequencing libraries were purified using VAHTS DNA Clean Beads (Vazyme, N411-01), analysed by Qubit (Thermo Fisher Scientific, Q33226) and Bio-Fragment Analyzer (Bioptic, Qsep400), and then sequenced on the GeneMind SURFSeq 5000 platform with PE150 read length. (8) cDNA enrichment: the remaining half of the purified preamplified products was mixed with Truseq Read1 SeqPrimer and template-switching primers and subjected to eight cycles of PCR to enrich the cDNA. Subsequently, the cDNA was fragmented, end-repaired, amplified by index PCR and ligated to sequencing adaptors. The indexed sequencing libraries were purified using VAHTS DNA Clean Beads, analysed by Qubit and Bio-Fragment Analyzer, and then sequenced on the GeneMind SURFSeq 5000 platform with PE150 read length.

The raw data were processed using SeekArcTools (v.1.0.0). For scRNA-seq, barcodes and UMIs were extracted and corrected based on a whitelist, adapter sequences were trimmed and reads were aligned to the GRCh38 reference genome using STAR, followed by annotation and UMI correction to generate gene count matrices. For scATAC-seq, barcodes were similarly processed, adapter contamination was removed and reads were aligned with bwa mem. SnapATAC2^85^ was then used to generate fragment files, call peaks with MACS3, compute QC metrics and perform cell calling. The final output consisted of cell- and sample-level gene count matrices and peak count matrices, enabling subsequent multiomic downstream analysis.

For downstream analysis of scRNA-seq, quality control and preprocessing were performed using Seurat (R package, v.4.4.0). Cells with fewer than 500 or more than 6,000 detected genes, fewer than 500 or more than 30,000 RNA counts, or a mitochondrial gene fraction above 20% were removed. After normalization, scaling and identification of variable features, dimensionality reduction was performed followed by batch correction using Harmony (v.1.2.3). UMAP was then applied for visualization. Differential gene expression was analysed using FindAllMarkers() with a minimum expression threshold of 10% of cells and log_2_-transformed fold change of >0.25.

For downstream analysis of scATAC-seq, Seurat, Signac (v.1.11.0) and Harmony were used. Quality control included filtering based on duplicate removal, fragment counts, fragments per peak, fraction of reads mapping to blacklist regions, nucleosome signal and transcriptional start site (TSS) enrichment. Cells with fewer than 500 or more than 100,000 ATAC reads were excluded, and peaks between 20 and 10,000 bp were retained. TF–IDF normalization and LSI were performed for dimensionality reduction, followed by batch correction using Harmony. Differentially accessible peaks were identified using FindAllMarkers() with a minimum expression threshold of 25% of cells while controlling for detected features as a latent variable. Peaks were visualized by IGV, and motif enrichment analysis was performed using motif data from the JASPAR 2022 database.

The single-cell multi-omics datasets were integrated using the weighted nearest neighbour method, followed by UMAP for dimensionality reduction and visualization. To define subclusters within the dataset, the expression levels of key genes were visualized using a dot plot. Feature scores for memory and cytotoxicity were calculated based on selected gene sets as described above. The interferon and interleukin feature scores were derived from gene sets in the KEGG pathways (https://www.genome.jp/kegg/). A summary of all of the feature genes is provided in Supplementary Table 8. Cluster feature analysis was performed and visualized using clusterProfiler^82^ using the GO gene sets.

### Statistical analyses

Statistical analysis was performed using GraphPad Prism (v.9.0.0). For data that are not normally distributed, nonparametric tests (for example, Wilcoxon rank-sum tests or Wilcoxon signed-rank tests) were used. For non-paired data, unpaired Student’s* t*-tests or equivalent nonparametric tests were used. For paired data, paired Student’s *t*-tests were used. For data with more than two groups and one variate, one-way ANOVA was used. For data with two variates, two-way ANOVA was used. For survival curve analysis, the log-rank (Mantel–Cox) test was used. Each point represents a biological replicate, and all data are presented as the mean ± s.d. or means ± s.e.m., as indicated. *P* values are labelled in the figures. *P* < 0.05 was considered to be statistically significant.

### Reporting summary

Further information on research design is available in the Nature Portfolio Reporting Summary linked to this article.

## Online content

Any methods, additional references, Nature Portfolio reporting summaries, source data, extended data, supplementary information, acknowledgements, peer review information; details of author contributions and competing interests; and statements of data and code availability are available at https://doi.org/10.1038/s41586-026-10250-y.

## Supplementary information

Supplementary Fig. 1 Uncropped immunoblots from the Extended Data Figures. **a**, Immunoblots for Extended Data Fig. 4k of HA–BACH2 and β-actin in Jurkat cells.

Reporting Summary

Supplementary Tables Supplementary Tables 1–9.

Peer Review file

## Source data

Source Data Figs. 1–5.

Source Data Extended Data Figs. 1–6.

## Data Availability

All data generated and/or analysed in this study are publicly available. Bulk RNA-seq, ATAC-seq and scRNA-seq data from mouse samples have been deposited in the NCBI GEO database under accession numbers GSE312103, GSE312102 and GSE312100, respectively. scRNA-seq data from human patient-derived CAR-T cells and single-cell multi-omics sequencing data from healthy human donor CAR-T cells have been deposited in the GSA-Human database at the China National Center for Bioinformation/ Beijing Institute of Genomics, Chinese Academy of Sciences, under accession numbers HRA012311 and HRA014168, respectively. Source data are provided with this paper.

## References

[1] Yu, Y. R. et al. Disturbed mitochondrial dynamics in CD8^+^ TILs reinforce T cell exhaustion. *Nat. Immunol.* **21**, 1540–1551 (2020). 10.1038/s41590-020-0793-3 33020660

[2] Scharping, N. E. et al. Mitochondrial stress induced by continuous stimulation under hypoxia rapidly drives T cell exhaustion. *Nat. Immunol.* **22**, 205–215 (2021). 10.1038/s41590-020-00834-9 PMC7971090 33398183

[3] Vardhana, S. A. et al. Impaired mitochondrial oxidative phosphorylation limits the self-renewal of T cells exposed to persistent antigen. *Nat. Immunol.* **21**, 1022–1033 (2020). 10.1038/s41590-020-0725-2 PMC7442749 32661364

[4] Franco, F., Jaccard, A., Romero, P., Yu, Y. R. & Ho, P. C. Metabolic and epigenetic regulation of T-cell exhaustion. *Nat. Metab.* **2**, 1001–1012 (2020). 10.1038/s42255-020-00280-9 32958939

[5] McLane, L. M., Abdel-Hakeem, M. S. & Wherry, E. J. CD8 T cell exhaustion during chronic viral infection and cancer. *Annu. Rev. Immunol.* **37**, 457–495 (2019). 10.1146/annurev-immunol-041015-055318 30676822

[6] Blank, C. U. et al. Defining ‘T cell exhaustion’. *Nat. Rev. Immunol.* **19**, 665–674 (2019). 10.1038/s41577-019-0221-9 PMC7286441 31570879

[7] Zhou, P. et al. Single-cell CRISPR screens in vivo map T cell fate regulomes in cancer. *Nature* **624**, 154–163 (2023). 10.1038/s41586-023-06733-x PMC10700132 37968405

[8] Beltra, J. C. et al. Developmental relationships of four exhausted CD8^+^ T cell subsets reveals underlying transcriptional and epigenetic landscape control mechanisms. *Immunity* **52**, 825–841 (2020). 10.1016/j.immuni.2020.04.014 PMC8360766 32396847

[9] Utzschneider, D. T. et al. Early precursor T cells establish and propagate T cell exhaustion in chronic infection. *Nat. Immunol.* **21**, 1256–1266 (2020). 10.1038/s41590-020-0760-z 32839610

[10] Daniel, B. et al. Divergent clonal differentiation trajectories of T cell exhaustion. *Nat. Immunol.* **23**, 1614–1627 (2022). 10.1038/s41590-022-01337-5 PMC11225711 36289450

[11] Yao, C. et al. BACH2 enforces the transcriptional and epigenetic programs of stem-like CD8^+^ T cells. *Nat. Immunol.* **22**, 370–380 (2021). 10.1038/s41590-021-00868-7 PMC7906956 33574619

[12] Utzschneider, D. T. et al. T cell factor 1-expressing memory-like CD8^+^ T cells sustain the immune response to chronic viral infections. *Immunity* **45**, 415–427 (2016). 10.1016/j.immuni.2016.07.021 27533016

[13] Chan, J. D. et al. FOXO1 enhances CAR T cell stemness, metabolic fitness and efficacy. *Nature* **629**, 201–210 (2024). 10.1038/s41586-024-07242-1 PMC11062918 38600376

[14] Doan, A. E. et al. FOXO1 is a master regulator of memory programming in CAR T cells. *Nature* **629**, 211–218 (2024). 10.1038/s41586-024-07300-8 PMC11062920 38600391

[15] Rutishauser, R. L. et al. Transcriptional repressor Blimp-1 promotes CD8^+^ T cell terminal differentiation and represses the acquisition of central memory T cell properties. *Immunity* **31**, 296–308 (2009). 10.1016/j.immuni.2009.05.014 PMC2783637 19664941

[16] Zhang, X. et al. Depletion of BATF in CAR-T cells enhances antitumor activity by inducing resistance against exhaustion and formation of central memory cells. *Cancer Cell* **40**, 1407–1422.e7 (2022). 10.1016/j.ccell.2022.09.013 36240777

[17] Ho, P. C. et al. Phosphoenolpyruvate is a metabolic checkpoint of anti-tumor T cell responses. *Cell* **162**, 1217–1228 (2015). 10.1016/j.cell.2015.08.012 PMC4567953 26321681

[18] Shangguan, Y., Wang, J., Ho, P. C. & Xu, Y. CD8^+^ T cell stressors converge on shared metabolic-epigenetic networks. *Trends Endocrinol. Metab.* 10.1016/j.tem.2025.08.009 (2025). 40987696

[19] Tirosh, I. et al. Dissecting the multicellular ecosystem of metastatic melanoma by single-cell RNA-seq. *Science* **352**, 189–196 (2016). 10.1126/science.aad0501 PMC4944528 27124452

[20] Jerby-Arnon, L. et al. A cancer cell program promotes T cell exclusion and resistance to checkpoint blockade. *Cell* **175**, 984–997 (2018). 10.1016/j.cell.2018.09.006 PMC6410377 30388455

[21] Widjaja, C. E. et al. Proteasome activity regulates CD8^+^ T lymphocyte metabolism and fate specification. *J. Clin. Invest.* **127**, 3609–3623 (2017). 10.1172/JCI90895 PMC5617668 28846070

[22] Berkers, C. R. et al. Profiling proteasome activity in tissue with fluorescent probes. *Mol. Pharm.* **4**, 739–748 (2007). 10.1021/mp0700256 17708652

[23] Greussing, R., Unterluggauer, H., Koziel, R., Maier, A. B. & Jansen-Durr, P. Monitoring of ubiquitin-proteasome activity in living cells using a Degron (dgn)-destabilized green fluorescent protein (GFP)-based reporter protein. *J. Vis. Exp. ***69**, 3327 (2012). 10.3791/3327 PMC3520582 23169445

[24] Feng, D. et al. Cbl-b enhances sensitivity to 5-fluorouracil via EGFR- and mitochondria-mediated pathways in gastric cancer cells. *Int. J. Mol. Sci.* **14**, 24399–24411 (2013). 10.3390/ijms141224399 PMC3876118 24351824

[25] Atamna, H., Brahmbhatt, M., Atamna, W., Shanower, G. A. & Dhahbi, J. M. ApoHRP-based assay to measure intracellular regulatory heme. *Metallomics* **7**, 309–321 (2015). 10.1039/c4mt00246f PMC4326600 25525887

[26] Poulos, T. L. Heme enzyme structure and function. *Chem. Rev.* **114**, 3919–3962 (2014). 10.1021/cr400415k PMC3981943 24400737

[27] Moran, A. E. et al. T cell receptor signal strength in T_reg_ and iNKT cell development demonstrated by a novel fluorescent reporter mouse. *J. Exp. Med.* **208**, 1279–1289 (2011). 10.1084/jem.20110308 PMC3173240 21606508

[28] Ma, X. et al. Cholesterol induces CD8^+^ T cell exhaustion in the tumor microenvironment. *Cell Metab.* **30**, 143–156 (2019). 10.1016/j.cmet.2019.04.002 PMC7061417 31031094

[29] Xu, S. et al. Uptake of oxidized lipids by the scavenger receptor CD36 promotes lipid peroxidation and dysfunction in CD8^+^ T cells in tumors. *Immunity* **54**, 1561–1577 (2021). 10.1016/j.immuni.2021.05.003 PMC9273026 34102100

[30] Zhang, K., Wang, M., Zhao, Y. & Wang, W. Taiji: system-level identification of key transcription factors reveals transcriptional waves in mouse embryonic development. *Sci. Adv.* **5**, eaav3262 (2019). 10.1126/sciadv.aav3262 PMC6436936 30944857

[31] Utzschneider, D. T. et al. T cells maintain an exhausted phenotype after antigen withdrawal and population reexpansion. *Nat. Immunol.* **14**, 603–610 (2013). 10.1038/ni.2606 23644506

[32] Watanabe-Matsui, M. et al. Heme regulates B-cell differentiation, antibody class switch, and heme oxygenase-1 expression in B cells as a ligand of Bach2. *Blood* **117**, 5438–5448 (2011). 10.1182/blood-2010-07-296483 21444915

[33] Shin, H. et al. A role for the transcriptional repressor Blimp-1 in CD8^+^ T cell exhaustion during chronic viral infection. *Immunity* **31**, 309–320 (2009). 10.1016/j.immuni.2009.06.019 PMC2747257 19664943

[34] Watanabe-Matsui, M. et al. Heme binds to an intrinsically disordered region of Bach2 and alters its conformation. *Arch. Biochem. Biophys.* **565**, 25–31 (2015). 10.1016/j.abb.2014.11.005 25444856

[35] Vardaka, P. et al. A cell-based bioluminescence assay reveals dose-dependent and contextual repression of AP-1-driven gene expression by BACH2. *Sci. Rep.* **10**, 18902 (2020). 10.1038/s41598-020-75732-z PMC7641119 33144667

[36] Huang, Q. et al. The primordial differentiation of tumor-specific memory CD8^+^ T cells as bona fide responders to PD-1/PD-L1 blockade in draining lymph nodes. *Cell* **185**, 4049–4066 (2022). 10.1016/j.cell.2022.09.020 36208623

[37] Andreatta, M. et al. Interpretation of T cell states from single-cell transcriptomics data using reference atlases. *Nat. Commun.* **12**, 2965 (2021). 10.1038/s41467-021-23324-4 PMC8137700 34017005

[38] Galmozzi, A. et al. PGRMC2 is an intracellular haem chaperone critical for adipocyte function. *Nature* **576**, 138–142 (2019). 10.1038/s41586-019-1774-2 PMC6895438 31748741

[39] Li, Z. et al. Functional diversification and dynamics of CAR-T cells in patients with B-ALL. *Cell Rep.* **42**, 113263 (2023). 10.1016/j.celrep.2023.113263 37851569

[40] Fraietta, J. A. et al. Determinants of response and resistance to CD19 chimeric antigen receptor (CAR) T cell therapy of chronic lymphocytic leukemia. *Nat. Med.* **24**, 563–571 (2018). 10.1038/s41591-018-0010-1 PMC6117613 29713085

[41] Bai, Z. et al. Single-cell antigen-specific landscape of CAR T infusion product identifies determinants of CD19-positive relapse in patients with ALL. *Sci. Adv.* **8**, eabj2820 (2022). 10.1126/sciadv.abj2820 PMC9177075 35675405

[42] Bai, Z. et al. Single-cell CAR T atlas reveals type 2 function in 8-year leukaemia remission. *Nature* **634**, 702–711 (2024). 10.1038/s41586-024-07762-w PMC11485231 39322664

[43] Richardson, P. G. et al. A phase 2 study of bortezomib in relapsed, refractory myeloma. *N. Engl. J. Med.* **348**, 2609–2617 (2003). 10.1056/NEJMoa030288 12826635

[44] Zhu, Z. et al. FOXP1 and KLF2 reciprocally regulate checkpoints of stem-like to effector transition in CAR T cells. *Nat. Immunol. ***25**, 117–128 (2024). 10.1038/s41590-023-01685-w PMC10841689 38012417

[45] Wagle, M. V. et al. Antigen-driven EGR2 expression is required for exhausted CD8^+^ T cell stability and maintenance. *Nat. Commun.* **12**, 2782 (2021). 10.1038/s41467-021-23044-9 PMC8119420 33986293

[46] Olesin, E., Nayar, R., Saikumar-Lakshmi, P. & Berg, L. J. The transcription factor Runx2 is required for long-term persistence of antiviral CD8^+^ memory T cells. *Immunohorizons* **2**, 251–261 (2018). 10.4049/immunohorizons.1800046 PMC6156005 30264035

[47] Kim, M. V., Ouyang, W., Liao, W., Zhang, M. Q. & Li, M. O. The transcription factor Foxo1 controls central-memory CD8^+^ T cell responses to infection. *Immunity* **39**, 286–297 (2013). 10.1016/j.immuni.2013.07.013 PMC3809840 23932570

[48] Zheng, Y. et al. KLF12 overcomes anti-PD-1 resistance by reducing galectin-1 in cancer cells. *J. Immunother. Cancer* **11**, e007286 (2023). 10.1136/jitc-2023-007286 PMC10432659 37586772

[49] Kelly, B. & Pearce, E. L. Amino assets: how amino acids support immunity. *Cell Metab* **32**, 154–175 (2020). 10.1016/j.cmet.2020.06.010 32649859

[50] Feng, B. et al. The type 2 cytokine Fc-IL-4 revitalizes exhausted CD8^+^ T cells against cancer. *Nature* **634**, 712–720 (2024). 10.1038/s41586-024-07962-4 PMC11485240 39322665

[51] Barton, P. R. et al. Super-killer CTLs are generated by single gene deletion of Bach2. *Eur. J. Immunol.* **52**, 1776–1788 (2022). 10.1002/eji.202249797 PMC9828676 36086884

[52] Buckman, J. F. et al. MitoTracker labeling in primary neuronal and astrocytic cultures: influence of mitochondrial membrane potential and oxidants. *J. Neurosci. Methods* **104**, 165–176 (2001). 10.1016/s0165-0270(00)00340-x 11164242

[53] Wegiel, B., Nemeth, Z., Correa-Costa, M., Bulmer, A. C. & Otterbein, L. E. Heme oxygenase-1: a metabolic nike. *Antioxid. Redox Signal.* **20**, 1709–1722 (2014). 10.1089/ars.2013.5667 PMC3961788 24180257

[54] Ma, X. et al. CD36-mediated ferroptosis dampens intratumoral CD8^+^ T cell effector function and impairs their antitumor ability. *Cell Metab.* **33**, 1001–1012 (2021). 10.1016/j.cmet.2021.02.015 PMC8102368 33691090

[55] Philip, M. et al. Heme exporter FLVCR is required for T cell development and peripheral survival. *J. Immunol.* **194**, 1677–1685 (2015). 10.4049/jimmunol.1402172 PMC4323866 25582857

[56] Kim, H. J., Khalimonchuk, O., Smith, P. M. & Winge, D. R. Structure, function, and assembly of heme centers in mitochondrial respiratory complexes. *Biochim. Biophys. Acta* **1823**, 1604–1616 (2012). 10.1016/j.bbamcr.2012.04.008 PMC3601904 22554985

[57] Scheiermann, C., Gibbs, J., Ince, L. & Loudon, A. Clocking in to immunity. *Nat. Rev. Immunol.* **18**, 423–437 (2018). 10.1038/s41577-018-0008-4 29662121

[58] Cervantes-Silva, M. P. et al. The circadian clock influences T cell responses to vaccination by regulating dendritic cell antigen processing. *Nat. Commun.* **13**, 7217 (2022). 10.1038/s41467-022-34897-z PMC9722918 36470865

[59] Wang, C. et al. Circadian tumor infiltration and function of CD8^+^ T cells dictate immunotherapy efficacy. *Cell* **187**, 2690–2702 (2024). 10.1016/j.cell.2024.04.015 38723627

[60] Shanker, A. et al. Bortezomib improves adoptive T-cell therapy by sensitizing cancer cells to FasL cytotoxicity. *Cancer Res.* **75**, 5260–5272 (2015). 10.1158/0008-5472.CAN-15-0794 PMC4681610 26494122

[61] Platt, R. J. et al. CRISPR-Cas9 knockin mice for genome editing and cancer modeling. *Cell* **159**, 440–455 (2014). 10.1016/j.cell.2014.09.014 PMC4265475 25263330

[62] McWilliams, T. G. et al. mito-QC illuminates mitophagy and mitochondrial architecture in vivo. *J. Cell Biol.* **214**, 333–345 (2016). 10.1083/jcb.201603039 PMC4970326 27458135

[63] Clark, N. C. et al. Conditional ablation of progesterone receptor membrane component 2 causes female premature reproductive senescence. *Endocrinology* **158**, 640–651 (2017). 10.1210/en.2016-1701 PMC5460782 28005395

[64] Meeth, K., Wang, J. X., Micevic, G., Damsky, W. & Bosenberg, M. W. The YUMM lines: a series of congenic mouse melanoma cell lines with defined genetic alterations. *Pigment Cell Melanoma Res.* **29**, 590–597 (2016). 10.1111/pcmr.12498 PMC5331933 27287723

[65] An, N. et al. Construction of a new anti-CD19 chimeric antigen receptor and the anti-leukemia function study of the transduced T cells. *Oncotarget* **7**, 10638–10649 (2016). 10.18632/oncotarget.7079 PMC4891147 26840021

[66] Trefny, M. P. et al. Deletion of SNX9 alleviates CD8 T cell exhaustion for effective cellular cancer immunotherapy. *Nat. Commun.* **14**, 86 (2023). 10.1038/s41467-022-35583-w PMC9895440 36732507

[67] Franco, F. et al. Regulatory circuits of mitophagy restrict distinct modes of cell death during memory CD8^+^ T cell formation. *Sci. Immunol.* **8**, eadf7579 (2023). 10.1126/sciimmunol.adf7579 37738363

[68] Doench, J. G. et al. Optimized sgRNA design to maximize activity and minimize off-target effects of CRISPR-Cas9. *Nat. Biotechnol.* **34**, 184–191 (2016). 10.1038/nbt.3437 PMC4744125 26780180

[69] Li, B., Ren, N., Yang, L., Liu, J. & Huang, Q. A qPCR method for genome editing efficiency determination and single-cell clone screening in human cells. *Sci. Rep.* **9**, 18877 (2019). 10.1038/s41598-019-55463-6 PMC6906436 31827197

[70] Di Conza, G. et al. Tumor-induced reshuffling of lipid composition on the endoplasmic reticulum membrane sustains macrophage survival and pro-tumorigenic activity. *Nat. Immunol.* **22**, 1403–1415 (2021). 10.1038/s41590-021-01047-4 PMC7611917 34686867

[71] Arguello, R. J. et al. SCENITH: a flow cytometry-based method to functionally profile energy metabolism with single-cell resolution. *Cell Metab.* **32**, 1063–1075 (2020). 10.1016/j.cmet.2020.11.007 PMC8407169 33264598

[72] Tsai, C. H. et al. Immunoediting instructs tumor metabolic reprogramming to support immune evasion. *Cell Metab.* **35**, 118–133 (2023). 10.1016/j.cmet.2022.12.003 PMC10375941 36599297

[73] Weirauch, M. T. et al. Determination and inference of eukaryotic transcription factor sequence specificity. *Cell* **158**, 1431–1443 (2014). 10.1016/j.cell.2014.08.009 PMC4163041 25215497

[74] Chen, E. Y. et al. Enrichr: interactive and collaborative HTML5 gene list enrichment analysis tool. *BMC Bioinform.* **14**, 128 (2013). 10.1186/1471-2105-14-128 PMC3637064 23586463

[75] Kuleshov, M. V. et al. Enrichr: a comprehensive gene set enrichment analysis web server 2016 update. *Nucleic Acids Res.* **44**, W90–W97 (2016). 10.1093/nar/gkw377 PMC4987924 27141961

[76] Xie, Z. et al. Gene set knowledge discovery with Enrichr. *Curr. Protoc.* **1**, e90 (2021). 10.1002/cpz1.90 PMC8152575 33780170

[77] Lun, A. T., Bach, K. & Marioni, J. C. Pooling across cells to normalize single-cell RNA sequencing data with many zero counts. *Genome Biol.* **17**, 75 (2016). 10.1186/s13059-016-0947-7 PMC4848819 27122128

[78] Hao, Y. et al. Dictionary learning for integrative, multimodal and scalable single-cell analysis. *Nat. Biotechnol.* **42**, 293–304 (2024). 10.1038/s41587-023-01767-y PMC10928517 37231261

[79] Massimo, A. & Santiago, C. ProjecTILs murine reference atlas of tumor-infiltrating T cells, version 1. *Figshare*10.6084/m9.figshare.12478571.v3 (2020).

[80] Love, M. I., Huber, W. & Anders, S. Moderated estimation of fold change and dispersion for RNA-seq data with DESeq2. *Genome Biol.* **15**, 550 (2014). 10.1186/s13059-014-0550-8 PMC4302049 25516281

[81] Zhu, A., Ibrahim, J. G. & Love, M. I. Heavy-tailed prior distributions for sequence count data: removing the noise and preserving large differences. *Bioinformatics* **35**, 2084–2092 (2019). 10.1093/bioinformatics/bty895 PMC6581436 30395178

[82] Wu, T. et al. clusterProfiler 4.0: a universal enrichment tool for interpreting omics data. *Innovation* **2**, 100141 (2021). 10.1016/j.xinn.2021.100141 PMC8454663 34557778

[83] Korsunsky, I. et al. Fast, sensitive and accurate integration of single-cell data with Harmony. *Nat. Methods* **16**, 1289–1296 (2019). 10.1038/s41592-019-0619-0 PMC6884693 31740819

[84] Dann, E., Henderson, N. C., Teichmann, S. A., Morgan, M. D. & Marioni, J. C. Differential abundance testing on single-cell data using *k*-nearest neighbor graphs. *Nat. Biotechnol.* **40**, 245–253 (2022). 10.1038/s41587-021-01033-z PMC7617075 34594043

[85] Zhang, K., Zemke, N. R., Armand, E. J. & Ren, B. A fast, scalable and versatile tool for analysis of single-cell omics data. *Nat. Methods* **21**, 217–227 (2024). 10.1038/s41592-023-02139-9 PMC10864184 38191932

