# Supplementary material for: Proteasome-guided haem signalling axis contributes to T cell exhaustion
Source: Nature. 2026 Mar 18;653(8114):548–57. doi: 10.1038/s41586-026-10250-y (PMC13658038; doi:10.1038/s41586-026-10250-y)

nature portfolio

https://doi.org/10.1038/s41586-026-10250-y

Supplementary information

Proteasome-guided haem signalling axis contributes to T cell exhaustion

In the format provided by the authors and unedited

Y. Xu et al., SI Figure1

a

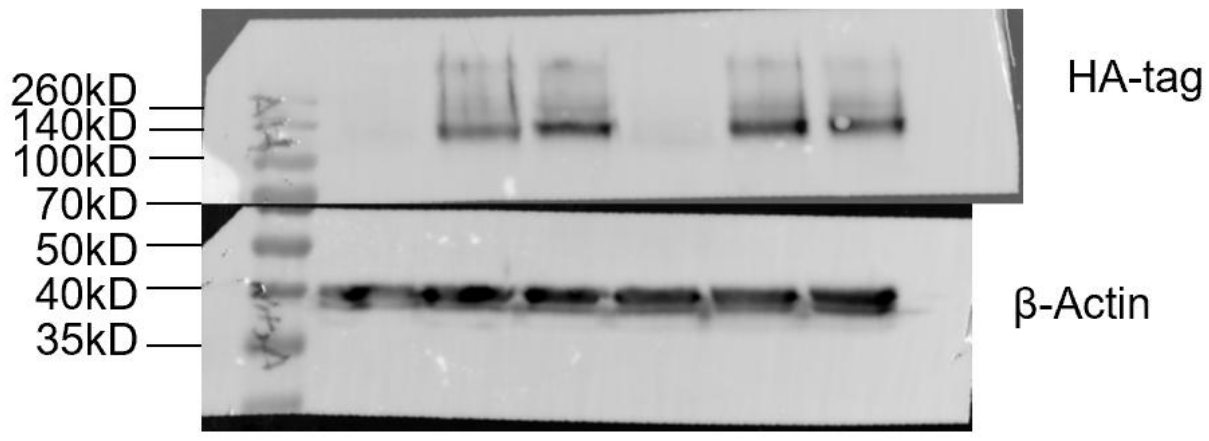

Supplement: Supplementary file 1 [file 41586_2026_10250_MOESM1_ESM.pdf]
