# Supplementary material for: Proteasome-guided haem signalling axis contributes to T cell exhaustion
Source: Nature. 2026 Mar 18;653(8114):548–57. doi: 10.1038/s41586-026-10250-y (PMC13658038; doi:10.1038/s41586-026-10250-y)
Supplement: Supplementary file 2 [file 41586_2026_10250_MOESM2_ESM.pdf]

nature portfolio

Corresponding author(s): Ping-Chih Ho

Last updated by author(s): Feb 6, 2026

Reporting Summary

Nature Portfolio wishes to improve the reproducibility of the work that we publish. This form provides structure for consistency and transparency in reporting. For further information on Nature Portfolio policies, see our Editorial Policies and the Editorial Policy Checklist.

Statistics

For all statistical analyses, confirm that the following items are present in the figure legend, table legend, main text, or Methods section.

n/a	Confirmed	
☐	☒	The exact sample size (*n*) for each experimental group/condition, given as a discrete number and unit of measurement
☐	☒	A statement on whether measurements were taken from distinct samples or whether the same sample was measured repeatedly
☐	☒	The statistical test(s) used AND whether they are one- or two-sided<br>*Only common tests should be described solely by name; describe more complex techniques in the Methods section.*
☐	☒	A description of all covariates tested
☐	☒	A description of any assumptions or corrections, such as tests of normality and adjustment for multiple comparisons
☐	☒	A full description of the statistical parameters including central tendency (e.g. means) or other basic estimates (e.g. regression coefficient) AND variation (e.g. standard deviation) or associated estimates of uncertainty (e.g. confidence intervals)
☐	☒	For null hypothesis testing, the test statistic (e.g. *F*, *t*, *r*) with confidence intervals, effect sizes, degrees of freedom and *P* value noted<br>*Give P values as exact values whenever suitable.*
☒	☐	For Bayesian analysis, information on the choice of priors and Markov chain Monte Carlo settings
☒	☐	For hierarchical and complex designs, identification of the appropriate level for tests and full reporting of outcomes
☒	☐	Estimates of effect sizes (e.g. Cohen's *d*, Pearson's *r*), indicating how they were calculated

Our web collection on statistics for biologists contains articles on many of the points above.

Software and code

Policy information about availability of computer code

Data collection

1. Flow cytometry data collection was performed with BD FACSDiva software (v.8.0.1)
2. Bulk RNA sequencing library was prepared by illumine Truseq Stranded RNA kit and sequenced by paired-end 150bp on Element Biosciences Aviti.
3. The gRNAs were designed using the publicly available online gRNA design tool CRISPick (https://portals.broadinstitute.org/gppx/crispick/public).
4. Single-cell RNA seq libraries were sequenced on the Illumina NovaSeq 6000 platform with NovaSeq 6000 S4 Reagent Kit (300 cycles).
5. For the protein degradation, proteomics were analyzed using an Easy Nano LC-Orbitrap Fusion System with a Nanospray Flex™ ion source (Thermo Fisher Scientific, USA).
6. For the protein ubiquitination, proteomics were analyzed using timsTOF HT or timsTOF_Pro2 mass spectrometer coupled to Thermo Scientific UltiMate 3000 system via a Nanospray Flex™ ion source (Bruker, Bremen, Germany).
7. Single-cell Multi-omics seq libraries were sequenced on the GeneMind SURFSeq 5000 platform.

Data analysis

1. Flow cytometry data were analyzed using FlowJo (version 10.8.1,Tree Star, Oregon, USA).
2. Statistical analysis was performed using GraphPad Prism (version 9.0.0, GraphPad software, Inc, La Jolla, CA, USA).
3. RAW bulk RNA-seq data used TrimGalore (v.0.6.6) for quality and adaptor trimming. The trimmed reads were mapped to the mm10 by using STAR (v.2.7.9a). The summarizeOverlaps function from the GenomicAlignments package (v.1.30.0) in R/Bioconductor (v.3.13) was applied for determining gene expression by counting the alignments to exons. The Gene Sets Enrichment Analysis (GSEA) was performed by GSEA software (v.4.3.2). The Principal Component Analysis (PCA) was performed by R (v.4.3.2).
4. RAW ATAC sequencing adapters were then removed and low-quality reads were filtered out using fastp software (v0.20.1). These reads were aligned to the mm10 mouse reference genome assembly using Bowtie2 (v2.4.1) set to the ‘’-very-sensitive’’ parameter. PCR duplicates were identified using samblaster (v0.1.24), and reads mapped to ENCODE black-listed regions were excluded prior to peak calling. Open

chromatin regions were identified through peak calling performed by MACS2 (v2.2.7.1), employing the options: -n-nomodel -keep -dup auto -g 2407883318 -p e-09 -extsize 147. Peaks coinciding with blacklisted regions were discarded. A consensus peak list was then created by merging ATAC-seq peaks from all samples using the BEDTools (v2.29.2) merge command. The identification of open chromatin regions facilitated PCA and differential accessibility was quantified using DESeq2. Taiji (v.1.2.0) with default parameters were used to integrate bulk RNA-seq and ATAC-seq data for key transcription factors identification. Cis-BP database (Build 2.00)was used for mouse motifs.
5. Mouse CD8+ T single cell RNA-seq: cell Ranger (V3.0.2, https://support.10xgenomics.com/) was used to process scRNA-seq data and generate the matrix data containing gene counts for each cell per sample. Downstream analyses, including data normalization, scaling and dimensional reduction were conducted employing Seurat v5 pipeline. For quality control filtering, cells with fewer than 900 and more than 6000 detected genes, fewer than 2400 or more than 38000 counts, lower than 0.13 or higher than 0.33 proportion of ribosomal genes, higher than 0.07 proportion of mitochondrial genes, lower than 0.8 log10 genes per UMI ratio, or higher than 0.03 proportion of Hemoglobulin-related genes were discarded. Cell type annotation was performed with ProjecTILs, using a murine reference atlas for tumor-infiltrating T cells subset only for CD8+ T subtypes. Differentially expressed genes for each cell type between conditions were identified using pseudobulk of each cell type per sample and employing negative binomial generalized linear model by DESeq2, considering the pairing the samples in the design. Fold change results were shrunk using the apeglm method. Significance was determined with the Wald test, with p-values adjusted using the Benjamini-Hochberg method to calculate the FDR. GSEA was performed on ranked genes based on -log10 of the adjusted p-value and the sign of the log2 fold change between conditions, utilizing clusterProfiler.
6. The raw data for human CAR-T scRNA-seq were mapped to the reference genome (GRCh38) using the Cell Ranger (V3.0.2, https://support.10xgenomics.com/), and then generate the matrix data containing gene counts for each cell per sample. Cells with fewer than 500 and more than 7000 detected genes were discarded and with a high fraction of mitochondrial genes (>20%) were removed. In addition, the proportion of ribosome related genes were controlled below 60%. The pre-processing data were normalized and scaled by Seurat (R package, version 4.3.0) workflow. The Harmony algorithm was used to remove batch effects between samples. PCA and UMAP were used for dimension reduction. The marker gene about different clusters were found by FindAllMarkers(). The trajectory analysis was used Monocle (version 2.30.0) based on its official documentation (https://cole-trapnell-lab.github.io/monocle-release/docs/). The different feature score were calculated by the AddModuleScore(). The standard miloR pipeline (https://github.com/MarioniLab/miloR) was applied to the converted object.
7. Proteomics raw data from DIA measurements were analyzed using the directDIA workflow in Spectronaut software (Biognosys, Switzerland). Identification was performed using the Uniprot mouse database with default settings. Digestion enzyme specificity was set to Trypsin/P. Modifications included carbamidomethylation of cysteine as a fixed modification, and oxidation of methionine and acetyl (protein N-terminus) as variable modifications. Up to two missed cleavages were allowed. FDR was estimated with the mProphet approach and set to 0.01 at both peptide precursor level and protein level. Cross-run normalization was done with mean peptide and precursor quantity. Differentially expressed proteins were identified by Wilcoxon t-test (cut off of log2 (fold change) > 0.75, p < 0.05) and used for gene enrichment analysis using Enrichr. To define protein degradation, all the quantified proteins were used to perform GO CC analysis and GO BP analysis using tidyproteomics package (v.1.7.2) in R. PCA was performed using R (v.4.3.2). To determine the difference among TN, TE, TM, TPEx and TTEx, differentially expressed proteins in each subset were compare against TN. The pathway enrichment analysis was performed in R by using tidyproteomics package (v1.8.5). For detecting the ubiquitination of proteins, differentially expressed proteins among TM, TE and TTEx subsets were identified relative to TE using a Wilcoxon test (log2FC > 1.5; p < 0.05). Differentially expressed proteins between MDR/MGlow and MDR/MGhigh cells were similarly identified using a Wilcoxon test (log2FC > 1.5; p < 0.05). Subcellular localization prediction was performed using WoLF PSORT (https://wolfpsort.hgc.jp/).
8. For the scMulti-omics seq, the raw data were processed using SeekArcTools (version 1.0.0). For scRNA-seq, barcodes and UMIs were extracted and corrected based on a whitelist, adapter sequences were trimmed, and reads were aligned to the GRCh38 reference genome using STAR, followed by annotation and UMI correction to generate gene count matrices. For scATAC-seq, barcodes were similarly processed, adapter contamination was removed, and reads were aligned with bwa mem. SnapATAC2 was then used to generate fragment files, call peaks with MACS3, compute QC metrics, and perform cell calling. The final output consisted of cell- and sample-level gene count matrices and peak count matrices, enabling subsequent multi-omic downstream analysis. For downstream analysis of scRNA-seq, quality control and preprocessing were performed using Seurat (R package, version 4.4.0). Cells with fewer than 500 or more than 6,000 detected genes, fewer than 500 or more than 30,000 RNA counts, or a mitochondrial gene fraction above 20% were removed. After normalization, scaling, and identification of variable features, dimensionality reduction was performed followed by batch correction using Harmony(version 1.2.3). UMAP was then applied for visualization. Differential gene expression was analyzed using FindAllMarkers() with a minimum expression threshold of 10% of cells and log2 fold change > 0.25. For downstream analysis of scATAC-seq, Seurat, Signac (version 1.11.0), and Harmony were used. Quality control included filtering based on duplicate removal, fragment counts, fragments per peak, fraction of reads mapping to blacklist regions, nucleosome signal, and transcriptional start site (TSS) enrichment. Cells with fewer than 500 or more than 100,000 ATAC reads were excluded, and peaks between 20 and 10,000 bp were retained. TF–IDF normalization and LSI were performed for dimensionality reduction, followed by batch correction using Harmony. Differentially accessible peaks were identified using FindAllMarkers() with a minimum expression threshold of 25% of cells while controlling for detected features as a latent variable. Peaks were visualized by IGV, and motif enrichment analysis was performed using motif data from the JASPAR 2022 database. The scMulti-Omic datasets were integrated using the Weighted Nearest Neighbor (WNN) method, followed by UMAP for dimensionality reduction and visualization. To define subclusters within the dataset, the expression levels of key genes were visualized using a dot plot. Feature scores for memory and cytotoxicity were calculated based on selected gene sets as described above. The interferon and interleukin feature scores were derived from gene sets in the KEGG pathways (https://www.genome.jp/kegg/). Cluster feature analysis was performed and visulated by clusterProfiler and using the GO gene sets.

For manuscripts utilizing custom algorithms or software that are central to the research but not yet described in published literature, software must be made available to editors and reviewers. We strongly encourage code deposition in a community repository (e.g. GitHub). See the Nature Portfolio guidelines for submitting code & software for further information.

Data

Policy information about availability of data

All manuscripts must include a data availability statement. This statement should provide the following information, where applicable:

- Accession codes, unique identifiers, or web links for publicly available datasets
- A description of any restrictions on data availability
- For clinical datasets or third party data, please ensure that the statement adheres to our policy

All data generated and/or analyzed in this study are publicly available. Bulk RNA-seq, ATAC-seq, and single-cell RNA-seq data from murine samples have been deposited in the NCBI Gene Expression Omnibus (GEO) database under accession numbers GSE312103, GSE312102, and GSE312100, respectively. Single-cell RNA-

seq data from human patient-derived CAR-T cells and single-cell multi-omics sequencing data from healthy human donor CAR-T cells have been deposited in the GSA-Human database at the China National Center for Bioinformation / Beijing Institute of Genomics, Chinese Academy of Sciences (https://ngdc.cncb.ac.cn/gsa-human), under accession numbers HRA012311 and HRA014168, respectively.

Research involving human participants, their data, or biological material

Policy information about studies with human participants or human data. See also policy information about sex, gender (identity/presentation), and sexual orientation and race, ethnicity and racism.

Reporting on sex and gender	Sex and/or gender was not considered in this study.
Reporting on race, ethnicity, or other socially relevant groupings	Race, ethnicity, or other socially relevant groupings were not considered in this study.
Population characteristics	Population characteristics were not considered as a variable in this study.
Recruitment	Patients diagnosed with B-cell acute lymphoblastic leukemia (B-ALL) were consecutively recruited at the Institute of Hematology and Blood Diseases Hospital, Chinese Academy of Medical Sciences and Peking Union Medical College. Eligible participants were identified by treating physicians during routine clinical care according to predefined inclusion criteria and were invited to participate in the study. All participants provided informed consent prior to enrollment. As this was a single-center study conducted at a tertiary referral hospital, selection bias related to referral patterns and disease severity may be present, which could limit the generalizability of the findings to broader B-ALL patient populations.
Ethics oversight	This study was reviewed and approved by the Review Committee of the Institute of Hematology and Blood Diseases Hospital, Chinese Academy of Medical Sciences (approval number: QT2016002-EC-2, QT2019006-EC-1 and IIT2016012-EC-1). All procedures involving human participants were conducted in accordance with institutional guidelines and the Declaration of Helsinki.

Note that full information on the approval of the study protocol must also be provided in the manuscript.

Field-specific reporting

Please select the one below that is the best fit for your research. If you are not sure, read the appropriate sections before making your selection.

☒ Life sciences ☐ Behavioural & social sciences ☐ Ecological, evolutionary & environmental sciences

For a reference copy of the document with all sections, see nature.com/documents/nr-reporting-summary-flat.pdf

Life sciences study design

All studies must disclose on these points even when the disclosure is negative.

Sample size	Group sizes for in vivo validation experiments were selected empirically based on previous results of the intra-group variation of tumor growth upon similar treatments. For in vitro experiments, group sizes were selected on the basis of previous publication and prior knowledge of variation and were replicated at least for 3 individual and independent experiments. Ref: Nat Immunol. 2020 Dec;21(12):1540-1551.
Data exclusions	Rout outlier tests were run with default parameters (Q = 1%) in Prism on all mouse experimental data due to inherent variability within the model system.
Replication	All presented results were repeatable. Replicates were used in all experiments as noted in figure legend or methods.
Randomization	Age and sex-matched animals were used for each experiment. Mice were randomized prior to treatment. For the experiments without mice, there is no randomization for samples because these in vitro experiments were observational and replicated at least for 3 individual, independent experiments.
Blinding	In vivo tumor engraftment and grouping was conducted randomly before treatment. Fully blinded experiments were not performed due to insufficient personnel availability to accommodate such situation and requirements for cage labeling and staffing needs.

Reporting for specific materials, systems and methods

We require information from authors about some types of materials, experimental systems and methods used in many studies. Here, indicate whether each material, system or method listed is relevant to your study. If you are not sure if a list item applies to your research, read the appropriate section before selecting a response.

Materials & experimental systems

n/a	Involved in the study	
☐	☒	Antibodies
☐	☒	Eukaryotic cell lines
☒	☐	Palaeontology and archaeology
☐	☒	Animals and other organisms
☒	☐	Clinical data
☒	☐	Dual use research of concern
☒	☐	Plants

Methods

n/a	Involved in the study	
☒	☐	ChIP-seq
☐	☒	Flow cytometry
☒	☐	MRI-based neuroimaging

Antibodies

Antibodies used

The following antibodies and staining reagents were purchased from BioLegend:
TruStain FcX™ PLUS (anti-mouse CD16/32) Antibody (Cat. # 156604, 1:200), Zombie Aqua™ Fixable Viability Kit (Cat. # 423102, 1:1000), anti-CD3e (Clone: 17A2, 1:100), anti-CD8a (Clone: 53.6.7, 1:100), anti-CD44 (Clone: IM7, 1:100), anti-CD45.1 (Clone: A20.1, 1:100), anti-CD45.2 (Clone: 104, 1:100), anti-PD1 (Clone: 29F.1A12, 1:100), anti-TIM3 (Clone: RMT3-23, 1:100), anti-Blimp1 (Clone: 5E7, 1:200), anti-CD62L (Clone: Mel-14, 1:100), anti-IFNγ (Clone: XMG1.2, 1:200), anti-TNFα (Clone: MP6-XT22, 1:200), anti-human CD8a (Clone: SK1, 1:100), anti- human PD-1 (Clone: EH12.2H7, 1:100), anti- human TIM3 (Clone: F38-2E2, 1:100), anti- human CD39 (Clone: A1, 1:100), Direct-Blot™ HRP anti-HA.11 Epitope Tag Antibody (Cat.# 901519, 1:1000) and anti-CD3ε (Clone: 145-2C11, 10ug/ml).

The following antibody were purchased from NOVUS:
anti-Bach2 (Clone: NBP2-34229, 1:200).

The following antibody were purchased from Cell Signaling:
Anti-PI3Kγ (Cell signaling Technology, Cat. # 4252, 1:200), anti-mTOR (Cell signaling Technology, Cat. # 2972, 1:200) and anti-TCF1/7 (Clone: C63D9, 1:400).

The following antibody was purchased from Merck: anti-puromycin (Clone: 12D10, 1:1000).

The following antibody was purchased from Sigma: anti-β actin (Cat.# A2228, 1:10000).

The following antibodies or staining reagents were purchased from Invitrogen: Foxp3/Transcription Factor Staining Buffer Set (Cat. # 00-5523-00).

Validation

For antibodies used in flow cytometry, each antibody has been validated by the manufacturer for use to detect mouse or human species targets. These antibodies are further validated and routinely used in our lab with good reproducibility. Detailed validation information for each antibody is available at the following websites:
TruStain FcX™ PLUS (anti-mouse CD16/32) Antibody (Cat. # 156604): https://www.biolegend.com/en-gb/explore-new-products/trustain-fcx-plus-anti-mouse-cd16-32-antibody-17085?GroupID=GROUP20
Zombie Aqua™ Fixable Viability Kit (Cat. # 423102): https://www.biolegend.com/en-gb/products/zombie-aqua-fixable-viability-kit-8444
anti-CD3e (Clone: 17A2): https://www.biolegend.com/en-gb/products/percp-cyanine5-5-anti-mouse-cd3-antibody-5596?GroupID=BLG242
anti-CD8a (Clone: 53.6.7): https://www.biolegend.com/en-gb/products/spark-plus-uv-395-anti-mouse-cd8a-antibody-25001
anti-CD44 (Clone: IM7): https://www.biolegend.com/en-gb/products/apc-cyanine7-anti-mouse-human-cd44-antibody-3933
anti-CD45.1 (Clone: A20.1): https://www.biolegend.com/en-gb/products/pe-cyanine7-anti-mouse-cd45-1-antibody-4917?GroupID=BLG1933
anti-CD45.2 (Clone: 104): https://www.biolegend.com/en-gb/products/brilliant-violet-421-anti-mouse-cd45-2-antibody-7328
anti-PD1 (Clone: 29F.1A12): https://www.biolegend.com/fr-ch/products/brilliant-violet-711-anti-mouse-cd279-pd-1-antibody-12303?GroupID=BLG7927
anti-TIM3 (Clone: RMT3-23): https://www.biolegend.com/en-gb/products/brilliant-violet-605-anti-mouse-cd366-tim-3-antibody-13391?GroupID=BLG10787
anti-Blimp1 (Clone: 5E7): https://www.biolegend.com/en-gb/products/pe-anti-mouse-blimp-1-antibody-12328?GroupID=BLG14433
anti-CD62L (Clone: Mel-14): https://www.biolegend.com/en-gb/products/fitc-anti-mouse-cd62l-antibody-384?GroupID=BLG10714
anti-IFNγ (Clone: XMG1.2): https://www.biolegend.com/en-gb/products/pe-anti-mouse-ifn-gamma-antibody-997?GroupID=GROUP24
anti-TNFα (Clone: MP6-XT22): https://www.biolegend.com/en-gb/products/brilliant-violet-711-anti-mouse-tnf-alpha-antibody-13622?GroupID=GROUP24
anti-human CD8a (Clone: SK1): https://www.biolegend.com/en-gb/products/brilliant-violet-421-anti-human-cd8-antibody-13512?GroupID=BLG10167
anti- human PD-1 (Clone: EH12.2H7): https://www.biolegend.com/en-gb/products/apc-cyanine7-anti-human-cd279-pd-1-antibody-7121?GroupID=BLG5466
anti- human TIM3 (Clone: F38-2E2): https://www.biolegend.com/en-gb/products/pe-anti-human-cd366-tim-3-antibody-6121?GroupID=BLG9937
anti- human CD39 (Clone: A1): https://www.biolegend.com/en-us/cytokines-chemokines-and-factors/apc-anti-human-cd39-antibody-6275?GroupID=BLG5462
Direct-Blot™ HRP anti-HA.11 Epitope Tag Antibody (Cat.# 901519): https://www.biolegend.com/en-gb/products/direct-blot-hrp-anti-ha11-epitope-tag-antibody-14398?GroupID=GROUP26.

anti-CD3ε (Clone: 145-2C11): https://www.biolegend.com/en-gb/products/ultra-leaf-purified-anti-mouse-cd3epsilon-antibody-7722?GroupID=BLG6744

The following antibody were purchased from NOVUS:
anti-Bach2 (Clone: NBP2-34229): https://www.novusbio.com/products/bach2-antibody_nbp2-34229

The following antibody were purchased from Cell Signaling:
Anti-PI3Kγ (Cell signaling Technology, Cat. # 4252): https://www.cellsignal.com/products/primary-antibodies/pi3-kinase-p110g-antibody/4252
anti-mTOR (Cell signaling Technology, Cat. # 2972): https://www.cellsignal.com/products/primary-antibodies/mtor-antibody/2972
anti-TCF1/7 (Clone: C63D9): https://www.cellsignal.cn/products/primary-antibodies/tcf1-tcf7-c63d9-rabbit-mab/2203

The following antibody was purchased from Merck:
anti-puromycin (Clone: 12D10): https://www.merckmillipore.com/CN/zh/product/Anti-Puromycin-clone-12D10-Alexa-Fluor-647-Conjugate-Antibody,MM_NF-MABE343-AF647

The following antibody was purchased from Sigma:
anti-β actin (Cat.# A2228): https://www.sigmaaldrich.cn/CN/zh/product/sigma/a2228
msockid=1ae55548e63a6d5b379c4088e7f36c91

Eukaryotic cell lines

Policy information about cell lines and Sex and Gender in Research

Cell line source(s)	The YUMM1.7 melanoma cell line was provided by M. Bosenberg (Yale University, USA). The YUMM1.7-gp33 cell line was created by introducing lentivirus carrying gp33 peptide expression cassettes into the parental cells (Nat Immunol. 2020 Dec;21(12):1540-1551). The YUMM1.7-HER2 cell line was created by introducing lentivirus carrying human HER2 expression cassettes into the parental cells. Nalm6-luc2 cells were provided by J. Wang (Institute of Hematology and Blood Diseases Hospital, Chinese Academy of Medical Sciences and Peking Union Medical College).
Authentication	None of the cell lines were authenticated in these studies. In all related studies, cell lines with low passage number were used.
Mycoplasma contamination	All cell lines were confirmed mycoplasma negative.
Commonly misidentified lines (See ICLAC register)	No commonly misidentified cell lines were used.

Animals and other research organisms

Policy information about studies involving animals; ARRIVE guidelines recommended for reporting animal research, and Sex and Gender in Research

Laboratory animals	C57BL/6J, B6 CD45.1 (B6.SJL-Ptprca Pepcb/BoyJ), Nur77GFP (C57BL/6-Tg(Nr4a1-EGFP/cre)820Khog/J), Rag1-/- (B6.129S7-Rag1tm1Mom/J), Cas9fl/fl (Rosa26-LSL-Cas9 knockin) mice were purchased from the Jackson Laboratory. P14+ CD4Cre were gifts from Prof. D. Zehn (Technical University of Munich, Germany). P14+CD4Cre+Cas9+ were bred by P14+ CD4Cre and Rosa26LSL-Cas9 mice. Mito-QC mice were obtained from I.G. Ganley, Pgrmc2fl/fl mice from Prof. E. Saez (The Scripps Institute, USA), and P14+ Tcf1-eGFP reporter mice from Prof. W. Held (University of Lausanne, Switzerland), B6J Cas9 mice from Prof. J. Joyce (University of Lausanne, Switzerland) Bach2fl/fl mice from Prof. M. Kurosakiand (RIKEN Center for Integrative Medical Sciences, Japan) and CD4Cre-Bmal1fl/fl mice 64 from Prof. C. Scheiermann. P14+ CD4Cre Bach2fl/fl mice were bred by P14+ CD4Cre and Bach2fl/fl. P14+ CD4Cre Pgrmc2fl/fl mice were bred by P14+ CD4Cre and Pgrmc2fl/fl. C-NKG (NOD.Cg-PrkdcscidIl2rgem1cya/Cya) mice were purchased from Cyagen Biotechnology Co., Ltd., China. The mice were housed in the animal facility of the University of Lausanne or Institute of Hematology and Blood Diseases Hospital and were kept in individually ventilated cages, at 19-23 °C, with 45-65% humidity, and with a 12 h dark/light cycle. All mice used were at 5-12 weeks of age.
Wild animals	Study did not involve wild animals.
Reporting on sex	Sex is not relevant in this study, and most animals used were female mice.
Field-collected samples	Study did not involve field-collected samples.
Ethics oversight	Experimental procedures in mouse studies were approved by the Swiss authorities (Canton of Vaud, animal protocol IDs VD3309, VD3250, VD3528, VD3765 and VD3710) or Institutional Animal Care and Use Committee of Peking Union Medical College (KT2020061-EC-1) and performed in accordance with the guidelines from the animal facility of the University of Lausanne or Institute of Hematology and Blood Diseases Hospital. Mice were euthanized when body weight loss was beyond 15%, or the tumor area reached 1000 mm3 (as a predetermined endpoint), or other ending points reach the requirements of the animal licenses.

Note that full information on the approval of the study protocol must also be provided in the manuscript.

Plants

Seed stocks	Plants or seed stocks were not used in this study.
Novel plant genotypes	Plant genotypes are not relevant in this study
Authentication	Plant authentication is not performed in this study

Flow Cytometry

Plots

Confirm that:

☒ The axis labels state the marker and fluorochrome used (e.g. CD4-FITC).

☒ The axis scales are clearly visible. Include numbers along axes only for bottom left plot of group (a 'group' is an analysis of identical markers).

☒ All plots are contour plots with outliers or pseudocolor plots.

☒ A numerical value for number of cells or percentage (with statistics) is provided.

Methodology

Sample preparation	Tumor was minced in RPMI medium with 2% FBS, 1% P/S, 1 µg/ml DNaseI (Sigma-Aldrich, Cat. # D5025) and 1 mg/ml collagenase (Sigma-Aldrich, Cat. # C5138), followed by digestion at 37 °C for 1 hour. After digestion, the sample was filtered through a 70-µm cell strainer (FisherScientific, Cat. # 11597522). Then tumor infiltrated lymphocytes (TILs) were enrichment by density gradient centrifugation (800g, 30 min) at 25 °C with 40% and 80% Percoll (MgBCH-Milain, Cat. # 41-17-0891-01). Then the middle layer was collected for further analysis. Similarly, spleens and lymph nodes were collected and minced through strainers (70 µm) and then RBC inside were lysed with ACK lysing buffer for 2 min at room temperature before antibody staining and flow cytometry analysis. Blood samples were collected and 1:1 diluted in the PBS buffer and PBMC were isolated by Lymphoprep (AxonLab AG, Cat. # 12053231).
Instrument	Flow analysis was performed by BD LSRFortessa flow cytometer with BD FACSDiva software (v.8.0.1). Cell sorting was performed with FACS Aria II (BD Biosciences) or SH800S Cell Sorter (SONY).
Software	Flow cytometry data were analyzed using FlowJo 10.6.1 (Tree Star, Oregon, USA).
Cell population abundance	For a typical analysis, >10k leukocytes were collected for further gating.
Gating strategy	We used standard gating strategies: gating on the typical lymphocyte population based on FSC-SSC signals, doublet exclusion based on FSC-H and FSC-A comparison, Live / Dead discrimination based on fixable Aqua dye signals. Murine T cell populations were identified based on the expression markers listed below. CD8 T cells: CD44+/CD3+/CD8+; Progenitor exhausted CD8+ T cells: TCF1+PD1+TIM3-; intermediate exhausted CD8+ T cells: TCF1-PD1+TIM3- and the terminal exhausted T cells: TCF1-PD1+TIM3+. Human T cell populations were identified based on the expression markers listed below. CD8 T cells: CD3+/CD8+; Progenitor exhausted CD8+ T cells: PD1lowTIM3-CD39- and the terminal exhausted T cells: PD1 +TIM3+CD39+.

☒ Tick this box to confirm that a figure exemplifying the gating strategy is provided in the Supplementary Information.
