# Supplementary material for: Proteasome-guided haem signalling axis contributes to T cell exhaustion
Source: Nature. 2026 Mar 18;653(8114):548–57. doi: 10.1038/s41586-026-10250-y (PMC13658038; doi:10.1038/s41586-026-10250-y)
Supplement: Supplementary file 3 [file 41586_2026_10250_MOESM4_ESM.pdf]

nature portfolio

Peer Review File

Proteasome-guided heme signaling axis contributes to T cell exhaustion

Corresponding Author: Professor Ping-Chih Ho

Any redactions in this file are there to maintain patient confidentiality, the confidentiality of unpublished data, or to remove third-party material.

This file contains all reviewer reports in order by version, followed by all author rebuttals in order by version.

Version 0:

Reviewer comments:

Referee #1

(Remarks to the Author)
In their manuscript, Xu et al. report that the previously known accumulation of depolarized mitochondria in exhausted t cells leads to heme accumulation after degradation of mitochondrial proteins and that heme binds to Bach2, leading to increased expression of Blimp1, which further promotes t cell exhaustion. Additionally, the authors found that several circadian clock molecules are heme proteins and therefore also involved in T cell exhaustion. This study provides another layer of mechanistic insight within the previously reported spectrum of mechanisms by which mitochondrial depolarization engaged transcriptional networks of t cell exhaustion. It links the metabolic phenomenon of accumulation of depolarized mitochondria to heme, which is linked to Blimp1, thus proposing a new target for immunotherapies in cancer.

Major issues:

The role of heme for T cell exhaustion in the situation of CAR T cell therapies has previously been published (Gupta et al. Blood 2023). It would be helpful if the authors could delineate their findings from previously published reports in their manuscript, given close similarities as well as discrepant findings. It would also be helpful to cite this study.

The annotation of different exhausted t cell subsets for subsequent correlation with proteasome-related genes, follows a biased approach. It would be helpful to also include controls, such as naïve T cells from the blood, on the negative side of the spectrum of exhaustion. It seems that the evidence for the conclusion that exhausted t cells have increased proteasome activity comes only from the public scRNAseq data. Overall, the correlation of exhaustion with actual proteasome activity requires experimental validation.

The authors start out with human CAR-T cell data, highlighting the association of the proteasome with exhaustion using single-cell transcriptomic reanalyses but then switch to mouse cells for the next mechanistic steps. It would strengthen the story if the proposed heme-bases mechanism of T cell exhaustion could also be tested in human T cells given that they even had access to clinical samples from patients with CAR T cell therapy. But also basic tests with ex vivo isolated and cultured (i.e. repetitive restimulations) human CD8 T cells would be helpful to evaluate the relevance of the findings for immunotherapies, as proposed by the authors.

The title of the manuscript highlights heme as a metabolic checkpoint. The manuscript, in its current form, is quite vague with respect to mechanistically testing the role of heme in T cell metabolism (so far primarily omics and bioinformatics). It would be helpful to see how it directly affects selected major metabolic pathways that have previously been associated to T cell exhaustion/stemness with experimental evidence.

In Fig.2 it would be helpful to not only sort the TILs according to their mitochondrial phenotypes but also according to a cellular classification of exhaustion vs. stemness to directly make the association of proteosomal degradation with t cell exhaustion (not only indirectly via mitochondrial states). Data that follows in fig. 3 would be helpful to support Fig. 2.

The data that heme regulates circadian rhythm molecules is exciting. However, these data are quite disconnected from the logical flow of the story and seem to be a stand-alone topic in the current state of the paper. It would be helpful to elaborate more experimentally on the role of the proposed molecules for exhaustion, metabolism and anti-tumor immunity.

Minor issues.

The manuscript requires English editing. It is in several instances difficult to understand what the authors mean due to grammar and spelling issues.

The figure legends and methods lack details required to fully understand the experimental conditions and bioinformatic analysis. Figures contain colors (probably for highlighting certain aspects) without indicating their purpose (e.g. Fig.2b-d).

Some figures contain statistical information, others not (e.g. violin plots of scRNAseq data). It would be helpful if p values can consistently be reported in the respective figures.

Referee #2

(Remarks to the Author)
In the manuscript entitled “Heme-mediated signaling acts as a metabolic checkpoint to orchestrate T cell exhaustion”, Xu et al. show that terminally exhausted CD8 T cells express an enhanced proteasome gene signature compared to other Tex and non-Tex populations. Xu et al. reason that the increased proteasome gene signature is due to the accumulation of depolarized mitochondria in terminally exhausted CD8 T cells. The authors demonstrate that exhausted/CD8 T cells with depolarized mitochondria degrade more mitochondrial proteins leading to greater intracellular regulatory heme concentrations which they claim leads to impacts on different transcription factors that ultimately control T cell exhaustion.

The work is interesting but due to several major concerns I recommend this manuscript be rejected. The manuscript presents many different pathways that impact exhaustion by themselves but are loosely linked through intracellular heme release. The presentation of these pathways are not logically linked and there is some contradictory data about hemin treatment and Bach2. These issues, combined with some typos and odd grammatical errors, makes the manuscript difficult to follow. Even if these issues are addressed in a revision, this manuscript does not meet the level of novelty expected of Nature. In my opinion this work is more suitable for a more specialized immunology journal after substantial revision/restructuring.

Major concerns:

1. The major scientific issue with the manuscript is that the authors do not convincingly show that heme drives exhaustion (the main conclusion of the paper). In Figure 3 the authors demonstrate that hemin, in vitro, is sufficient to reduce cytokine production but also reduces proliferation (Supp Fig 3e), making it difficult to interpret if the lower cytokine production is due to lower proliferation/TCR stimulation (hemin potentially decreases CD44 but this is not shown) or increased cell death (viability is not shown). Further, while decreased functionality is the true readout of T cell exhaustion, the authors don’t show any other exhaustion associated markers by flow cytometry in their in vitro experiments (Fig 3a-f). Their in vivo transfer experiments (Fig 3i-m) only show modest affects, with the largest being cytokine production and cell number/expansion. These data could also be interpreted as hemin decreasing the proliferation and cytokine production of T cells independent of affecting exhaustion.

2. In figure 3n, the authors demonstrate that hemin treatment, which increases intracellular heme, increases Bach2 transcriptional activity and decreases Blimp1 transcriptional activity. However, in figure 4b-c they show that hemin treatment lowers Bach2 expression and increases Blimp1 expression. While expression does not necessarily equal activity, these contradictory results are not explained and confound the conclusions of hemin treatment on T cell function/exhaustion.

3. A lot of genomics data was used to characterize different Tex populations that arise from the Bach2 heme binding mutant. However, there was no functional data (cytokine production, tumor growth) that demonstrated any biological significance of the increased Tpex population that the Bach2 mutant T cells brought about. In line with this the authors even admit that “… overexpressing a heme-binding insensitive Bach2 mutant in these T cells failed to achieve these beneficial outcomes (Figure 4d, 4h and Extended Data Figure 4o)”. These data and this statement make it clear that heme binding by Bach2 is not a driving mechanism for how heme impacts T cell function/exhaustion. These data make it unclear why Bach2 was pursued as a mechanism in the first place.

Referee #3

(Remarks to the Author)
In this mansucript, Xu and colleagues identify a proteasome gene signature in terminally exhausted TIL T cells, and show that pre-treatment of CAR T cells with the proteasome inhibitor bortezomib leads to an increase in anti-tumoral effect. This is mechanistically explained by the release of heme from mitochondrial proteins degraded via the proteasome, leading to

harmful effects on T cell function. Further, they show that this is mediated via by binding of heme to Bach2, and that knockout of the nuclear heme importer Pgrmc3 improves anti-tumoral response. Finally, they identify the circadian clock gene Bmal1 as a heme target, and show that its deletion in T cells also improves T cell functionality.

The importance of the proteasome in CD8+ T cell function has been shown previously (e.g PMID 28846070, PMID 21497118 - the former should be cited as a minimum), but not in the context of anti-tumoral immunity, and the translational potential of this work is therefore very high.

However, to fully substantiate this and the proposed underlying mechanisms, addition clarification is needed.

Generally, one of the issues of the manuscript is the extensive use of normalisation (undermining the understanding of interexperimental variation), and paired t tests on non-normally distributed data, or non-paired data.

Related to Figure 1.

A-B. What is the basis of the proteosome score? Is this from an existing database e.g. GO/Biocarta, or was it generated by the authors? Is there overlap with this list and other cluster defining genes? The gene list needs to be given.
E. What time point are these single cell data from? Extended Data 1D mentions day 9 or day 21.
G. The proportion analysis needs to be done in a more statistically rigorous way, for example using scCODA, Milo, or other computational technique.
K. This data can't be used to rule out changes in cell proliferation because there is no info on cell viability and maintenance. Proliferation data e.g. CFSE dilution, and the effect on viability needs to be shown.
M. The effect size here is very small and the difference is non-statistically significant.
N. There needs to be a statistical test comparing the CAR-T +/ bortezomib groups.
O. The duration of bortezomib treatment has not been specified in the legend or methods. Many other critical details like injection regimen, CAR construct, or cell numbers have not been included in the legends. The effect of the CAR-T treatment is minimal compared to the control VEC-T condition. Is that expected?

Related to Figure 2.

A. The toxicity and non-specific effect of Mitotracker dyes on mitochondrial function and dynamics have neither been critically tested nor acknowledged as a confounder or study limitation. Particularly with MTDR FM such effects have been reported in the field (PMID 11164242).

C-D. It is unclear why these proteomics experiments were performed in the presence of CHX, textual clarification and justification are needed. Using CHX as a main condition in the comparisons might introduce mitochondrial bias if cells have elevated rates of active mitochondrial translation. Without the cytoplasmic protein input into the mitochondrial proteome, an unfolded protein response in the mitochondria may be activated and the magnitude of this activation might differ between cell types tested, introducing bias. So, the authors should compare mitochondrial translation rates between depolarised vs healthy cell populations. Ideally, they should also repeat the proteomics experiments with a condition containing mitochondrial translation inhibitor like chloramphenicol, or using a SILAC based methodology.

F-G. The legend indicates that the authors subjected cells to a combination of oligmycin A, antimycin A, Mdivi-1 and CHX treatment. Whereas, on the actual figure only CHX is mentioned and in the text describing these findings, none of these inhibitors were mentioned or acknowledged. Why were they used? This is an extremely potent cocktail and may have a major effect on the outcome of the experiment. Also, the split scale rather exaggerates the effect size, which is modest.

H-I. Paired t tests are inappropriate here as the data are not paired (it would seem, assuming that each subset was separately repetitively stimulated). Further, the data has been normalised so it is no longer normally distributed. Were inhibitors (e.g. oligomycin etc) used in I?

K. What is the P value signifying? Regardless, the biggest issue is that the decrease in relative RH amount following MG132 treatment occurs at comparable rates between cell types, contradicting earlier findings suggesting MGlow cells have higher rates of proteasomal liberation of RH from mitochondria.

Related to Figure 3.

Extended Data Fig. 3B - paired t test used inappropriately

The statement one line 227 that 'these findings indicate that the degradation of mitochondrial proteins increase RH levels, which correlates with the severity of T cell exhaustion' has not been substantiated.

Related to Figure 4.

C. This experiment needs to be performed on Bach2 mutant cells to see if the increase in Blimp1 is prevented.

Extended Data Fig. 4A-D. Inhibitors were used for unjustified reasons.

Related to Figure 5.

Extended Data Fig. 5c-j. Again the statistical test used here is appropriate as these data are neither paired nor normally distributed (e and g).

Related to Figure 6.

The use of paired t tests is not appropriate as the data are not paired, and sometimes also not normally distributed. Why are points joined?

Version 1:

Reviewer comments:

Referee #3

(Remarks to the Author)

In this revised version of the manuscript, there have been significant improvements in terms of clarity and most of my concerns have been addressed, but as before, the use of statistics is not ideal, with an over-reliance on paired t tests which can lead to an artificially low standard error and false positives. Therefore, reanalysis of many experiments is required, as detailed below. This will probably not change the majority of the findings of the work however, which remain of interest.

Please see my responses to the rebuttal below.

Referee #3 (Remarks to the Author):
In this manuscript, Xu and colleagues identify a proteasome gene signature in terminally exhausted TIL T cells, and show that pre-treatment of CAR T cells with the proteasome inhibitor bortezomib leads to an increase in anti-tumoral effect. This is mechanistically explained by the release of heme from mitochondrial proteins degraded via the proteasome, leading to harmful effects on T cell function. Further, they show that this is mediated via by binding of heme to Bach2, and that knockout of the nuclear heme importer Pgrmc2 improves anti-tumoral response. Finally, they identify the circadian clock gene Bmal1 as a heme target, and show that its deletion in T cells also improves T cell functionality.

The importance of the proteasome in CD8+ T cell function has been shown previously (e.g PMID 28846070, PMID 21497118 - the former should be cited as a minimum), but not in the context of anti-tumoral immunity, and the translational potential of this work is therefore very high.
Reponses: We thank the reviewer for the valuable suggestion and extremely positive feedback about out study. We apologize for missing the citations during the editing process. We have now discussed the role of the proteasome in CD8+ T cell function in the discussion section and have cited the suggested study (PMID 28846070) as recommended in the revised manuscript.

However, to fully substantiate this and the proposed underlying mechanisms, addition clarification is needed.

Generally, one of the issues of the manuscript is the extensive use of normalization (undermining the understanding of interexperimental variation), and paired t tests on non-normally distributed data, or non-paired data.
Reponses: We thank the reviewer for highlighting these important points. Regarding the extensive use of normalization, we acknowledge the concern about potentially undermining the understanding of inter-experimental variation. However, we would like to explain that several results presented are cumulative data from several independent experiments. For certain assays, such as the heme assay and FACS analyses of Bach2 and Blimp1 expression, the variation between independent experiments was too large to combine the results without normalization. To address this, we have provided additional explanations in the Methods section to clarify the rationale for normalization and its application to ensure comparability across experiments. Furthermore, we now present unnormalized data for key experiments in the revised manuscript to enhance transparency regarding inter-experimental variability. As for the use of paired t-tests on non-normally distributed or non-paired data, we have thoroughly re-evaluated our statistical analyses to ensure the use of appropriate tests. For data that are not normally distributed, we have applied non-parametric tests (e.g., Mann-Whitney U test or Wilcoxon signed-rank test). Similarly, for non-paired data, we used unpaired t-tests or equivalent non-parametric tests where appropriate. These revisions have been detailed in the revised Methods section, and the figure legends have been updated accordingly. We hope these changes address the reviewer's concerns and enhance the rigor and transparency of our analyses.

Whilst I appreciate the authors taking this point on, there are still many instances of incorrect statistical testing which have not been corrected. For example, in Fig 1k, 2b, Extended data fig 4. ANOVA and multiple t tests are used variably. There is no description of how multiple testing is corrected in either the figure legends or methods section. In most cases these issues will not affect the overall validity of the work, but may do in some cases.

Related to Figure 1.

A-B. What is the basis of the proteosome score? Is this from an existing database e.g. GO/Biocarta, or was it generated by

the authors? Is there overlap with this list and other cluster defining genes? The gene list needs to be given.
Reponses: We appreciate the reviewer's question regarding the basis of the proteasome score. The proteasome score was calculated using genes annotated in the KEGG proteasome pathway (hsa03050) based on the gene weighting approach previously published by our collaborator 9. This list is publicly available and has been curated as part of the KEGG database. For clarity and reproducibility, we have mentioned it in the Material and Method section and have included the full gene list used for the proteasome score in the Extended Table 9 of the revised manuscript.

Thank you

E. What time point are these single cell data from? Extended Data 1D mentions day 9 or day 21.
Reponses: We thank the reviewer for raising this question. The single-cell data were obtained from CAR-T cells sorted from the peripheral blood of patients with B cell acute lymphoblastic leukemia at two time points: Day 9 (Peak) and Day 21 (Late) post anti-CD19 CAR-T infusion. To avoid potential bias and cover all cells collected from this cohort, all sorted CAR-T cells from these time points were used for scRNA-seq analysis. To ensure clarity, we have included this information in the figure legend for the revised Figure 6c (previously Figure 1e).

Thank you

G. The proportion analysis needs to be done in a more statistically rigorous way, for example using scCODA, Milo, or other computational technique.
Reponses: We appreciate the reviewer's suggestion to enhance the statistical rigor of our proportion analysis. Following this recommendation, we utilized Milo for the statistical analysis. The updated results are now presented in the revised Figure 6e in the revised manuscript.

Thank you. This analysis should also be performed on the single cell data in Fig 3j.

K. This data can't be used to rule out changes in cell proliferation because there is no info on cell viability and maintenance. Proliferation data e.g. CFSE dilution, and the effect on viability needs to be shown.
Reponses: Thank you for this helpful suggestion. In response to this concern, we re-performed the whole human CD19 CAR T-cell experiment and incorporated additional experiments to assess both T cell proliferation and viability after 0.1 nM Bortezomib treatment. Specifically, we used CellTrace Far Red dilution to evaluate proliferation and Zombie Aqua staining to determine viability of T cells at day 5 post CAR transduction. Our results showed that Bortezomib treatment does not impact proliferation and viability of CD19 CAR T-cells compared to those treated with the control vehicle (revised Figure 6i–j). We have included these findings in the revised manuscript to address the reviewer's concern.

Thank you. In new Fig 1k-l, why are the same type of data presented differently? Why are paired (incorrect) and unpaired t tests used for these similar panels?

M. The effect size here is very small and the difference is non-statistically significant.
Reponses: Thank you for this insightful observation. To address this concern, we re-performed the experiments and refined our approach by treating CAR-T cells with a lower dose of Bortezomib (0.1 nM) for only 24 hours on day 5 post-CAR transduction, rather than at the initiation of T cell activation. This adjustment minimizes potential effects of Bortezomib during the priming phase. With this refined experimental design, we focused on the expression of exhaustion markers, including PD-1, TIM-3 and CD39, in CD19 CAR T-cells following in vitro repetitive stimulation with target cells, as well as their in vivo anti-tumor responses (revised Figure 6k-q). Our new results continue to support our conclusion that Bortezomib pre-treatment during CAR T-cell production enhances resistance to exhaustion under repetitive stimulation and provides stronger therapeutic benefits.

Thank you, although it remains the case that the effect size is small.

N. There needs to be a statistical test comparing the CAR-T +/ bortezomib groups.
Reponses: Thank you for the suggestion to include a direct statistical comparison between the CAR-T ± bortezomib groups. In the revised manuscript, we have performed and reported statistical analyses accordingly. Specifically, we pre-treated anti-CD19 CAR-T cells with a low dose of bortezomib (0.1 ng/ml) for 24 hours on Day 5 post-CAR transduction, followed by transfer of 1×106 CAR-T cells into mice 4 days post tumor engraftment (5×105 Nalm6 cells). The body weight and survival of each group are provided with statistical analyses in the revised Figure 6p-q. For this longitudinal body weight changes, we employed a repeated-measures two-way ANOVA with corrections for multiple comparisons. These analyses indicated that bortezomib pre-treatment significantly enhanced CAR-T cell anti-tumor activity compared to CAR-T alone.

Thank you.

O. The duration of bortezomib treatment has not been specified in the legend or methods. Many other critical details like injection regimen, CAR construct, or cell numbers have not been included in the legends. The effect of the CAR-T treatment is minimal compared to the control VEC-T condition. Is that expected?
Reponses: We appreciate the reviewer's comments and apologize for any confusion regarding our experimental details. In the revised manuscript, we have included the complete injection regimen and bortezomib treatment duration in the legend to revised Figure 6o, which clarifies the timing of each intervention. The construction of our anti-CD19 CAR-T is a second-generation design with a 4-1BB intracellular co-stimulatory domain, and its scFv recognition region is derived from our previously characterized HIB19 antibody clone 10,11. We have also specified the CAR-T cell numbers and other procedural details in the revised figure legend to ensure full transparency. Regarding the concern about the beneficial effects of CAR-T cells, our new analyses demonstrated that CAR-T treatment significantly prolongs the survival of leukemia-bearing mice compared to the control condition (revised Figure 6p-q).

Thanks.

Related to Figure 2.

A. The toxicity and non-specific effect of Mitotracker dyes on mitochondrial function and dynamics have neither been critically tested nor acknowledged as a confounder or study limitation. Particularly with MTDR FM such effects have been reported in the field (PMID 11164242).
Reponses: Thank you for your valuable comments. In our previous study (Y-R. Yu et al., 2021, Nature Immunology), we utilized MDR and MG staining to distinguish MDR/MGhigh and MDR/MGlow populations from tumor-infiltrating lymphocytes (TILs) and ex vivo-induced T cells with dysfunctional mitochondria. These populations were subsequently sorted, and their mitochondrial ultrastructure was examined using electron microscopy. Our data demonstrated that MDR/MGlow T cells exhibited disorganized cristae morphology, supporting the notion that this population represents cells with mitochondrial dysfunction. Moreover, we also applied inhibitors to assess how these staining can distinguish cellular defects in mitochondrial health in the study published in 2021. Furthermore, we confirmed the terminally exhausted phenotype of MDR/MGlow T cells through a rechallenge assay.
In the current study, we have taken additional steps to ensure the robustness of our findings. Beyond using MDR and MG staining to identify terminally exhausted T cells, we have incorporated well-established exhaustion markers, including TCF1, TIM3, PD1, and CD39 (as now highlighted in the revised manuscript). These markers further substantiate our conclusion that mitochondrial protein degradation leads to increased RH levels, which correlate with the severity of T cell exhaustion. We appreciate the opportunity to clarify these points and hope this addresses the reviewer's concerns.

Whilst I appreciate the authors response, it does not fully address this point, which should be stated as a limitation in the discussion.

C-D. It is unclear why these proteomics experiments were performed in the presence of CHX, textual clarification and justification are needed. Using CHX as a main condition in the comparisons might introduce mitochondrial bias if cells have elevated rates of active mitochondrial translation. Without the cytoplasmic protein input into the mitochondrial proteome, an unfolded protein response in the mitochondria may be activated and the magnitude of this activation might differ between cell types tested, introducing bias. So, the authors should compare mitochondrial translation rates between depolarised vs healthy cell populations. Ideally, they should also repeat the proteomics experiments with a condition containing mitochondrial translation inhibitor like chloramphenicol, or using a SILAC based methodology.
Reponses: We thank the reviewer for their detailed and thoughtful comments regarding the use of CHX in our experiments and the potential of mitochondrial bias. The rationale for applying CHX in our experimental design was to block general protein translation and assess the stability of pre-existing proteins between MDR/MGlow and MDR/MGhigh T cells. As the reviewer mentioned, the different mitochondrial translation rate may be a confounding factor of our experiment. However, only 13 mitochondria-encoded proteins are translated by the mitochondrial translation machinery (Nature. 1981 Apr 9;290(5806):457-65), including MT-CO1, MT-CO2, MT-CO3, MT-ATP6, MT-ATP8, MT-ND1, MT-ND2, MT-ND3, MT-ND4, MT-ND5, MT-ND6, MT-ND4L, and MT-CYB. Among these, we observed a significant difference only in MT-CO2 levels between the MDR/MGlow and MDR/MGhigh groups in the steady state, while the other mitochondria-encoded proteins did not exhibit notable variations. Given this observation, we believe that differential mitochondrial translation is unlikely to be a major contributing factor to the differences between MDR/MGlow and MDR/MGhigh T cells. While we did not perform these additional experiments, we believe the current results derived from different approaches, including proteomics, mito-degron reporter, and release of regulatory heme, provide sufficient evidence to support our conclusions. We hope this explanation adequately addresses the reviewer's concerns and are happy to provide additional information if required.

Thank you.

In new Fig 2d, plotting a regression line is not appropriate here and a Kruskal–Wallis test should be used instead to examine

whether intergroup differences are statistically significant. Fig 2t would typically be analysed by 2-way ANOVA (as done in Fig 3d).

F-G. The legend indicates that the authors subjected cells to a combination of oligmycin A, antimycin A, Mdivi-1 and CHX treatment. Whereas, on the actual figure only CHX is mentioned and in the text describing these findings, none of these inhibitors were mentioned or acknowledged. Why were they used? This is an extremely potent cocktail and may have a major effect on the outcome of the experiment. Also, the split scale rather exaggerates the effect size, which is modest.
Reponses: Thank you for raising this important point. We sincerely apologize for not adequately explaining the rationale behind the use of oligomycin A, antimycin A, and Mdivi-1 in the original submission. These treatments were employed to induce mitochondrial dysfunction, as established in our previous publication (Yu et. al. Nature Immunology 2021) 12. We have clarified this in the revised manuscript.
Regarding the use of a split scale, we appreciate your feedback on the potential for exaggerating the effect size. To address this concern, we have revised the calculation method. Instead of using a division operation, we now use a subtraction operation to present the results. This adjustment also highlights the pronounced difference between mitochondrial protein degradation and cytosolic protein degradation in MDR/MGhigh and MDR/MGlow T cells. These updated results are shown in the revised Figure 1i-j. In addition, we also applied this approach in both murine and human CD8+ T cells with different exhaustion severity. Based on exhaustion severity, we also confirmed that terminally exhausted T cells preferentially degrade mito-degron reporter compared to progenitor exhausted T cells (revised Figure 1k-l).

Thanks - although the rationale for the cocktail needs to be mentioned in the main text.

H-I. Paired t tests are inappropriate here as the data are not paired (it would seem, assuming that each subset was separately repetitively stimulated). Further, the data has been normalized so it is no longer normally distributed. Were inhibitors (e.g. oligomycin etc) used in I?
Reponses: Thanks for the thoughtful question. To clarify, for original Figure 2h (revised Figure 2a), T cells were treated with oligomycin A, antimycin A, and Mdivi-1, followed by staining with MDR and MG. Flow cytometry analysis revealed two distinct populations: MDR/MGhigh and MDR/MGlow T cells. These populations were subsequently sorted from each sample and subjected to the heme assay. We examined the regulatory heme levels in both MDR/MGhigh and MDR/MGlow T cells from the same mouse as a paired comparison. For Figure 2i (revised Figure 2b), human T cells expressing NY-ESO-1-specific TCR were repetitively stimulated with tumor cells expressing the NY-ESO-1 antigen. This process also resulted in MDR/MGhigh and MDR/MGlow populations, which were sorted for further heme assay. We examined the regulatory heme levels in both MDR/MGhigh and MDR/MGlow T cells from the same donor as a paired comparison. Since both datasets were generated from the same mouse or human donor and involved sorting populations within each sample, we used paired analysis to confirm the increased of RH is a consistent outcome. In response to your comment regarding statistical tests, we have made revisions to ensure appropriate analysis. For Figure 2h (now revised as Figure 2a), we replaced the paired t-test with the Wilcoxon test to account for the normalized data. For Figure 2i (now revised as Figure 2b), we retained the paired t-test.
Lastly, regarding the experimental setup for Figure 2i, no inhibitors (e.g., oligomycin A, antimycin A, or Mdivi-1) were used. The MDR/MG low population in this experiment was induced solely by repetitive stimulation with tumor cells expressing the NY-ESO-1 antigen. This process also resulted in MDR/MGhigh and MDR/MGlow populations upon multiple round of re-stimulation (Reviewer Figure 8). Overall, we generated T cells accumulating depolarized mitochondria with three methods for assessing RH levels and draw our conclusion. We appreciate your feedback and believe these updates and clarifications address your concerns. Moreover, we hope you also agree that we took a high standard to make our conclusion by using different approaches.
Reviewer Figure 8. The staining of MDR and MG in human CD8+ T cells expressing TCR against NY-ESO-1 under repetitive TCR stimulation.

Thank you.

K. What is the P value signifying? Regardless, the biggest issue is that the decrease in relative RH amount following MG132 treatment occurs at comparable rates between cell types, contradicting earlier findings suggesting MGlow cells have higher rates of proteasomal liberation of RH from mitochondria.
Reponses: Thank you for your valuable comments. We performed a Simple Linear Regression analysis on this data, and the p-value indicates that the slope is significantly non-zero. The statistical analysis revealed that the slope for the MDR/MGlow population is -24.1, whereas the slope for the MDR/MGhigh population is -18.21. These results demonstrate that the MDR/MGlow population exhibits a higher rate of decrease in relative RH concentration following MG132 treatment. We sincerely apologize for not including the slope information in the original submission. We have now added this explanation and the corresponding statistical analysis to the revised manuscript (revised Extended Data Figure 2c). Additionally, we would like to clarify that both MDR/MGhigh and MDR/MGlow population can have hemoprotein turnover originating from cytosolic proteins. However, the turnover amount is significantly lower compared to the mitochondrial protein degradation. We appreciate your careful review and hope this clarification, along with the updates to the manuscript, addresses your concerns.

The P value in the test is not comparing the two conditions (from what I can see). It would make more sense to do a 2-way ANOVA (as done elsewhere), as I do not see the specific reason a linear regression fit is done here, and not in the many other examples of dose-response experiments in the manuscript.

Related to Figure 3.

Extended Data Fig. 3B - paired t test used inappropriately
Reponses: Thank you for pointing this out. The results in original Extended Data Figure 3b (revised Extended Data Figure 3d) represent the RH concentration of tumor interstitial fluid (TIF) and serum from the same tumor-bearing mouse. Thus, the nature of this data is indeed paired. However, since the data were normalized, we have replaced the paired t-test with the Wilcoxon test, which is more appropriate for normalized data. We have updated the statistical analysis method and revised the figure legend to reflect this change in the revised manuscript. We appreciate your feedback and have made the necessary adjustments in the revised manuscript.

Although the measurements are from the same mouse, they are different variables that are not paired (e.g. not 'before-after' from the same sample), and so an unpaired statistical test should be used e.g. Mann-Whitney U test.

The statement one line 227 that ‘these findings indicate that the degradation of mitochondrial proteins increase RH levels, which correlates with the severity of T cell exhaustion’ has not been substantiated.
Reponses: We appreciate the reviewer’s thoughtful comments. In the revised manuscript, we have expanded our experiments to substantiate this statement. Specifically, we generated human TPEx (progenitor exhausted T cells) and TTEx (terminally exhausted T cells) and performed proteomic analysis, which revealed that TTEx exhibits higher proteasome activity compared to TPex (revised Figure 1c). This finding was further confirmed using a proteasome activity probe (revised Figure 1d-e). Moreover, we conducted new experiments to determine regulatory heme levels in those human T cell subsets and confirmed that TTEx have higher RH levels compared to TPex (revised Figure 2e). These new results are consistent with our findings that murine TTEx have higher RH levels compared to TPex (revised Figure 2d).
To investigate mitochondrial protein degradation, we employed both the mitochondrial protein degradation reporter (mito-degron) and the cytosolic protein degradation reporter (cyto-degron) in a murine tumor model (revised Figure 1k) and a human ex vivo exhaustion model (revised Figure 1l). These results demonstrated that TTEx degrades more mitochondrial reporter compared to TPEX. Taken together, we believe the revised manuscript provides robust evidence that mitochondrial protein degradation increases RH levels, correlating with the severity of T cell exhaustion. We hope this explanation adequately addresses your concerns.

Thank you.

Related to Figure 4.

C. This experiment needs to be performed on Bach2 mutant cells to see if the increase in Blimp1 is prevented.
Reponses: Thanks for the advice. We transduced the Bach2WT and Bach2MUT into P14 cell from Bach2CKO mice, and induced the exhaustion of T cell by using αCD3 antibody restimulation with or without hemin treatment for 48 hours. We then assessed Blimp1 expression by flow cytometry. Our result confirmed that hemin induces Blimp1 expression in P14 cells overexpressing wild type Bach2 (Bach2WT), while failing to upregulate Blimp1 expression in P14 cells overexpressing mutant form of Bach2 (Bach2MUT). These results have been include in the revised manuscript (revised Figure 3c and Extended Data Figure 3l).

Thank you. I assume revised Fig 3d is referred to here.

Extended Data Fig. 4A-D. Inhibitors were used for unjustified reasons.
Reponses: Thank you for raising this point. We sincerely apologize for not adequately explaining the rationale behind the use of oligomycin A, antimycin A, and Mdivi-1 in the original submission. These treatments were employed to induce mitochondrial dysfunction, as established in our previous publication (Yu et. al. Nature Immunology 2021) 12. We have clarified this in the revised manuscript (revised Extended Data Figure 3a-d) as discussed above.

Thanks.

In extended data figure 4 - all the statistical tests here are wrong (assuming Wilcoxon signed rank test referred to). None of the samples in here are paired since they come from separate individual mice.

Related to Figure 5.

Extended Data Fig. 5c-j. Again the statistical test used here is appropriate as these data are neither paired nor normally distributed (e and g).
Reponses: Thank you for your insightful comments. The results in original Extended Data Fig. 5c, 5e, and 5g represent the percentage of IFNg/TNFa-producing TILs (c), as well as the expression levels of Bach2 (e) and Blimp1 (g) in P14 T cells expressing either control gRNA or Pgrmc2-targeting gRNA, isolated from the same tumor in mice that received co-transfer of both P14 cells. Since we performed co-transfer in each mouse and directly compared control gRNA-expressing and Pgrmc2-targeting gRNA-expressing P14 cells isolated from the same tumors, these results represent paired comparisons. However, as the data were normalized, we have replaced the paired t-test with the Wilcoxon test, which is more appropriate for normalized data. We have updated the statistical analysis method accordingly and revised the figure legends to reflect this change.

Thanks. Again, with relation to paired t tests, the assumption is that the control and Pgrmc-2 P14 T cells are generated from the same mouse, which I assume to be the case. Even then, it is stretching it to assume that the data can be analyzed in a pairwise fashion.

Related to Figure 6.

The use of paired t tests is not appropriate as the data are not paired, and sometimes also not normally distributed. Why are points joined?
Reponses: Thank you for your thoughtful comments. The experiments presented in the results of original Figure 6 (revised Figure 5) were co-transfer experiments as described in the previous point. Since we performed co-transfer in each mouse and directly compared control gRNA-expressing and Nr1d1/2-targeting gRNA-expressing P14 cells isolated from the same tumors, these results represent paired comparisons. We also incorporated new results regarding to Bmal1-deficiency with similar designs in the revised manuscript. To illustrate the paired nature of the data and experiemtns, we joined the points corresponding to the same tumor or mouse. Additionally, as the data were not normalized, we believe the assumption of normal distribution holds, making the use of the paired t-test appropriate in this context.

As before, its somewhat tenuous to consider the co-transferred cells as paired, but if that is the case, then pairwise t tests should not be used in analyzing Bmal1 cKO experiments in Figure 5 as these are clearly from different mice.

Referee #4

(Remarks to the Author)
This manuscript provides intriguing insights into how proteasome activity regulates T cell exhaustion, linking mitochondrial dysfunction and heme-mediated signaling with transcriptional reprogramming and immunotherapeutic failure. The study is ambitious in scope and addresses timely concepts in immunometabolism and T cell biology. However, significant mechanistic and explanatory gaps remain. My main concerns follow:

1) While the authors present compelling data that proteasome activity is upregulated in exhausted $CD8^+$ T cells and correlates with degradation of mitochondrial proteins, the mechanistic basis for this selectivity is not addressed. The authors do not provide biochemical, structural, or post-translational data to explain why mitochondrial targets—including hemoproteins—are specifically recognized by the proteasome. This omission leaves a key conceptual gap in the central model.

2) The proposed pathway implies that dysfunctional mitochondria trigger proteasome engagement. However, the upstream signaling axis responsible for this upregulation is neither tested nor hypothesized. It is unclear whether mitochondrial depolarization itself initiates proteasome activation or whether this is a consequence of an independent exhaustion-associated program.

3) The inclusion of circadian regulators such as REV-ERBα/β and BMAL1—while conceptually interesting—feels peripheral to the central heme-proteasome axis. These elements are not tested or shown to be essential for the phenotypes described. The authors may consider either strengthening this arm experimentally. The authors mention that heme alters the transcriptional activity of these circadian factors based on transcriptomic trends and gene knockout phenotypes. However, this lacks direct biochemical validation that regulatory heme binds to and functionally modulates these transcription factors. The following experiments could help address these points: a) Direct binding assays demonstrating interaction between heme and circadian TFs (REV-ERBs, CLOCK, or BMAL1). b) Reporter assays or chromatin occupancy analyses showing that heme modulates the transcriptional output of circadian targets in T cells. c) Rescue experiments using heme-binding mutants of these TFs to establish causality.

4) Although the paper introduces RH as a mediator of transcriptional reprogramming (via Blimp1 induction and Bach2 degradation), key questions remain unanswered: What quantity or localization of RH is required to alter T cell fate? How is

RH imported into the nucleus and how stable is it under exhaustion conditions? Are alternative pathways of heme degradation/export (e.g., HO-1, FLVCR1) active in T cells or the TME?

5) The rationale for the last section of the paper -CD19 CARs – is relatively weak. Why would the CAR T cells become exhausted during manufacturing? Why would they exhibit proteasome dysregulation in this context? Isn't this induced by the TME or the general cancer macroenvironment?

6) The effects of bortezomib pre-treatment on CAR-T cell function are modest, with some improvement in tumor burden and survival. Importantly, minimal evidence is provided that the same exhaustion or proteasome dysregulation observed in TILs occurs during CAR-T manufacturing. This is a critical assumption upon which the therapeutic implications rest. The authors should directly measure proteasome activity, mitochondrial fitness, and exhaustion markers in CAR-Ts during ex vivo expansion and progressively post-transfer. Also, it is important to note that Reference #61 compromises the therapeutic novelty of the current study.

7) Unclear how and why short-term pre-treatment with bortezomib could induce long-lasting effects in the transferred CAR-T cells. Could RH-edited CAR T cells be tested to define if they show improved therapeutic efficacy in vivo?

Additional Comments
• Please clarify whether immunoproteasome components are directly implicated.
• The manuscript has several typos and grammar errors that need attention.

Referee #5

(Remarks to the Author)
The results presented do not demonstrate sufficient novelty to meet the publication standards for Nature. In particular, the in vivo data lack robustness (see reviewer Figure 7). Greater mechanistic insight may be needed to support the conclusions. The novelty here is not the connection of heme to Bach1 in T cells (Gupta Blood 2023). The central novel claim could be that mitochondrial depolarization leads to increased proteasome activity, which selectively degrades mitochondrial proteins to elevate heme levels. However, the mechanism behind this is unclear. Proteasome function is ATP-dependent, so it is surprising that membrane depolarization, typically associated with mitochondrial ATP depletion, would enhance proteasome activity. Furthermore, the basis for the selective degradation of mitochondrial proteins is not well explained. Moreover, the connection to clock genes is not clear. Heme-BACH1 makes sense but not sure how they got to BMAL1.

Version 2:

Reviewer comments:

Referee #3

(Remarks to the Author)
I am satisfied that the authors have addressed my comments.

Referee #4

(Remarks to the Author)
the authors have satisfactorily responded to most of my comments and concerns. The paper has improved and is now more cohesive and mechanistic. I would respectfully suggest changing the tittle to "contribute" instead of "drive" and justifying more thoroughly the transition from the analysis of this pathway in TILs vs. CAR-T cell manufacturing in the main text. Overall, this is an interesting and exciting story.

Referee #5

(Remarks to the Author)
The authors have done a good job of responding.

Open Access This Peer Review File is licensed under a Creative Commons Attribution 4.0 International License, which permits use, sharing, adaptation, distribution and reproduction in any medium or format, as long as you give appropriate credit to the original author(s) and the source, provide a link to the Creative Commons license, and indicate if changes were made.
In cases where reviewers are anonymous, credit should be given to 'Anonymous Referee' and the source.
The images or other third party material in this Peer Review File are included in the article's Creative Commons license, unless indicated otherwise in a credit line to the material. If material is not included in the article's Creative Commons license and your intended use is not permitted by statutory regulation or exceeds the permitted use, you will need to obtain permission directly from the copyright holder.
To view a copy of this license, visit https://creativecommons.org/licenses/by/4.0/

Response (Xu et al.)

We would like to thank the reviewers for the constructive and insightful comments to our manuscript. Based on the comments and critiques, we propose some experiments and attempt to answer these comments point by point in the letter. We think that the constructive comments raised by the reviewers indeed strengthen the depth of this review article. We hope these proposed experiments and replies in this letter can satisfy the reviewers' concerns.

Please find our "point-to-point" response (in blue) to the reviewer's comments below. We also highlighted modifications in blue and marked the new figures with blue labeling in figure number in the revised manuscript.

Referees' comments:

Referee #1 (Remarks to the Author):

In their manuscript, Xu et al. report that the previously known accumulation of depolarized mitochondria in exhausted t cells leads to heme accumulation after degradation of mitochondrial proteins and that heme binds to Bach2, leading to increased expression of Blimp1, which further promotes t cell exhaustion. Additionally, the authors found that several circadian clock molecules are heme proteins and therefore also involved in T cell exhaustion. This study provides another layer of mechanistic insight within the previously reported spectrum of mechanisms by which mitochondrial depolarization engaged transcriptional networks of t cell exhaustion. It links the metabolic phenomenon of accumulation of depolarized mitochondria to heme, which is linked to Blimp1, thus proposing a new target for immunotherapies in cancer.

Major issues:

1. The role of heme for T cell exhaustion in the situation of CAR T cell therapies has previously been published (Gupta et al. Blood 2023). It would be helpful if the authors could delineate their findings from previously published reports in their manuscript, given close similarities as well as discrepant findings. It would also be helpful to cite this study.

Response: We sincerely appreciate the reviewer's insightful feedback. We believe that the study referred by the reviewer is a meeting abstract from ASH 65th. The abstract by Gupta et al. reports that during ex vivo CAR-T cell manufacturing under non-stressful conditions (normoxia, nutrient sufficiency, and the absence of repetitive TCR stimulation), heme supplementation significantly decreased the proportions of LAG3^{+} and TIM3^{+} cells, as well as CD62L^{+} and TCF1^{+} cells, in both CD4^{+} and CD8^{+} subsets. Additionally, their study evaluated the cytotoxicity and cytokine production of CAR-T cells co-cultured with target cells, revealing that Hemin-pretreated CAR-T cells exhibited enhanced cytokine production and greater killing capacity. Although this meeting abstract suggests that heme may modulate T cell differentiation, we believe that the observed phenotype represent a steady-state feature, as those CAR T-cells did not undergo repetitive TCR stimulation- a critical process encountered by tumor-specific T cells within the tumor microenvironment. In contrast, we employed a different experimental

design to better mimic repetitive TCR stimulation, a process tumor-specific T cells could encounter in tumors. Specifically, $CD8^{+}$ T cells were primed and then differentiated into effector T cells for six days and then treated with hemin plus anti-CD3 antibody for 48 hours (**revised Extended Data Figure 2g**). Our data underscores that higher heme concentrations accelerate T cell exhaustion in the condition that T cells undergo repetitive TCR stimulation (**revised Figure 3**). To further clarify whether repetitive TCR stimulation is a key factor to determine how heme can differentially instruct T cell differentiation, we also treated effector T cells with hemin during resting phase for 48 hours. Our results showed partial similarity to those of Gupta et al. (ASH 65th). Treatment with hemin in the resting condition reduced $TIM3^{+}PD1^{+}$ population and CD62L-expressing subset. However, we did not observe declined $TCF1^{+}$ population and enhanced cytokine production in hemin treated T cells (**Reviewer Figure 1**). Normally, cells with functional mitochondria and intact mitochondrial dynamics can utilize available heme to enhance cellular metabolism. However, repetitive TCR stimulation promotes the accumulation of depolarized mitochondria and disrupts mitochondrial dynamics. We thus speculate that, under these conditions, cells may engage distinct mechanisms to respond excessive heme levels. Together, these results suggest that the intricate interplay between heme availability and distribution, mitochondrial health status, and T cell receptor signal amplitude can differentially influence T cell differentiation. We have incorporated part of this discussion into the revised manuscript and hope the reviewer agrees that our experimental setting provides a more patho-physiologically relevant model to mimic the challenges encountered by tumor-specific T cells within tumors.

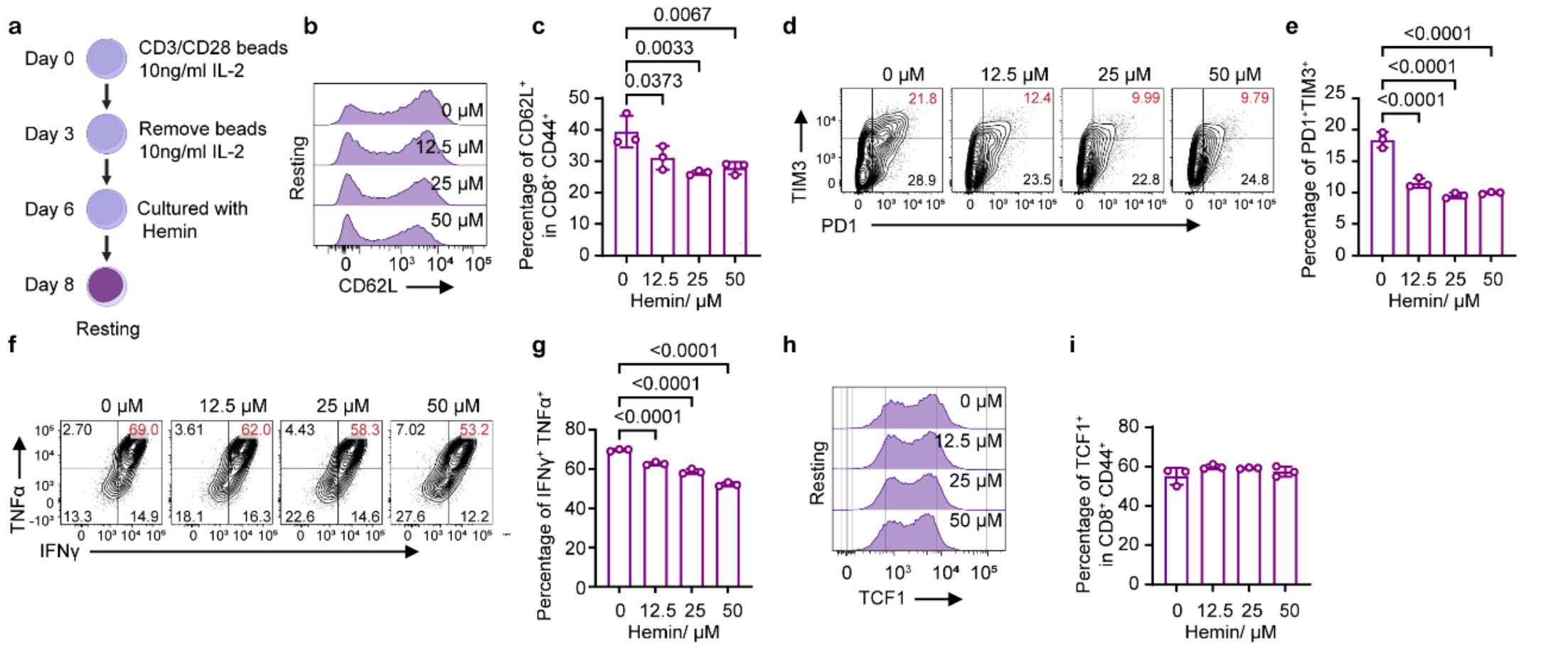

Reviewer Figure 1. The features of resting $CD8^{+}$ T cells treated with Hemin.

a, Experimental workflow for in vitro treatment of resting $CD8^{+}$ T cells with Hemin. **b,** Representative flow plots of CD62L expression of the resting $CD8^{+}$ T cells. **c,** The quantification of CD62L expression in (b). n = 3. **d,** Representative flow plots of PD1 and TIM3 expression in the resting $CD8^{+}$ T cells. **e,** The quantification of $PD1^{+}TIM3^{+}$ population in (d). n = 3. **f**, Representative flow plots of cytokine production in the resting $CD8^{+}$ T cells. **g,** The quantification of $IFN\gamma^{+}TNF\alpha^{+}$ population in (f). n = 3. **h,** Representative flow plots of TCF1 expression in the resting $CD8^{+}$ T cells. **i,** The quantification of TCF1 expression in (h). n = 3. Data are representative of three independent experiments with similar results (c, e, g and i). Each symbol represents one individual. Data are means ± s.d. and were analyzed by one-way ANOWA (c, e, g and i).

2. The annotation of different exhausted t cell subsets for subsequent correlation with proteasome-related genes, follows a biased approach. It would be helpful to also include

controls, such as naïve T cells from the blood, on the negative side of the spectrum of exhaustion. It seems that the evidence for the conclusion that exhausted t cells have increased proteasome activity comes only from the public scRNAseq data. Overall, the correlation of exhaustion with actual proteasome activity requires experimental validation.

Response: We sincerely appreciate your insightful feedback and suggestions. In response to your comments, we have conducted additional experiments with human T cells to provide robust evidence supporting our assertion that terminally exhausted T cells exhibit higher proteasome activity. Below, we detail the experimental design and findings:

a. Proteasome Activity Assay:

To investigate proteasome activity across different T cell states, we activated human CD8 T cells with distinct activation procedure to obtain T_E (Effector T cells), T_M (Memory T cells), and T_{PEx} (Progenitor Exhausted T cells) (**revised Extended Data Figures 1d**). We used a proteasome activity probe to measure proteasome activity in those human CD8 T cell subsets. This new result indicates that T_{TEx} and T_M exhibited higher proteasome activity than T_{PEx} and T_E (**revised Figures 1d-e**). Moreover, we also applied the same probe for TILs isolated from melanoma-bearing mice. Consistent with our findings, proteasome activity probe increased signal intensity along with the severity of exhaustion and Tpex subset has the lowest proteasome activity (**Reviewer Figure 2**).

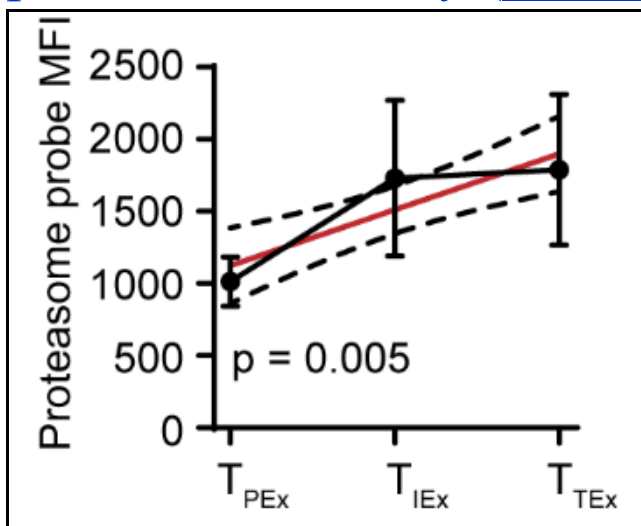

Reviewer Figure 2. Quantification of proteasome probe fluorescence intensity in the indicated subtype of P14 TILs derived from YUMM1.7-gp33 melanoma tumors. n = 11; Data are the cumulative results from two independent experiments. Data are means ± s.d. and were analyzed by simple linear regression.

b. Proteomic analysis:

In addition to applying proteasome activity probe, we also performed proteomic analysis with these human T cell subsets along with naïve T cells (T_N) to determine their proteome with quantitative measurement. Enrichment analysis of the differentially expressed proteins revealed that T_{TEx} and T_M exhibited significantly higher proteasome scores compared to T_N (**revised Figures 1c**), which aligns with the result of proteasome activity probe.

c. Protein Degradation Reporter Assay:

To further explore proteasome-mediated protein degradation, we developed lentivirus harboring mitochondrial protein degradation (Mito-degron) and cytoplasmic protein degradation (Cyto-degron) reporters for human T cells. This analysis revealed that T_{TEx} displayed significantly higher mitochondrial protein degradation activity compared to T_{PEx} (**revised Figure 1k-l**). Moreover, although T_M exhibited the highest overall proteasome activity, T_M preferentially degrade Cyto-degron rather than Mito-degron (**revised Extended Data Figure 1j**).

These new dataset with human T cells and proteasome activity staining in murine TILs further strengthen our conclusion that proteasome activity gradually increases as T cells progress toward terminal exhaustion state.

3. The authors start out with human CAR-T cell data, highlighting the association of the proteasome with exhaustion using single-cell transcriptomic re-analyses but then switch to mouse cells for the next mechanistic steps. It would strengthen the story if the proposed heme-bases mechanism of T cell exhaustion could also be tested in human T cells given that they even had access to clinical samples from patients with CAR T cell therapy. But also basic tests with ex vivo isolated and cultured (i.e. repetitive restimulations) human CD8 T cells would be helpful to evaluate the relevance of the findings for immunotherapies, as proposed by the authors.

Reponses: We thank the reviewer's valuable comments. In the revised manuscript, we examined the regulatory heme (RH) levels in human T_N, T_E, T_M, T_{PEx}, and T_{TEx} subsets generated as described in point 2. Our results showed that T_{TEx} exhibits higher RH levels compared to T_{PEx} or T_E (**revised Figure 2e**). Notably, T_M had lower RH levels compared to T_{TEX} despite that both subsets had similar staining levels of proteasome activity probe. This is consistent with the results of degron reporter, suggesting that T_M cells mainly degrade cytosolic proteins but T_{TEX} selectively degrade mitochondrial proteins. Unfortunately, we can not examine whether RH levels in CD19 CAR-T cells are different among patients with distinct therapeutic outcomes since the measurement of RH requires a high cell number. To further strengthen our conclusion in human contexts, we performed a comprehensive analysis on whether proteasome inhibition can improve human CD19 CAR-T cells resistance to exhaustion and therapeutic outcomes in NSG mice engrafted with human tumors. Moreover, we have moved CAR T-cell data to figure 6 rather than the first figure in the revised manuscript to improve the cohesion and connectivity of mechanistic understandings and clinical implications. Altogether, we hope the reviewer agrees that the addition of new results combined with our results using murine T cells do provide advanced information and proof-of-concept evidence on targeting proteasome activity during CAR T cell production.

4. The title of the manuscript highlights heme as a metabolic checkpoint. The manuscript, in its current form, is quite vague with respect to mechanistically testing the role of heme in T cell metabolism (so far primarily omics and bioinformatics). It would be helpful to see how it directly affects selected major metabolic pathways that have previously been associated to T cell exhaustion/stemness with experimental evidence.

Reponses: We appreciate the insightful comments raised by the reviewer regarding the manuscript title and the mechanistic role of heme in modulating T cell metabolism. We have revised the title of this manuscript to better reflect the focus of this work. In addition, we conducted new experiments to examine the roles of heme on modulating metabolic program in T cells. First, under repetitive stimulation, we observed that exogenous hemin supplementation significantly suppressed mitochondrial-dependent metabolism (**Reviewer Figure 3**). Moreover, our new experiment demonstrated that Bmal1-deficient T cells maintain their dependence on mitochondria-driven metabolism (**Revised Figure 5j**) and mitochondrial health, as assessed by MDR and MG staining (**Reviewer Figure 4**), in response to exogenous hemin treatment. These new data align with the reported metabolic characteristics that T_{TEx} cells lose their ability to produce ATP via OXPHOS [1] and support our claim that heme can affect metabolic program by modulating circadian regulatory circuit in T cells. Together, we believe that our results suggest that the increase of regulatory heme due to accumulation of depolarized mitochondria can drive

a vicious cycle to further dampen mitochondrial health in T cells, which further promotes severity of exhaustion. We hope the reviewer agrees that our claim is supported by these new data and our revised title improves the clarity of our findings.

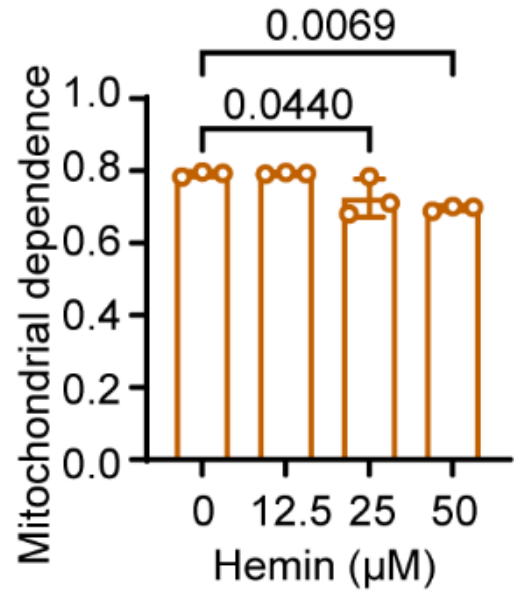

Reviewer Figure 3. Quantification of mitochondrial metabolic preference in CD8^{+} T cells treated with indicated concentrations of Hemin under repetitive anti-CD3 antibody stimulation, assessed by single-cell energetic metabolism profiling through translation inhibition (SCENITH). n = 3; Data are representative of three independent experiments with similar results. Each symbol represents one individual. Data are means ± s.d. and were analyzed by one-way ANOWA.

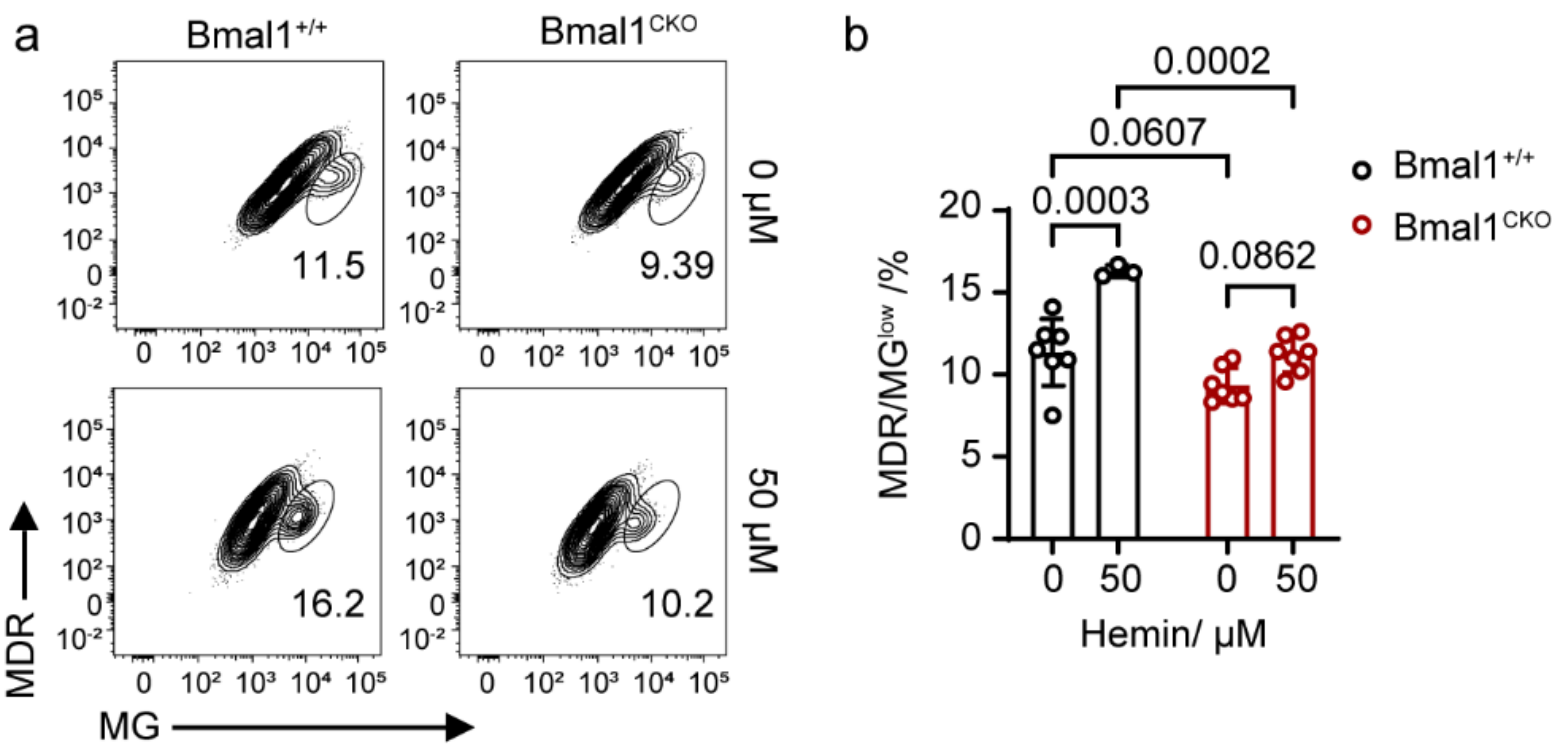

Reviewer Figure 4. a, Representative flow plots of MDR and MG staining in the Bmal1$^{+/+}$ or Bmal1^{CKO} CD8^{+} T cells under αCD3 antibody repetitive restimulation with or without Hemin treatment. **b,** Quantification of MDR/MG^{low} population in (a). Data are representative of one independent experiments. Each symbol represents one individual. Data are means ± s.d. and were analyzed by two-way ANOWA.

5. In Fig.2 it would be helpful to not only sort the TILs according to their mitochondrial phenotypes but also according to a cellular classification of exhaustion vs. stemness to directly make the association of proteasomal degradation with t cell exhaustion (not only indirectly via mitochondrial states). Data that follows in fig. 3 would be helpful to support Fig. 2.

Reponses: We sincerely thank the reviewer for their valuable suggestions, which indeed is important for strengthening our conclusion. As suggested by the reviewer, we conducted new experiments listed below.

a. Degron assay in murine T cells:
   We transduced mito-mCherry degron and cyto-mCherry degron reporters into P14 cells expressing a TCF7-GFP reporter and then transferred transduced P14 cells into tumor-bearing mice. In this setting, TILs with different exhaustion severity, including TCF7-GFP^{+}PD1^{+}TIM3^{-} (T_{PEx}) and TCF7-GFP^{-}PD1^{+}TIM3^{+} (T_{TEx}) subsets, were then isolated. These cell subsets were treated with or without cycloheximide (CHX) for another 2 hours to measure degron signals. This design allowed us to assess the relationship between the exhaustion state in TILs and their mitochondrial or cytosolic protein degradation. Our results revealed that the T_{TEx} subset displayed higher reduction of mito-mCherry degron signal in response to CHX compared to T_{PEx} subset. However, the reduction of cyto-mCherry degron in response to CHX was comparable between T_{PEx} and T_{TEx} (**revised Figure 1k**). These new results strengthen our conclusion that

terminally exhausted T cells (T_{TEx}) selectively degrade mitochondrial proteins in a higher kinetics compared to progenitor exhausted T cells (T_{PEx}).

b. Degron assay in human T cells:
We also examined the degron reporter in the human T cell setting. We first generated mito-mCherry degron and cyto-mCherry degron reporters in lentivirus vectors. We transduced these reporters into ex vivo cultured human T cells, which were then stimulated with repetitive TCR stimulation to induce exhaustion. Additionally, IL-2- or IL-15-skewed T_E and T_M cells were generated as described in our response to the second question raised by the reviewer. These cells were also treated with or without CHX for 2 hours to measure mCherry signals. Our result is consistent with our observation in murine TILs, showing that T_{TEx} subset has a higher reduction of mito-mCherry reporter compared to T_{PEx}, while the degradation of cyto-mCherry reporter remained comparable between T_{TEx} and T_{PEx} (**revised Figure 1i**).

c. Proteasome Activity Assay:
To investigate proteasome activity across different T cell states, we utilized human T cells to generate T_E (Effector T cells), T_M (Memory T cells), and T_{PEx} (Progenitor Exhausted T cells). We then applied a proteasome activity probe to measure proteasome activity in those human CD8 T cell subsets. This new result indicates that T_{TEx} and T_M exhibited higher proteasome activity than T_{PEx} and T_E (**revised Figures 1d-e**). Moreover, we also applied the same probe for TILs isolated from melanoma-bearing mice. Consistent with our findings, proteasome activity probe increased signal intensity along with the severity of exhaustion and Tpex subset has the lowest proteasome activity (**Reviewer Figure 2**, see above).

d. Proteomic analysis:
In addition to applying proteasome activity probe, we also performed proteomic analysis with these human T cell subsets along with naïve T cells (T_N) to determine their proteome with quantitative measurement. Enrichment analysis of the differentially expressed proteins revealed that T_{TEx} and T_M exhibited significantly higher proteasome scores compared to T_N (**revised Figures 1c**), which aligns with the result of proteasome activity probe.

Altogether, we hope the reviewer agrees that these updated results further corroborate our conclusion that terminally exhausted T cells, whether defined by specific markers or the declined mitochondrial health, exhibit greater mitochondrial protein degradation compared to T_{PEx}.

6. The data that heme regulates circadian rhythm molecules is exciting. However, these data are quite disconnected from the logical flow of the story and seem to be a stand-alone topic in the current state of the paper. It would be helpful to elaborate more experimentally on the role of the proposed molecules for exhaustion, metabolism and anti-tumor immunity.

Reponses: We sincerely appreciate the reviewer’s positive feedback on our findings regarding regulators of circadian rhythms and their impacts on T cell exhaustion. Following the reviewer’s recommendation, we performed additional experiments to further elucidate how regulators of circadian rhythms influence T cell exhaustion, metabolism, and anti-tumor

immunity. Below, we summarize the new findings:

a. Role of Nr1d1/2 on guiding exhaustion:

Our integrated analysis suggested that the progenitor exhausted T cells and terminally exhausted T cells have different activity of Reverbα/β (encoded by Nr1d1/2), two transcriptional repressors for regulating circadian rhythms (**revised Figure 6a**). To investigate the roles of Nr1d1/2 on T cell exhaustion, we generated Nr1d1/2-deficient P14 T cells with the CRISPR-based approach and evaluated their differentiation and metabolic fitness in a murine tumor model. We found that Nr1d1/2-deficient P14 cells exhibited enhanced mitochondrial fitness (**revised Figure 5b-c**), increased dependence on mitochondrial-dependent metabolism (**revised Figure 5d**), an elevated propensity to form progenitor exhausted T cells (**revised Figure 5e**), and elevated cytokine production (**revised Figure 5f-g**) compared to wild type P14 cells. These new results are consistent with our original findings, indicating that Bmal1-deficient P14 cells exhibit an enhanced ability to resist terminal exhaustion.

b. Heme failed to modulate T cell exhaustion and impair mitochondrial health in Bmal1-deficient T cells:

We confirmed that Bmal1-deficient T cells expressed lower levels of Nr1d1/2 expression (**revised Figure 5h**), consistent with previous reports showing that Bmal1 controls the expression of Nr1d1/2 in muscle cells and hepatocytes. Furthermore, we found that hemin treatment suppressed mitochondrial-dependent metabolism in T cells derived from wild type mice but not in those from Bmal1-deficient mice under repetitive TCR stimulation (**revised Figure 5i**). In this in vitro repetitive TCR stimulation setting, hemin treatment also resulted in a reduced induction of terminally exhausted T cells, as assessed by the simultaneous expression of PD-1 and TIM-3, in Bmal1-deficient T cells (**revised Figure 5j**). These new data are consistent with our original findings, showing that Bmal1-deficient tumor-infiltrating T cells had improved mitochondrial fitness (**revised Figure 5l**) and enhanced cytokine production (**revised Figure 5m-n**) in vivo.

c. Improved cellular functions in Bmal1-deficient T cells:

We also conducted adoptive transfer experiment to assess the anti-tumor responses provided by Bmal1-deficient P14 T cells. Our result showed that adoptive transfer of a low number of Bmal1-deficient P14 cells led to a modest but significant reduction in tumor growth compared to wild type P14 cells (**revised Figure 5o**). Of note, tumor infiltrating Baml1-deficient P14 cells had an increased abundance of T_{PEx} (**revised Figure 5p**). We further examined whether Bmal1-deficient T_{PEx} display more superior stem-like properties than wild type T_{PEx}. We co-transferred wild type and Baml1-deficient P14 cells in to YUMM1.7-gp33 melanoma-bearing mice and subsequently isolated tumor-infiltrating $CD127^+PD1^+TIM3^-$ P14 T cells. Equal number wild type and Bmal1-deficient tumor-infiltrating $CD127^+PD1^+TIM3^-$ P14 T cells were then mixed and co-transferred into naïve mice, followed by infection with LCMV Armstrong clone (**Revised Figure 5q**). Our new results showed that Bmal1-deficient memory-like P14 cells demonstrated higher ability to maintain TCF1 in the expanded populations, produce IFNg, and reduced expression of TIM3 (**Revised Figure 5r-t**). These new results highlight that targeting Bmal1 can preserve T cell anti-tumor

responses and enhance their stem-like properties in T_{PEX} subsets.

Together, our new results, in combination with our original findings, underscore that heme accelerates T cell exhaustion and exacerbates mitochondrial impairment by regulating key circadian rhythm molecules. We hope these new data sufficiently address the reviewer's comments and further substantiate the critical role of circadian rhythm regulators in T cell metabolism and function.

Minor issues.

1. The manuscript requires English editing. It is in several instances difficult to understand what the authors mean due to grammar and spelling issues.

Reponses: We sincerely appreciate the reviewer's feedback regarding the language and clarity of our manuscript. We have engaged a native English speaker to carefully edit and revise the manuscript to improve grammar, spelling, and overall readability.

2. The figure legends and methods lack details required to fully understand the experimental conditions and bioinformatic analysis. Figures contain colors (probably for highlighting certain aspects) without indicating their purpose (e.g. Fig.2b-d).

Reponses: We thank the reviewer for pointing out the need for additional details in the figure legends and methods, as well as clarification regarding the use of colors in the figures. We have carefully revised the figure legends to provide more comprehensive explanations of the experimental conditions and bioinformatic analyses. Additionally, we have updated the methods section to include detailed descriptions of all relevant procedures. We also clarified the colors appearing in those figures in the figure legends of the revised manuscript. We hope these updates address the reviewer's concerns and improve the clarity and interpretability of the figures and accompanying text.

3. Some figures contain statistical information, others not (e.g. violin plots of scRNAseq data). It would be helpful if p values can consistently be reported in the respective figures.

Reponses: We sincerely appreciate the reviewer's suggestion regarding the consistency of statistical reporting across the figures. We have now incorporated statistical information, including p-values, for all relevant data in the revised figures. We hope these revisions address the reviewer's concerns and enhance the clarity and completeness of our data presentation.

Referee #2 (Remarks to the Author):

In the manuscript entitled "Heme-mediated signaling acts as a metabolic checkpoint to orchestrate T cell exhaustion", Xu et al. show that terminally exhausted CD8 T cells express an enhanced proteasome gene signature compared to other Tex and non-Tex populations. Xu et al. reason that the increased proteasome gene signature is due to the accumulation of depolarized mitochondria in terminally exhausted CD8 T cells. The authors demonstrate that exhausted/CD8 T cells with depolarized mitochondria degrade more mitochondrial proteins leading to greater intracellular regulatory heme concentrations which they claim leads to impacts on different transcription factors that ultimately control T cell exhaustion.

The work is interesting but due to several major concerns I recommend this manuscript be rejected. The manuscript presents many different pathways that impact exhaustion by themselves but are loosely linked through intracellular heme release. The presentation of these pathways are not logically linked and there is some contradictory data about hemin treatment and Bach2. These issues, combined with some typos and odd grammatical errors, makes the manuscript difficult to follow. Even if these issues are addressed in a revision, this manuscript does not meet the level of novelty expected of Nature. In my opinion this work is more suitable for a more specialized immunology journal after substantial revision/restructuring.

Reponses: We sincerely appreciate the reviewer's feedback and recognize the concerns raised by the reviewer. Below, we address the main points in detail and provide evidence to support the novelty, logical coherence, and significance of our study.

1. Concern Regarding the Link Between Hemin Treatment and Bach2 Expression

We have clarified the relationship between hemin treatment and Bach2 expression in our response to the second question (see below). Specifically, we demonstrate that heme directly binds to Bach2, altering its expression and transcriptional repression activity. These results provide a mechanistic explanation of how mitochondrial dynamics and heme metabolism influence T cell differentiation and exhaustion. Additionally, we have revised the manuscript to improve clarity on this point and to avoid any perceived contradictions.

2. Novelty of the Study

We respectfully argue that our study provides significant novel insights into T cell exhaustion and how declined mitochondrial health orchestrates T cell exhaustion program, addressing key gaps in the current understanding of T cell biology:

a. Mechanisms governing transcriptional regulation in T cell exhaustion:
   While emerging evidence highlights the dynamic transition of transcriptional networks in T cell exhaustion, the mechanisms governing these transitions remain poorly understood. Our study is the first to report that the accumulation of depolarized mitochondria modulates Bach2 downregulation and Blimp1 upregulation through a heme-mediated regulatory axis. These findings establish a novel mechanistic link between declined mitochondrial health and transcriptional transitions during T cell exhaustion.
b. Proteasome-guided immunometabolic regulations for T cell exhaustion
   Although dysfunctional mitochondria have been implicated in promoting T cell exhaustion, the precise mechanisms linking mitochondrial dysfunction to the exhaustion program remain largely unknown. Our study reveals that T cells accumulating depolarized mitochondria enhance proteasome activity to selectively degrade mitochondria protein, thereby driving T cell exhaustion. This discovery unveils a previously unrecognized regulatory axis in the immunometabolism and highlights the role of proteasome activity in metabolic adaptation and cell fate determination.
c. Role of circadian rhythm regulators in T cell exhaustion:
   Circadian rhythms have been shown to affect therapeutic efficacy of immune checkpoint blockade in pre-clinical models and patients. However, whether circadian rhythm regulators modulate T cell differentiation and how this impacts T cell anti-tumor responses remain unknown. Through bioinformatic analyses and metabolic

assessments, we demonstrate for the first time that circadian rhythm regulators play a role in T cell exhaustion. Furthermore, we provide new evidence that heme influences T cell metabolism and exhaustion severity by modulating hemoproteins involved in fine-tuning circadian rhythms. These findings offer mechanistic insights into clinical and pre-clinical observations and pave the way for further exploration of the role of circadian rhythms in T cell biology.

d. Proteasome activity determines the therapeutic efficacy of CD19 CAR-T Cells: Our study also highlights the impact of intrinsic proteasome activity in T cells on the quality of CD19 CAR-T cells in human settings. These findings offer novel insights into optimizing T cell-based immunotherapies and hold significant translational potential for improving therapeutic efficacy.

3. Logical Linkage of Pathways and Presentation

We acknowledge the reviewer’ s concern regarding the logical linkage of pathways in our manuscript. To address this, we have thoroughly revised and restructured the manuscript to improve coherence and readability. We have expanded the results and discussion sections to provide clearer explanations, ensuring the narrative is logically connected. Additionally, we have conducted professional editing to ensure grammatical accuracy in the revised manuscript.

We hope that the reviewer finds the revised manuscript addresses the concerns. We believe that our study provides novel and impactful insights into how impaired mitochondrial dynamics and mitochondria health drive T cell exhaustion, filling critical gaps in the field and broaden the horizon on immunometabolic regulations in T cell differentiation. Furthermore, the findings from human CD19 CAR T-cell therapy offer proof-of-concept evidence demonstrating how the mechanisms we uncover can be leveraged to enhance therapeutic interventions. We are confident that the revised manuscript now meets the standards of clarity, coherence, and novelty expected by Nature.

Major concerns:

1. The major scientific issue with the manuscript is that the authors do not convincingly show that heme drives exhaustion (the main conclusion of the paper). In Figure 3 the authors demonstrate that hemin, in vitro, is sufficient to reduce cytokine production but also reduces proliferation (Supp Fig 3e), making it difficult to interpret if the lower cytokine production is due to lower proliferation/TCR stimulation (hemin potentially decreases CD44 but this is not shown) or increased cell death (viability is not shown). Further, while decreased functionality is the true readout of T cell exhaustion, the authors don’t show any other exhaustion associated markers by flow cytometry in their in vitro experiments (Fig 3a-f). Their in vivo transfer experiments (Fig 3i-m) only show modest affects, with the largest being cytokine production and cell number/expansion. These data could also be interpreted as hemin decreasing the proliferation and cytokine production of T cells independent of affecting exhaustion.

Reponses: We thank the reviewer for the comments. To address the concerns raised by the reviewer, we conducted several new experiments and we believe that our original results and the new results provide clarifications to support our conclusion.

a. In response to the reviewer’s concerns, we conducted a new set of experiments to assess whether hemin treatment affects TCR activation using P14 mice expressing the *Nur77-GFP* reporter, a well-established system for measuring TCR signal strength [2].

We primed naïve P14 cells from P14 mice expressing *Nur77-GFP* reporter and then repetitively stimulated them with an anti-CD3 antibody in the presence of a control vehicle or escalating doses of hemin. Our result showed that hemin treatment enhanced Nurr77-GFP expression, indicating that hemin increased TCR signaling rather than suppressed it (**revised Extended Data Figure 2k-l**). Moreover, we incorporated new data showing that hemin treatment upregulated exhaustion markers, including PD1 and TIM3 (**revised Figure 2h-i**), while concurrently suppressing CD44 expression- a characteristic feature of more exhausted T cells [3]) (**revised Extended Data Figure 2m-n**).

b. We also confirmed that hemin treatment reduced cell viability in a dose-dependent manner (**Reviewer Figure 5**). Therefore, we reason that the observed reduction in cell number under hemin treatment is an integrated outcome of both reduced cell viability and impaired proliferation. However, for assays measuring cytokine production, we re-stimulated T cells with gp33 peptide and only analyzed cytokine production in live cells. This experimental design ensures that our findings are independent of cell death and instead reflect intrinsic functional changes in T cells. Moreover, given that hemin treatment enhances TCR signaling, it is unlikely that the observed phenotype resulted from declined TCR activation.

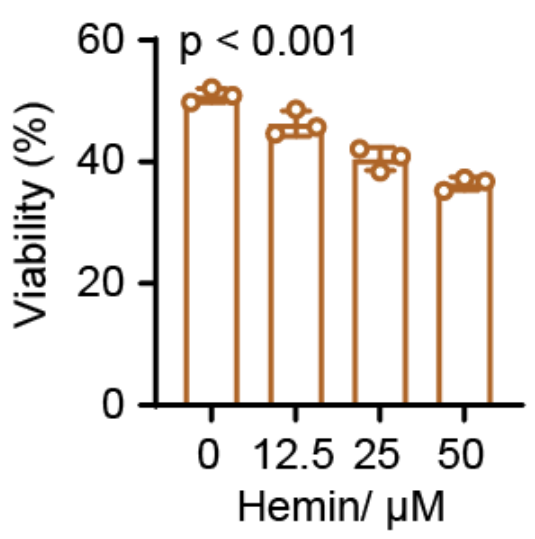

Reviewer Figure 5. The quantitative results for Zomib Aqua staining in ex vivo cultured murine $CD8^+$ T cells treated with indicated dose of Hemin for 48 hours in the repetitive s t i m u

c. As mentioned by the reviewer, decreased functionality is a true readout of T cell exhaustion. In our original manuscript, we transferred equal number of viable cells derived from control- or hemin-treated groups into naïve mice, followed by infection with the LCMV Amstrong clone. This experimental design enabled us to determine the functional decline in terminally exhausted T cells, as indicated by reduced expansion, sustained PD-1 expression, declined TCF1 expression, and impaired cytokine production in the expanded population [4]. Importantly, in this assay, P14 cells were exposed to hemin treatment only before transfer, and we specifically measured PD-1 and TCF1 expression, as well as cytokine production, exclusively in the expanded live P14 cells. Thus, we argue that our findings are independent of cell death and reflect intrinsic functional changes in T cells.

d. In addition to flow cytometry analyses of hemin-treated cells and in vivo functional assessments upon re-challenge, we conducted RNA-seq to confirm that hemin promotes the expression of gene signatures associated with T cell exhaustion (**revised Figure 2n**). Moreover, we performed ATAC-seq and applied Taiji analysis to integratively assess transcriptional networks in P14 cells exposed to hemin. This analysis further demonstrated that hemin treatment induces the transition of transcriptional networks linked to terminally exhausted T cells (**revised Figure 2o**).

Taken together, by employing multiple complementary approaches, including FACS analysis of in vitro samples, in vivo functional assessments, RNA-seq, and integrative analysis of chromatin accessibility and gene expression), we have rigorously validated that increased freely available heme promotes T cell exhaustion. We hope the reviewer agrees that these results provide strong evidence to support our conclusion and our new data effectively addresses the reviewer's concerns.

2. In figure 3n, the authors demonstrate that hemin treatment, which increases intracellular heme, increases Bach2 transcriptional activity and decreases Blimp1 transcriptional activity. However, in figure 4b-c they show that hemin treatment lowers Bach2 expression and increases Blimp1 expression. While expression does not necessarily equal activity, these contradictory results are not explained and confound the conclusions of hemin treatment on T cell function/exhaustion.
Reponses: We thank the reviewer for raising these important points. To clarify, Figure 3n in the original manuscript (now **revised Figure 2o**) presents the Taiji analysis, which evaluates the activity of transcription regulators by integrating RNA-seq and ATAC-seq data. However, as noted, Taiji analysis does not distinguish between transcriptional activators and repressors. The result of Figure 2o indicates that target genes controlled by Bach2 are highly expressed, while those controlled by Blimp1 (encoded by *PRDM1*) are significantly suppressed in the hemin-treated group. Notably, Bach2 and Blimp1 are known "transcriptional repressor", and Bach2 can suppress Blimp1 expression [5]. So the result of Taiji analysis should be interpreted as hemin treatment abolishing Bach2-mediated transcriptional suppression while promoting Blim1-controlled transcriptional suppression. This analysis suggest that hemin treatment alleviates Bach2-mediated transcriptional repression while increasing Blimp1 expression and function. This observation in the Taiji analysis is consistent with our findings in the original Figure 4b-c (now **revised Figures 3b-c)**, where we demonstrated that hemin treatment suppresses Bach2 expression while promoting Blimp1 expression. We acknowledge that the description of these findings in the original manuscript may have been unclear and the lack of clarification that Bach2 and Blimp1 are transcriptional repressor, which could have led to confusion. To address this, we have revised the description of the results to improve clarity. We hope the reviewer find our revised manuscript resolves any ambiguity and provides a clear rationale for the consistency of our findings.

3. A lot of genomics data was used to characterize different Tex populations that arise from the Bach2 heme binding mutant. However, there was no functional data (<u>cytokine production, tumor growth</u>) that demonstrated any biological significance of the increased Tpex population that the Bach2 mutant T cells brought about. In line with this the authors even admit that "…overexpressing a heme-binding insensitive Bach2 mutant in these T cells failed to achieve these beneficial outcomes (Figure 4d, 4h and Extended Data Figure 4o)". These data and this statement make it clear that heme binding by Bach2 is not a driving mechanism for how heme impacts T cell function/exhaustion. These data make it unclear why Bach2 was pursued as a mechanism in the first place.
Reponses: We thank the reviewer for the comments regarding to Bach2 mutant. First, we would like to clarify that our primary focus in the original manuscript was to assess the impact of

Bach2 mutant through cellular immunology assays, including changes in Blimp1 expression, tumor infiltration, and the differentiation status of TILs expressing either wild type or mutant form of Bach2. Additionally, we performed scRNA-seq to determine if the expression of Bach2 mutant can alter the frequency of cell subsets among TILs as we observed based on cellular assays. This represents the only genomic dataset we collected in relation to the Bach2 mutant.

Moreover, previous studies have demonstrated that overexpression of Bach2 promotes stem-like features but suppresses effector functions in T cells since Bach can hamper the expression of genes controlling effector functions and differentiation [6,7]. In fact, we did test cytokine production and anti-tumor responses in T cells expressing Bach2 mutant. Consistent with these findings, our results showed that T cells overexpressing Bach2^{MUT} exhibited a greater reduction in effector molecule production compared to T cells expressing wild type Bach2 (**Reviewer Figure 6**). Furthermore, CAR T cells overexpressing either Bach2^{WT} or Bach2^{MUT} did not enhance anti-tumor responses (**Reviewer Figure 7**), and CAR T cells expressing Bach2^{MUT} tended to show reduced control of tumor growth compared to those expressing Bach2^{WT}. These findings are consistent with other previous reports. These results are also in line with our conclusion that heme binds to Bach2, promoting a decline in stemness and driving terminal differentiation in T cells. However, we have to acknowledge that enhancing T cell stemness alone does not directly equate to improved anti-tumor responses. As highlighted in recent studies, effective CD8^{+} T cells must balance stemness with the capacity to differentiate into effector T cells to achieve optimal anti-tumor activity [8]. We have incorporated this discussion into the revised manuscript for clarification. (see below) We hope this explanation addresses the reviewer' s concerns and provides clarity regarding our findings and their implications.

Revised paragraph in the revised manuscript: "However, Bach2^{MUT}-overexpressing P14 T cells failed to enhance cytokine secretion and Bach2^{MUT} overexpression in anti-Her2 CAR-T cells did not improve survival (data not shown), suggesting that Bach2^{MUT} maintains CD8^{+} T cells in a stem-like state at the cost of effector function. This aligns with a recent report showing that Bach2 overexpression blocks the acquisition of cytotoxic and effector function in T cells [7]. These findings reinforce the idea that T cell stemness alone is insufficient for enhanced anti-tumor responses. Effective CD8^{+} T cells must balance stemness and differentiation into effector cells to optimize tumor control [8]. "

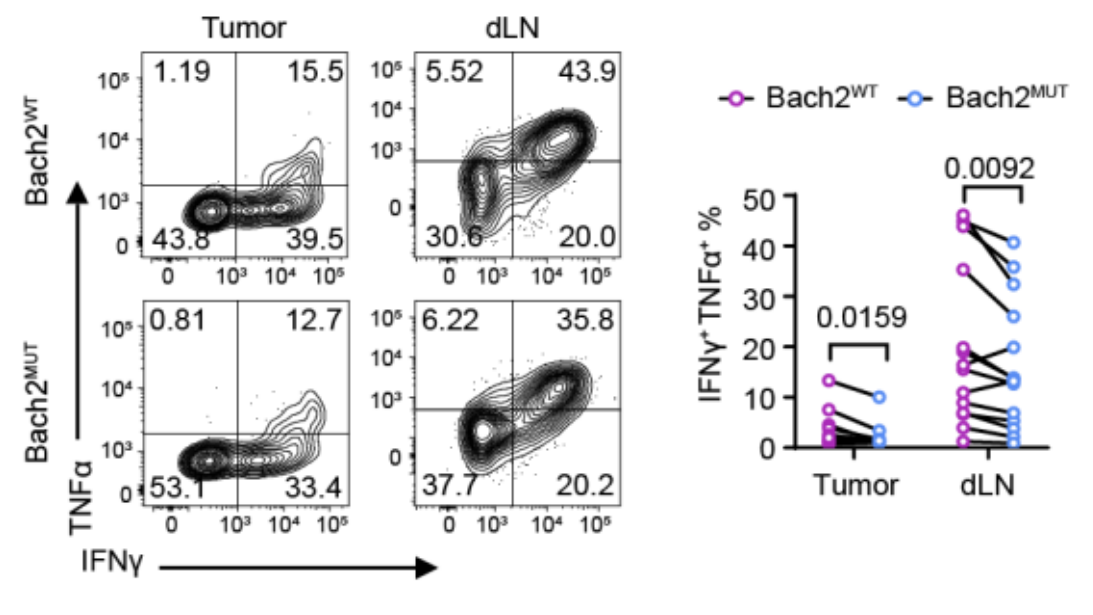

Reviewer Figure 6. Bach2^{WT} and Bach2^{MUT} P14 T cell with different congenic markers were co-transferred into YUMM1.7-gp33 tumor-bearing mice. (Left panel) The representative flow cytometry plots and (right panel) the quantitative results for TNFα and IFNγ stain

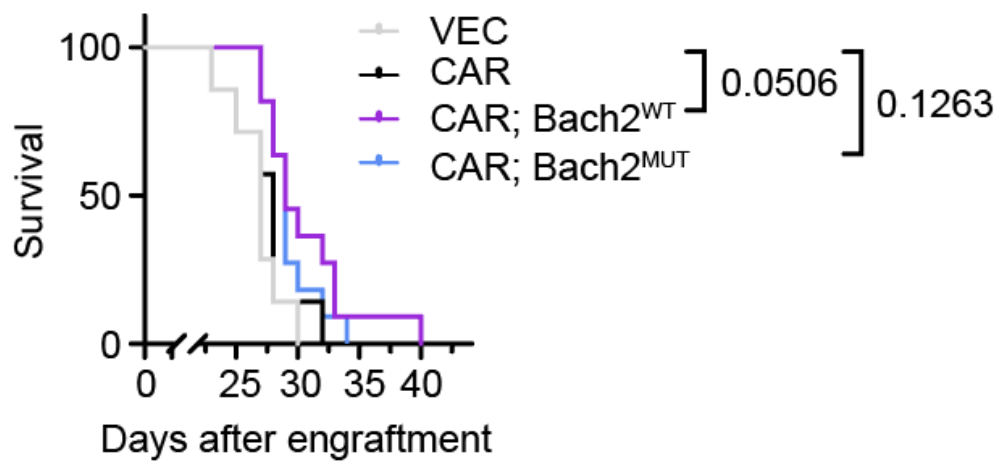

Reviewer Figure 7. Vector control, Anti-Her2 CAR, Anti-Her2 CAR with $Bach2^{WT}$ or Anti-Her2 CAR CAR with $Bach2^{MUT}$ were transduced into $CD8^+$ T cell, respectively. The generated VEC-T, CAR-T, CAR;$Bach2^{WT}$-T or CAR;$Bach2^{MUT}$-T cell were transferred into YUMM1.7-Her2 tumor-bearing mice. Kaplan-Meier survival curves show the survival probability (%) of the indicated groups of mice. The mice were recorded as deceased when the tumor size exceeds 800 mm3. Data shown are cumulative results of three independent experiments. *P < 0.05 is determine by log-rank test.

Referee #3 (Remarks to the Author):

In this manuscript, Xu and colleagues identify a proteasome gene signature in terminally exhausted TIL T cells, and show that pre-treatment of CAR T cells with the proteasome inhibitor bortezomib leads to an increase in anti-tumoral effect. This is mechanistically explained by the release of heme from mitochondrial proteins degraded via the proteasome, leading to harmful effects on T cell function. Further, they show that this is mediated via by binding of heme to Bach2, and that knockout of the nuclear heme importer Pgrmc2 improves anti-tumoral response. Finally, they identify the circadian clock gene Bmal1 as a heme target, and show that its deletion in T cells also improves T cell functionality.

The importance of the proteasome in $CD8^+$ T cell function has been shown previously (e.g PMID 28846070, PMID 21497118 - the former should be cited as a minimum), but not in the context of anti-tumoral immunity, and the translational potential of this work is therefore very high.

Reponses: We thank the reviewer for the valuable suggestion and extremely positive feedback about out study. We apologize for missing the citations during the editing process. We have now discussed the role of the proteasome in $CD8^+$ T cell function in the discussion section and have cited the suggested study (PMID 28846070) as recommended in the revised manuscript.

However, to fully substantiate this and the proposed underlying mechanisms, addition clarification is needed.

Generally, one of the issues of the manuscript is the extensive use of normalization (undermining the understanding of interexperimental variation), and paired t tests on non-normally distributed data, or non-paired data.

Reponses: We thank the reviewer for highlighting these important points. Regarding the extensive use of normalization, we acknowledge the concern about potentially undermining the understanding of inter-experimental variation. However, we would like to explain that several results presented are cumulative data from several independent experiments. For certain assays, such as the heme assay and FACS analyses of Bach2 and Blimp1 expression, the variation between independent experiments was too large to combine the results without normalization. To address this, we have provided additional explanations in the Methods section to clarify the

rationale for normalization and its application to ensure comparability across experiments. Furthermore, we now present unnormalized data for key experiments in the revised manuscript to enhance transparency regarding inter-experimental variability. As for the use of paired t-tests on non-normally distributed or non-paired data, we have thoroughly re-evaluated our statistical analyses to ensure the use of appropriate tests. For data that are not normally distributed, we have applied non-parametric tests (e.g., Mann-Whitney U test or Wilcoxon signed-rank test). Similarly, for non-paired data, we used unpaired t-tests or equivalent non-parametric tests where appropriate. These revisions have been detailed in the revised Methods section, and the figure legends have been updated accordingly. We hope these changes address the reviewer's concerns and enhance the rigor and transparency of our analyses.

Related to Figure 1.

A-B. What is the basis of the proteosome score? Is this from an existing database e.g. GO/Biocarta, or was it generated by the authors? Is there overlap with this list and other cluster defining genes? The gene list needs to be given.

Reponses: We appreciate the reviewer's question regarding the basis of the proteasome score. The proteasome score was calculated using genes annotated in the KEGG proteasome pathway (hsa03050) based on the gene weighting approach previously published by our collaborator [9]. This list is publicly available and has been curated as part of the KEGG database. For clarity and reproducibility, we have mentioned it in the Material and Method section and have included the full gene list used for the proteasome score in the Extended Table 9 of the revised manuscript.

E. What time point are these single cell data from? Extended Data 1D mentions day 9 or day 21.

Reponses: We thank the reviewer for raising this question. The single-cell data were obtained from CAR-T cells sorted from the peripheral blood of patients with B cell acute lymphoblastic leukemia at two time points: Day 9 (Peak) and Day 21 (Late) post anti-CD19 CAR-T infusion. To avoid potential bias and cover all cells collected from this cohort, all sorted CAR-T cells from these time points were used for scRNA-seq analysis. To ensure clarity, we have included this information in the figure legend for the **revised Figure 6c** (previously Figure 1e).

G. The proportion analysis needs to be done in a more statistically rigorous way, for example using scCODA, Milo, or other computational technique.

Reponses: We appreciate the reviewer's suggestion to enhance the statistical rigor of our proportion analysis. Following this recommendation, we utilized Milo for the statistical analysis. The updated results are now presented in the **revised Figure 6e** in the revised manuscript.

K. This data can't be used to rule out changes in cell proliferation because there is no info on cell viability and maintenance. Proliferation data e.g. CFSE dilution, and the effect on viability needs to be shown.

Reponses: Thank you for this helpful suggestion. In response to this concern, we re-performed the whole human CD19 CAR T-cell experiment and incorporated additional experiments to

assess both T cell proliferation and viability after 0.1 nM Bortezomib treatment. Specifically, we used CellTrace Far Red dilution to evaluate proliferation and Zombie Aqua staining to determine viability of T cells at day 5 post CAR transduction. Our results showed that Bortezomib treatment does not impact proliferation and viability of CD19 CAR T-cells compared to those treated with the control vehicle (**revised Figure 6i－j**). We have included these findings in the revised manuscript to address the reviewer’ s concern.

M. The effect size here is very small and the difference is non-statistically significant.
Reponses: Thank you for this insightful observation. To address this concern, we re-performed the experiments and refined our approach by treating CAR-T cells with a lower dose of Bortezomib (0.1 nM) for only 24 hours on day 5 post-CAR transduction, rather than at the initiation of T cell activation. This adjustment minimizes potential effects of Bortezomib during the priming phase. With this refined experimental design, we focused on the expression of exhaustion markers, including PD-1, TIM-3 and CD39, in CD19 CAR T-cells following in vitro repetitive stimulation with target cells, as well as their in vivo anti-tumor responses (**revised Figure 6k-q**). Our new results continue to support our conclusion that Bortezomib pre-treatment during CAR T-cell production enhances resistance to exhaustion under repetitive stimulation and provides stronger therapeutic benefits.

N. There needs to be a statistical test comparing the CAR-T +/ bortezomib groups.
R
eponses: Thank you for the suggestion to include a direct statistical comparison between the
C
A
R
-
T

bortezomib groups. In the revised manuscript, we have performed and reported statistical analyses accordingly. Specifically, we pre-treated anti-CD19 CAR-T cells with a low dose of

O. The duration of bortezomib treatment has not been specified in the legends or methods. Many other critical details like injection regimen, CAR construct, or cell numbers have not been included in the legends. The effect of the CAR-T treatment is minimal compared to the control VEC-T condition. Is that expected?
Reponses: We appreciate the reviewer’ s comments and apologize for any confusion regarding our experimental details. In the revised manuscript, we have included the complete injection regimen and bortezomib treatment duration in the legend to **revised Figure 6o**, which clarifies the timing of each intervention. The construction of our anti-CD19 CAR-T is a second-generation design with a 4-1BB intracellular co-stimulatory domain, and its scFv recognition region is derived from our previously characterized HIB19 antibody clone [10,11]. We have also specified the CAR-T cell numbers and other procedural details in the revised figure legend to ensure full transparency. Regarding the concern about the beneficial effects of CAR-T cells, our new analyses demonstrated that CAR-T treatment significantly prolongs the survival of leukemia-bearing mice compared to the control condition (**revised Figure 6p-q**).
0
.
1

n
g
/

Related to Figure 2.

A. The toxicity and non-specific effect of Mitotracker dyes on mitochondrial function and dynamics have neither been critically tested nor acknowledged as a confounder or study limitation. Particularly with MTDR FM such effects have been reported in the field (PMID 11164242).

Reponses: Thank you for your valuable comments. In our previous study (Y-R. Yu et al., 2021, Nature Immunology), we utilized MDR and MG staining to distinguish MDR/MG^{high} and MDR/MG^{low} populations from tumor-infiltrating lymphocytes (TILs) and ex vivo-induced T cells with dysfunctional mitochondria. These populations were subsequently sorted, and their mitochondrial ultrastructure was examined using electron microscopy. Our data demonstrated that MDR/MG^{low} T cells exhibited disorganized cristae morphology, supporting the notion that this population represents cells with mitochondrial dysfunction. Moreover, we also applied inhibitors to assess how these staining can distinguish cellular defects in mitochondrial health in the study published in 2021. Furthermore, we confirmed the terminally exhausted phenotype of MDR/MG^{low} T cells through a rechallenge assay.

In the current study, we have taken additional steps to ensure the robustness of our findings. Beyond using MDR and MG staining to identify terminally exhausted T cells, we have incorporated well-established exhaustion markers, including TCF1, TIM3, PD1, and CD39 (as now highlighted in the revised manuscript). These markers further substantiate our conclusion that mitochondrial protein degradation leads to increased RH levels, which correlate with the severity of T cell exhaustion. We appreciate the opportunity to clarify these points and hope this addresses the reviewer's concerns.

C-D. It is unclear why these proteomics experiments were performed in the presence of CHX, textual clarification and justification are needed. Using CHX as a main condition in the comparisons might introduce mitochondrial bias if cells have elevated rates of active mitochondrial translation. Without the cytoplasmic protein input into the mitochondrial proteome, an unfolded protein response in the mitochondria may be activated and the magnitude of this activation might differ between cell types tested, introducing bias. So, the authors should compare mitochondrial translation rates between depolarised vs healthy cell populations. Ideally, they should also repeat the proteomics experiments with a condition containing mitochondrial translation inhibitor like chloramphenicol, or using a SILAC based methodology.

Reponses: We thank the reviewer for their detailed and thoughtful comments regarding the use of CHX in our experiments and the potential of mitochondrial bias. The rationale for applying CHX in our experimental design was to block general protein translation and assess the stability of pre-existing proteins between MDR/MG^{low} and MDR/MG^{high} T cells. As the reviewer mentioned, the different mitochondrial translation rate may be a confounding factor of our experiment. However, only 13 mitochondria-encoded proteins are translated by the mitochondrial translation machinery (Nature. 1981 Apr 9;290(5806):457-65), including MT-CO1, MT-CO2, MT-CO3, MT-ATP6, MT-ATP8, MT-ND1, MT-ND2, MT-ND3, MT-ND4, MT-ND5, MT-ND6, MT-ND4L, and MT-CYB. Among these, we observed a significant difference only in MT-CO2 levels between the MDR/MG^{low} and MDR/MG^{high} groups in the steady state,

while the other mitochondria-encoded proteins did not exhibit notable variations. Given this observation, we believe that differential mitochondrial translation is unlikely to be a major contributing factor to the differences between MDR/MG^{low} and MDR/MG^{high} T cells. While we did not perform these additional experiments, we believe the current results derived from different approaches, including proteomics, mito-degron reporter, and release of regulatory heme, provide sufficient evidence to support our conclusions. We hope this explanation adequately addresses the reviewer' s concerns and are happy to provide additional information if required.

F-G. The legend indicates that the authors subjected cells to a combination of oligmycin A, antimycin A, Mdivi-1 and CHX treatment. Whereas, on the actual figure only CHX is mentioned and in the text describing these findings, none of these inhibitors were mentioned or acknowledged. Why were they used? This is an extremely potent cocktail and may have a major effect on the outcome of the experiment. Also, the split scale rather exaggerates the effect size, which is modest.

Reponses: Thank you for raising this important point. We sincerely apologize for not adequately explaining the rationale behind the use of oligomycin A, antimycin A, and Mdivi-1 in the original submission. These treatments were employed to induce mitochondrial dysfunction, as established in our previous publication (Yu et. al. Nature Immunology 2021) [12]. We have clarified this in the revised manuscript.

Regarding the use of a split scale, we appreciate your feedback on the potential for exaggerating the effect size. To address this concern, we have revised the calculation method. Instead of using a division operation, we now use a subtraction operation to present the results. This adjustment also highlights the pronounced difference between mitochondrial protein degradation and cytosolic protein degradation in MDR/MG^{high} and MDR/MG^{low} T cells. These updated results are shown in the **revised Figure 1i-j**. In addition, we also applied this approach in both murine and human CD8+ T cells with different exhaustion severity. Based on exhaustion severity, we also confirmed that terminally exhausted T cells preferentially degrade mito-degron reporter compared to progenitor exhausted T cells (**revised Figure 1k-l**).

H-I. Paired t tests are inappropriate here as the data are not paired (it would seem, assuming that each subset was separately repetitively stimulated). Further, the data has been normalized so it is no longer normally distributed. Were inhibitors (e.g. oligomycin etc) used in I?

Reponses: Thanks for the thoughtful question. To clarify, for original Figure 2h (**revised Figure 2a**), T cells were treated with oligomycin A, antimycin A, and Mdivi-1, followed by staining with MDR and MG. Flow cytometry analysis revealed two distinct populations: MDR/MG^{high} and MDR/MG^{low} T cells. These populations were subsequently sorted from each sample and subjected to the heme assay. We examined the regulatory heme levels in both MDR/MG^{high} and MDR/MG^{low} T cells from the same mouse as a paired comparison. For Figure 2i (**revised Figure 2b**), human T cells expressing NY-ESO-1-specific TCR were repetitively stimulated with tumor cells expressing the NY-ESO-1 antigen. This process also resulted in MDR/MG^{high} and MDR/MG^{low} populations, which were sorted for further heme assay. We examined the regulatory heme levels in both MDR/MG^{high} and MDR/MG^{low} T cells from the same donor as a paired comparison. Since both datasets were generated from the same mouse

or human donor and involved sorting populations within each sample, we used paired analysis to confirm the increased of RH is a consistent outcome. In response to your comment regarding statistical tests, we have made revisions to ensure appropriate analysis. For Figure 2h (now revised as **Figure 2a**), we replaced the paired t-test with the Wilcoxon test to account for the normalized data. For Figure 2i (now revised as **Figure 2b**), we retained the paired t-test.

Lastly, regarding the experimental setup for Figure 2i, no inhibitors (e.g., oligomycin A, antimycin A, or Mdivi-1) were used. The MDR/MG ^{low} population in this experiment was induced solely by repetitive stimulation with tumor cells expressing the NY-ESO-1 antigen. This process also resulted in MDR/MG^{high} and MDR/MG^{low} populations upon multiple round of re-stimulation (**Reviewer Figure 8**). Overall, we generated T cells accumulating depolarized mitochondria with three methods for assessing RH levels and draw our conclusion. We appreciate your feedback and believe these updates and clarifications address your concerns. Moreover, we hope you also agree that we took a high standard to make our conclusion by using different approaches.

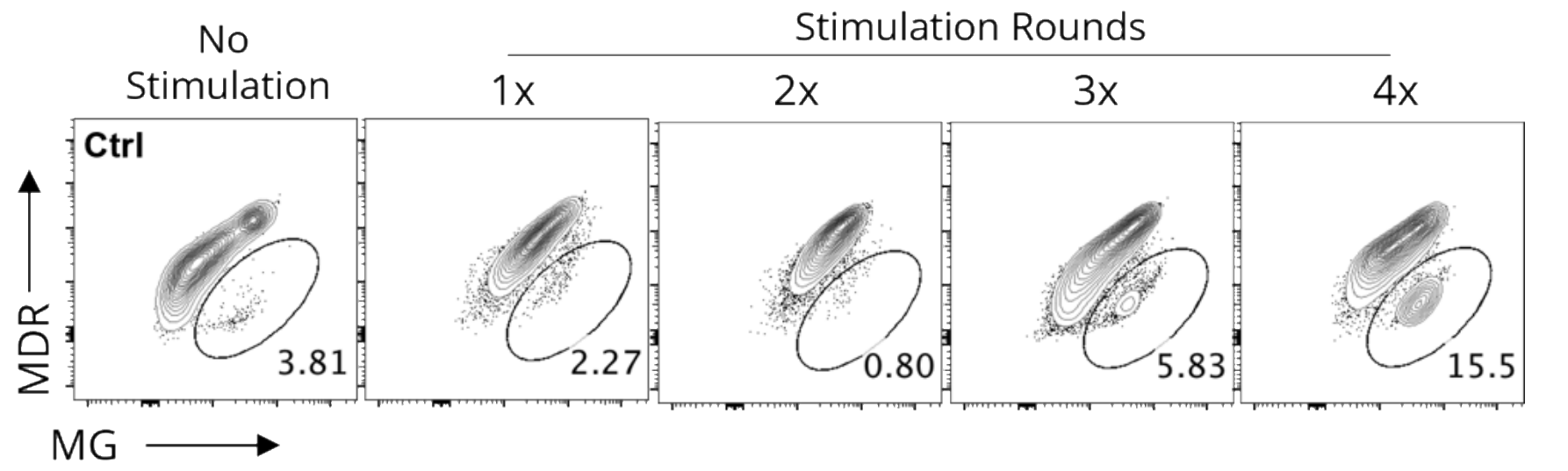

Reviewer Figure 8. The staining of MDR and MG in human CD8+ T cells expressing TCR against NY-ESO-1 under repetitive TCR stimulation.

K. What is the P value signifying? Regardless, the biggest issue is that the decrease in relative RH amount following MG132 treatment occurs at comparable rates between cell types, contradicting earlier findings suggesting MGlow cells have higher rates of proteasomal liberation of RH from mitochondria.

Reponses: Thank you for your valuable comments. We performed a Simple Linear Regression analysis on this data, and the p-value indicates that the slope is significantly non-zero. The statistical analysis revealed that the slope for the MDR/MG^{low} population is -24.1, whereas the slope for the MDR/MG^{high} population is -18.21. These results demonstrate that the MDR/MG^{low} population exhibits a higher rate of decrease in relative RH concentration following MG132 treatment. We sincerely apologize for not including the slope information in the original submission. We have now added this explanation and the corresponding statistical analysis to the revised manuscript (revised **Extended Data Figure 2c**). Additionally, we would like to clarify that both MDR/MG^{high} and MDR/MG^{low} population can have hemoprotein turnover originating from cytosolic proteins. However, the turnover amount is significantly lower compared to the mitochondrial protein degradation. We appreciate your careful review and hope this clarification, along with the updates to the manuscript, addresses your concerns.

Related to Figure 3.

Extended Data Fig. 3B - paired t test used inappropriately

Reponses: Thank you for pointing this out. The results in original Extended Data Figure 3b (revised **Extended Data Figure 3d**) represent the RH concentration of tumor interstitial fluid (TIF) and serum from the same tumor-bearing mouse. Thus, the nature of this data is indeed paired. However, since the data were normalized, we have replaced the paired t-test with the Wilcoxon test, which is more appropriate for normalized data. We have updated the statistical analysis method and revised the figure legend to reflect this change in the revised manuscript. We appreciate your feedback and have made the necessary adjustments in the revised manuscript.

The statement one line 227 that 'these findings indicate that the degradation of mitochondrial proteins increase RH levels, which correlates with the severity of T cell exhaustion' has not been substantiated.

Reponses: We appreciate the reviewer's thoughtful comments. In the revised manuscript, we have expanded our experiments to substantiate this statement. Specifically, we generated human T_{PEx} (progenitor exhausted T cells) and T_{TEx} (terminally exhausted T cells) and performed proteomic analysis, which revealed that T_{TEx} exhibits higher proteasome activity compared to T_{Pex} (**revised Figure 1c**). This finding was further confirmed using a proteasome activity probe (**revised Figure 1d-e**). Moreover, we conducted new experiments to determine regulatory heme levels in those human T cell subsets and confirmed that T_{TEx} have higher RH levels compared to T_{Pex} (**revised Figure 2e**). These new results are consistent with our findings that murine T_{TEx} have higher RH levels compared to T_{Pex} (**revised Figure 2d**).

To investigate mitochondrial protein degradation, we employed both the mitochondrial protein degradation reporter (mito-degron) and the cytosolic protein degradation reporter (cyto-degron) in a murine tumor model (**revised Figure 1k**) and a human ex vivo exhaustion model (**revised Figure 1l**). These results demonstrated that T_{TEx} degrades more mitochondrial reporter compared to T_{PEX}. Taken together, we believe the revised manuscript provides robust evidence that mitochondrial protein degradation increases RH levels, correlating with the severity of T cell exhaustion. We hope this explanation adequately addresses your concerns.

Related to Figure 4.

C. This experiment needs to be performed on Bach2 mutant cells to see if the increase in Blimp1 is prevented.

Reponses: Thanks for the advice. We transduced the $Bach2^{WT}$ and $Bach2^{MUT}$ into P14 cell from $Bach2^{CKO}$ mice, and induced the exhaustion of T cell by using αCD3 antibody restimulation with or without hemin treatment for 48 hours. We then assessed Blimp1 expression by flow cytometry. Our result confirmed that hemin induces Blimp1 expression in P14 cells overexpressing wild type Bach2 ($Bach2^{WT}$), while failing to upregulate Blimp1 expression in P14 cells overexpressing mutant form of Bach2 ($Bach2^{MUT}$). These results have been include in the revised manuscript (**revised Figure 3c** and **Extended Data Figure 3l**).

Extended Data Fig. 4A-D. Inhibitors were used for unjustified reasons.

Reponses: Thank you for raising this point. We sincerely apologize for not adequately explaining the rationale behind the use of oligomycin A, antimycin A, and Mdivi-1 in the

original submission. These treatments were employed to induce mitochondrial dysfunction, as established in our previous publication (Yu et. al. Nature Immunology 2021) [12]. We have clarified this in the revised manuscript (**revised Extended Data Figure 3a-d**) as discussed above.

Related to Figure 5.

Extended Data Fig. 5c-j. Again the statistical test used here is appropriate as these data are neither paired nor normally distributed (e and g).

Reponses: Thank you for your insightful comments. The results in original Extended Data Fig. 5c, 5e, and 5g represent the percentage of IFNγ/TNFα-producing TILs (c), as well as the expression levels of Bach2 (e) and Blimp1 (g) in P14 T cells expressing either control gRNA or *Pgrmc2*-targeting gRNA, isolated from the same tumor in mice that received co-transfer of both P14 cells. Since we performed co-transfer in each mouse and directly compared control gRNA-expressing and *Pgrmc2*-targeting gRNA-expressing P14 cells isolated from the same tumors, these results represent paired comparisons. However, as the data were normalized, we have replaced the paired t-test with the Wilcoxon test, which is more appropriate for normalized data. We have updated the statistical analysis method accordingly and revised the figure legends to reflect this change.

Related to Figure 6.

The use of paired t tests is not appropriate as the data are not paired, and sometimes also not normally distributed. Why are points joined?

Reponses: Thank you for your thoughtful comments. The experiments presented in the results of original Figure 6 (**revised Figure 5)** were co-transfer experiments as described in the previous point. Since we performed co-transfer in each mouse and directly compared control gRNA-expressing and *Nr1d1/2*-targeting gRNA-expressing P14 cells isolated from the same tumors, these results represent paired comparisons. We also incorporated new results regarding to Bmal1-deficiency with similar designs in the revised manuscript. To illustrate the paired nature of the data and experiemtns, we joined the points corresponding to the same tumor or mouse. Additionally, as the data were not normalized, we believe the assumption of normal distribution holds, making the use of the paired t-test appropriate in this context.

Reference:

1 Schurich, A. *et al.* Distinct Metabolic Requirements of Exhausted and Functional Virus-Specific CD8 T Cells in the Same Host. *Cell Rep* **16**, 1243-1252, doi:10.1016/j.celrep.2016.06.078 (2016).

2 Moran, A. E. *et al.* T cell receptor signal strength in Treg and iNKT cell development demonstrated by a novel fluorescent reporter mouse. *J Exp Med* **208**, 1279-1289, doi:10.1084/jem.20110308 (2011).

3 Tsui, C. *et al.* MYB orchestrates T cell exhaustion and response to checkpoint inhibition. *Nature* **609**, 354-360, doi:10.1038/s41586-022-05105-1 (2022).

4 Alfei, F. *et al.* TOX reinforces the phenotype and longevity of exhausted T cells in

chronic viral infection. *Nature* **571**, 265-269, doi:10.1038/s41586-019-1326-9 (2019).

5 Muto, A. *et al.* Bach2 represses plasma cell gene regulatory network in B cells to promote antibody class switch. *EMBO J* **29**, 4048-4061, doi:10.1038/emboj.2010.257 (2010).

6 Yao, C. *et al.* BACH2 enforces the transcriptional and epigenetic programs of stem-like CD8(+) T cells. *Nat Immunol* **22**, 370-380, doi:10.1038/s41590-021-00868-7 (2021).

7 Barton, P. R. *et al.* Super-killer CTLs are generated by single gene deletion of Bach2. *Eur J Immunol* **52**, 1776-1788, doi:10.1002/eji.202249797 (2022).

8 Zhou, P. *et al.* Single-cell CRISPR screens in vivo map T cell fate regulomes in cancer. *Nature* **624**, 154-163, doi:10.1038/s41586-023-06733-x (2023).

9 Xiao, Z., Dai, Z. & Locasale, J. W. Metabolic landscape of the tumor microenvironment at single cell resolution. *Nat Commun* **10**, 3763, doi:10.1038/s41467-019-11738-0 (2019).

10 Gu, R. *et al.* Efficacy and safety of CD19 CAR T constructed with a new anti-CD19 chimeric antigen receptor in relapsed or refractory acute lymphoblastic leukemia. *J Hematol Oncol* **13**, 122, doi:10.1186/s13045-020-00953-8 (2020).

11 An, N. *et al.* Construction of a new anti-CD19 chimeric antigen receptor and the anti-leukemia function study of the transduced T cells. *Oncotarget* **7**, 10638-10649, doi:10.18632/oncotarget.7079 (2016).

12 Yu, Y. R. *et al.* Disturbed mitochondrial dynamics in CD8(+) TILs reinforce T cell exhaustion. *Nat Immunol* **21**, 1540-1551, doi:10.1038/s41590-020-0793-3 (2020).

Response (Xu et al.)
We would like to thank the reviewers for the constructive and insightful comments to our manuscript. Based on the comments and critiques, we propose some experiments and attempt to answer these comments point by point in the letter. We think that the constructive comments raised by the reviewers indeed strengthen the depth of this review article. We hope these proposed experiments and replies in this letter can satisfy the reviewers' concerns.

Please find our "point-to-point" response (in blue) to the reviewer's comments below. We also highlighted modifications in blue and marked the new figures with blue labeling in figure number in the revised manuscript.

Referee #3 (Remarks to the Author):
In this manuscript, Xu and colleagues identify a proteasome gene signature in terminally exhausted TIL T cells, and show that pre-treatment of CAR T cells with the proteasome inhibitor bortezomib leads to an increase in anti-tumoral effect. This is mechanistically explained by the release of heme from mitochondrial proteins degraded via the proteasome, leading to harmful effects on T cell function. Further, they show that this is mediated via by binding of heme to Bach2, and that knockout of the nuclear heme importer Pgrmc2 improves anti-tumoral response. Finally, they identify the circadian clock gene Bmal1 as a heme target, and show that its deletion in T cells also improves T cell functionality.

The importance of the proteasome in $CD8^+$ T cell function has been shown previously (e.g PMID 28846070, PMID 21497118 - the former should be cited as a minimum), but not in the context of anti-tumoral immunity, and the translational potential of this work is therefore very high.

Reponses: We thank the reviewer for the valuable suggestion and extremely positive feedback about out study. We have discussed the role of the proteasome in $CD8^+$ T cell function in the discussion section and have cited the suggested study (PMID 28846070) as recommended in the revised manuscript.

However, to fully substantiate this and the proposed underlying mechanisms, addition clarification is needed.

Generally, one of the issues of the manuscript is the extensive use of normalization (undermining the understanding of interexperimental variation), and paired t tests on non-normally distributed data, or non-paired data.
Reponses: We thank the reviewer for highlighting these important points. Regarding the extensive use of normalization, we acknowledge the concern about potentially undermining the

understanding of inter-experimental variation. However, several results presented are cumulative data from several independent experiments. For certain assays, such as the heme assay and FACS analyses of Bach2 and Blimp1 expression, the variation between independent experiments was too large to combine the results without normalization due to the nature of experimental procedures, including machine status and batch of Apo-HRP we used. To address this, we have provided additional explanations in the Methods section to clarify the rationale for normalization and its application to ensure comparability across experiments. Furthermore, we now present unnormalized data for key experiments in the revised manuscript to enhance transparency regarding inter-experimental variability. As for the use of paired t-tests on non-normally distributed or non-paired data, we have thoroughly re-evaluated our statistical analyses to ensure the use of appropriate tests. For data that are not normally distributed, we have applied non-parametric tests (e.g., Mann-Whitney U test or Wilcoxon signed-rank test). Similarly, for non-paired data, we used unpaired t-tests or equivalent non-parametric tests where appropriate. These revisions have been detailed in the revised Methods section, and the figure legends have been updated accordingly. We hope these changes address the reviewer's concerns and enhance the rigor and transparency of our analyses.

Whilst I appreciate the authors taking this point on, there are still many instances of incorrect statistical testing which have not been corrected. For example, in Fig 1k, 2b, Extended data fig 4. ANOVA and multiple t tests are used variably. There is no description of how multiple testing is corrected in either the figure legends or methods section. In most cases these issues will not affect the overall validity of the work, but may do in some cases.

Response: We thank the reviewer for this valuable observation. We have carefully re-examined all statistical analyses presented in the manuscript and have standardized the tests across comparable datasets. Specifically, one-way ANOVA followed by Tukey' s multiple-comparison test is now consistently applied for comparisons involving more than two groups, whereas unpaired two-tailed t-tests are used for pairwise analyses. The figure legends and the Methods section have been revised accordingly to clearly describe the statistical approaches and correction methods used for multiple testing. We have verified that these updates do not alter the overall conclusions of the study, but we agree that consistent and transparent reporting strengthens the rigor and reproducibility of our results.

G. The proportion analysis needs to be done in a more statistically rigorous way, for example using scCODA, Milo, or other computational technique.

Reponses: We appreciate the reviewer' s suggestion to enhance the statistical rigor of our proportion analysis. Following this recommendation, we utilized Milo for the statistical analysis. The updated results are now presented in the revised Figure 6e in the revised

manuscript.

Thank you. This analysis should also be performed on the single cell data in Fig 3j.

Response: We thank the reviewer for this insightful follow-up. For the single-cell dataset shown in original Figure 3j (**Revised Figure 4j**), cell type annotation was performed by mapping to a well-established reference atlas (ProjecTILs). Thus, cell identities were assigned based on their global transcriptional similarity to reference cell types rather than unsupervised clustering within our dataset. Applying MiloR would require redefining new clusters using a k-nearest-neighbor (KNN) approach, which could complicate the interpretation and continuity of the manuscript. However, following the reviewer's suggestion, we conducted a MiloR analysis for comparison. The results showed that Tpex cells increased, whereas Tex cells decreased in the Bach2^{MUT} group (**Reviewer Figure 1**), consistent with our original findings presented in **Figure 4k**. To maintain a clear logical flow and avoid introducing redundant cluster definitions, we have decided to retain the original analysis while referencing these confirmatory MiloR results in the revised text.

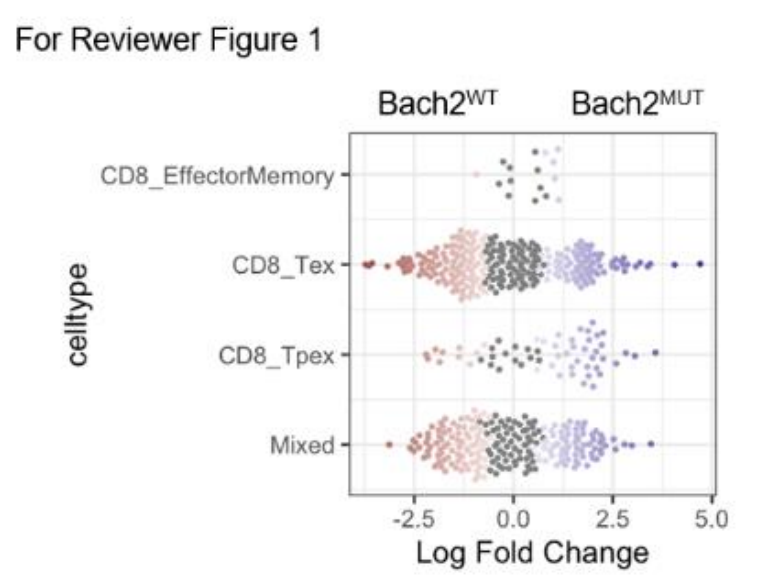

Reviewer Figure 1. Beeswarm plot of the log-transformed fold changes in cell abundance comparing CR versus RE. Differential abundance neighborhoods at a 5% FDR are highlighted with color.

K. This data can' t be used to rule out changes in cell proliferation because there is no info on cell viability and maintenance. Proliferation data e.g. CFSE dilution, and the effect on viability needs to be shown.

Reponses: Thank you for this helpful suggestion. In response to this concern, we re-performed the whole human CD19 CAR T-cell experiment and incorporated additional experiments to assess both T cell proliferation and viability after 0.1 nM Bortezomib treatment. Specifically, we used CellTrace Far Red dilution to evaluate proliferation and Zombie Aqua staining to determine viability of T cells at day 5 post CAR transduction. Our results showed that Bortezomib treatment does not impact proliferation and viability of CD19 CAR T-cells compared to those treated with the control vehicle (revised Figure 6i-j). We have included these findings in the revised manuscript to address the reviewer' s concern.

Thank you. In new Fig 1k-l, why are the same type of data presented differently? Why are

paired (incorrect) and unpaired t tests used for these similar panels?

Response: We have modified the type of data presented in Figure 1k (**Revised Figure 1e-f**) to match that of Figure 1l (**Revised Extended Figure 1h**) and changed the statistical analysis method to an unpaired t-test. The statistical method and corresponding figure legend have been updated in the revised manuscript.

Related to Figure 2.

A. The toxicity and non-specific effect of Mitotracker dyes on mitochondrial function and dynamics have neither been critically tested nor acknowledged as a confounder or study limitation. Particularly with MTDR FM such effects have been reported in the field (PMID 11164242).

Reponses: Thank you for your valuable comments. In our previous study (Y-R. Yu et al., 2021, Nature Immunology), we utilized MDR and MG staining to distinguish MDR/MGhigh and MDR/MGlow populations from tumor-infiltrating lymphocytes (TILs) and ex vivo-induced T cells with dysfunctional mitochondria. These populations were subsequently sorted, and their mitochondrial ultrastructure was examined using electron microscopy. Our data demonstrated that MDR/MGlow T cells exhibited disorganized cristae morphology, supporting the notion that this population represents cells with mitochondrial dysfunction. Moreover, we also applied inhibitors to assess how these staining can distinguish cellular defects in mitochondrial health in the study published in 2021. Furthermore, we confirmed the terminally exhausted phenotype of MDR/MGlow T cells through a rechallenge assay.

In the current study, we have taken additional steps to ensure the robustness of our findings. Beyond using MDR and MG staining to identify terminally exhausted T cells, we have incorporated well-established exhaustion markers, including TCF1, TIM3, PD1, and CD39 (as now highlighted in the revised manuscript). These markers further substantiate our conclusion that mitochondrial protein degradation leads to increased RH levels, which correlate with the severity of T cell exhaustion. We appreciate the opportunity to clarify these points and hope this addresses the reviewer’ s concerns.

Whilst I appreciate the authors response, it does not fully address this point, which should be stated as a limitation in the discussion.

Response: We appreciate the reviewer’s suggestion and this has been included in the discussion section in the revised manuscript.

C-D. It is unclear why these proteomics experiments were performed in the presence of CHX, textual clarification and justification are needed. Using CHX as a main condition in the comparisons might introduce mitochondrial bias if cells have elevated rates of active mitochondrial translation. Without the cytoplasmic protein input into the mitochondrial proteome, an unfolded protein response in the mitochondria may be activated and the magnitude of this activation might differ between cell types tested, introducing bias. So, the authors should compare mitochondrial translation rates between depolarised vs healthy cell populations. Ideally, they should also repeat the proteomics experiments with a condition containing mitochondrial translation inhibitor like chloramphenicol, or using a SILAC based methodology.
Reponses: We thank the reviewer for their detailed and thoughtful comments regarding the use of CHX in our experiments and the potential of mitochondrial bias. The rationale for applying CHX in our experimental design was to block general protein translation and assess the stability of pre-existing proteins between MDR/MGlow and MDR/MGhigh T cells. As the reviewer mentioned, the different mitochondrial translation rate may be a confounding factor of our experiment. However, only 13 mitochondria-encoded proteins are translated by the mitochondrial translation machinery (Nature. 1981 Apr 9;290(5806):457-65), including MT-CO1, MT-CO2, MT-CO3, MT-ATP6, MT-ATP8, MT-ND1, MT-ND2, MT-ND3, MT-ND4, MT-ND5, MT-ND6, MT-ND4L, and MT-CYB. Among these, we observed a significant difference only in MT-CO2 levels between the MDR/MGlow and MDR/MGhigh groups in the steady state, while the other mitochondria-encoded proteins did not exhibit notable variations. Given this observation, we believe that differential mitochondrial translation is unlikely to be a major contributing factor to the differences between MDR/MGlow and MDR/MGhigh T cells. While we did not perform these additional experiments, we believe the current results derived from different approaches, including proteomics, mito-degron reporter, and release of regulatory heme, provide sufficient evidence to support our conclusions. We hope this explanation adequately addresses the reviewer's concerns and are happy to provide additional information if required.

Thank you.

In new Fig 2d, plotting a regression line is not appropriate here and a Kruskal–Wallis test should be used instead to examine whether intergroup differences are statistically significant. Fig 2t would typically be analysed by 2-way ANOVA (as done in Fig 3d).

Response: We have updated the statistical analysis for Figure 2d (**Revised Figure 3b**) and Figure 2t (**Revised Figure 3o**) according to your suggestions. The statistical methods and

corresponding figure legends have been revised accordingly in the updated manuscript.

F-G. The legend indicates that the authors subjected cells to a combination of oligmycin A, antimycin A, Mdivi-1 and CHX treatment. Whereas, on the actual figure only CHX is mentioned and in the text describing these findings, none of these inhibitors were mentioned or acknowledged. Why were they used? This is an extremely potent cocktail and may have a major effect on the outcome of the experiment. Also, the split scale rather exaggerates the effect size, which is modest.

Reponses: Thank you for raising this important point. We sincerely apologize for not adequately explaining the rationale behind the use of oligomycin A, antimycin A, and Mdivi-1 in the original submission. These treatments were employed to induce mitochondrial dysfunction, as established in our previous publication (Yu et. al. Nature Immunology 2021) 12. We have clarified this in the revised manuscript.

Regarding the use of a split scale, we appreciate your feedback on the potential for exaggerating the effect size. To address this concern, we have revised the calculation method. Instead of using a division operation, we now use a subtraction operation to present the results. This adjustment also highlights the pronounced difference between mitochondrial protein degradation and cytosolic protein degradation in MDR/MGhigh and MDR/MGlow T cells. These updated results are shown in the revised Figure 1i-j. In addition, we also applied this approach in both murine and human CD8+ T cells with different exhaustion severity. Based on exhaustion severity, we also confirmed that terminally exhausted T cells preferentially degrade mito-degron reporter compared to progenitor exhausted T cells (revised Figure 1k-l).

Thanks - although the rationale for the cocktail needs to be mentioned in the main text.

Response: We appreciate the reviewer' s suggestion. The rationale for using the inhibitor cocktail has now been explicitly incorporated into the main text.

K. What is the P value signifying? Regardless, the biggest issue is that the decrease in relative RH amount following MG132 treatment occurs at comparable rates between cell types, contradicting earlier findings suggesting MGlow cells have higher rates of proteasomal liberation of RH from mitochondria.

Reponses: Thank you for your valuable comments. We performed a Simple Linear Regression analysis on this data, and the p-value indicates that the slope is significantly non-zero. The statistical analysis revealed that the slope for the MDR/MGlow population is -24.1, whereas the slope for the MDR/MGhigh population is -18.21. These results demonstrate that the MDR/MGlow population exhibits a higher rate of decrease in relative RH concentration

following MG132 treatment. We sincerely apologize for not including the slope information in the original submission. We have now added this explanation and the corresponding statistical analysis to the revised manuscript (revised Extended Data Figure 2c). Additionally, we would like to clarify that both MDR/MGhigh and MDR/MGlow population can have hemoprotein turnover originating from cytosolic proteins. However, the turnover amount is significantly lower compared to the mitochondrial protein degradation. We appreciate your careful review and hope this clarification, along with the updates to the manuscript, addresses your concerns.

The P value in the test is not comparing the two conditions (from what I can see). It would make more sense to do a 2-way ANOVA (as done elsewhere), as I do not see the specific reason a linear regression fit is done here, and not in the many other examples of dose-response experiments in the manuscript.

Response: We appreciate the reviewer' s point. Our primary purpose for applying linear regression was to quantify the degradation rate by extracting the slope parameter from MDR/MG^{high} and MDR/MG^{low} populations, as the slope directly reflects the rate of RH decline (i.e., a more negative slope indicates a faster degradation rate).

In response to the reviewer' s suggestion, we have now retained the linear regression analysis for calculating degradation rates, and additionally performed a two-way ANOVA to statistically compare the differences between the two groups across time points. These updated statistical results have been incorporated into the revised manuscript for clarity and completeness.

Related to Figure 3.

Extended Data Fig. 3B - paired t test used inappropriately

Reponses: Thank you for pointing this out. The results in original Extended Data Figure 3b (revised Extended Data Figure 3d) represent the RH concentration of tumor interstitial fluid (TIF) and serum from the same tumor-bearing mouse. Thus, the nature of this data is indeed paired. However, since the data were normalized, we have replaced the paired t-test with the Wilcoxon test, which is more appropriate for normalized data. We have updated the statistical analysis method and revised the figure legend to reflect this change in the revised manuscript. We appreciate your feedback and have made the necessary adjustments in the revised manuscript.

Although the measurements are from the same mouse, they are different variables that are not paired (e.g. not 'before-after' from the same sample), and so an unpaired statistical test should

be used e.g. Mann-Whitney U test.

Response: Thank you for this clarification. We have re-analyzed the results using the Mann-Whitney U test, which is appropriate for comparisons between two independent variables. The statistical method and corresponding figure legend have been updated in the revised manuscript.

In extended data figure 4 - all the statistical tests here are wrong (assuming Wilcoxon signed rank test referred to). None of the samples in here are paired since they come from separate individual mice.

Response: We appreciate the reviewer' s comment regarding the statistical tests. We would like to clarify that the datasets shown in Extended Data Fig. 4c, 4e, 4g, and 4j (**Revised Extended Data Fig. 5c, 5e, 5g, and 5k**) are indeed paired measurements, as they were derived from samples obtained from the same mice in co-transfer experiments. Thus, the assumptions of a paired design are met. We also note that the use of paired t-tests is standard for analyzing co-transfer experiments in which two cell populations are isolated from the same animal. For example, recent studies in *Nature* (Zhou et al., 2023; Fig. 3c) and *Cell* (Ngiow et al., 2024; Fig. 2H) have applied paired t-tests in analogous experimental designs (examples provided in **Reviewer Figure 2**). Based on both our experimental structure and established precedent in the literature, we are confident that the paired t-test is appropriate for these analyses.

For reviewer Figure 2

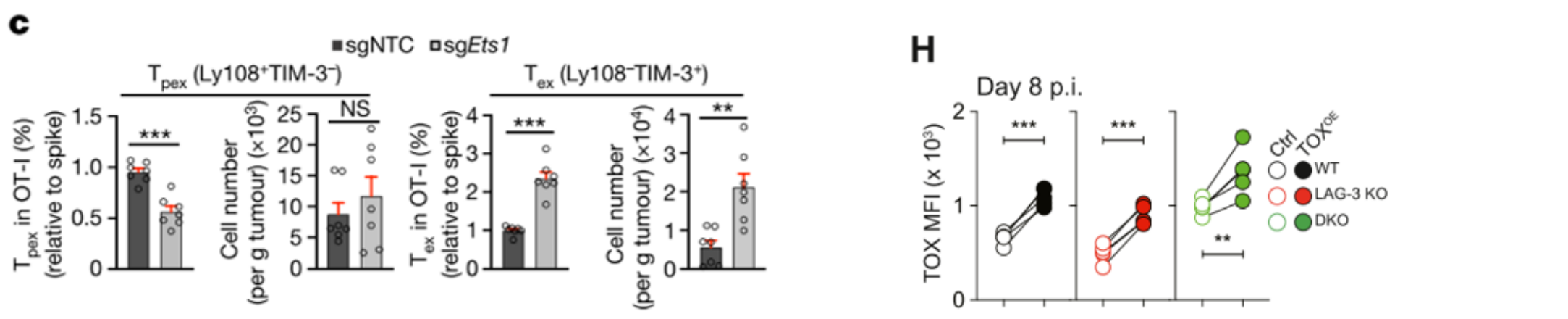

(**b**–**e**), two-tailed paired Student's *t*-test

P. Zhou, et al., Nature, 2023, Figure 3c

(H and I) *p* values calculated using two-tailed paired t test.

S. F. Ngiow, et al., Cell, 2024, Figure 2

Related to Figure 5.

Extended Data Fig. 5c-j. Again the statistical test used here is appropriate as these data are neither paired nor normally distributed (e and g).

Reponses: Thank you for your insightful comments. The results in original Extended Data Fig. 5c, 5e, and 5g represent the percentage of IFNg/TNFa-producing TILs (c), as well as the expression levels of Bach2 (e) and Blimp1 (g) in P14 T cells expressing either control gRNA or Pgrmc2-targeting gRNA, isolated from the same tumor in mice that received co-transfer of

both P14 cells. Since we performed co-transfer in each mouse and directly compared control gRNA-expressing and Pgrmc2-targeting gRNA-expressing P14 cells isolated from the same tumors, these results represent paired comparisons. However, as the data were normalized, we have replaced the paired t-test with the Wilcoxon test, which is more appropriate for normalized data. We have updated the statistical analysis method accordingly and revised the figure legends to reflect this change.

Thanks. Again, with relation to paired t tests, the assumption is that the control and Pgrmc-2 P14 T cells are generated from the same mouse, which I assume to be the case. Even then, it is stretching it to assume that the data can be analyzed in a pairwise fashion.

Response: We appreciate the reviewer' s comment regarding the statistical tests. We would like to clarify that the datasets shown in those data are indeed paired measurements, as they were derived from samples obtained from the same mice in co-transfer experiments. We have addressed this point as described above.

Referee #4 (Remarks to the Author):

This manuscript provides intriguing insights into how proteasome activity regulates T cell exhaustion, linking mitochondrial dysfunction and heme-mediated signaling with transcriptional reprogramming and immunotherapeutic failure. The study is ambitious in scope and addresses timely concepts in immunometabolism and T cell biology. However, significant mechanistic and explanatory gaps remain. My main concerns follow:

1) While the authors present compelling data that proteasome activity is upregulated in exhausted $CD8^+$ T cells and correlates with degradation of mitochondrial proteins, the mechanistic basis for this selectivity is not addressed. The authors do not provide biochemical, structural, or post-translational data to explain why mitochondrial targets- including hemoproteins- are specifically recognized by the proteasome. This omission leaves a key conceptual gap in the central model.

Response: We sincerely appreciate the reviewer's insightful feedback. We agree that understanding the mechanistic basis for the selectivity of mitochondrial protein degradation is critical. To address this, we conducted a series of experiments to investigate the underlying mechanisms governing this process as listed below.

a. To examine the selective degradation of mitochondrial proteins, we treated T_E, T_M, and T_{TEx} cells with MG132 to inhibit the proteasomal degradation of ubiquitinated proteins. Using anti-ubiquitin beads to enrich for ubiquitinated proteins, we performed

proteomics analysis (**Revised Figure 1g**). We observed that, compared to T_E cells, ubiquitinated proteins in T_M cells were predominantly cytoplasmic, whereas in T_{TEx} cells, they were predominantly mitochondrial (**Revised Figure 1h**). These observations indicate that exhausted T cells preferentially engage proteasome-mediated catabolic pathway, thereby directing the selective degradation of mitochondrial proteins.

b. We investigated the potential E3 ligases contributing to selective mitochondrial protein degradation by analyzing their expression across the proteomes of T_E, T_M, T_{PEx}, and T_{TEx} cells. We identified several upregulated E3 ligases in exhausted T cells, including CBL-B, which has previously been reported to regulate mitochondrial potential in tumor cells (Feng, D. et al., Int J Mol Sci, 2013). To test the contribution of CBL-B, we ablated CBL-B expression in human T cells harboring mCherry-degron reporter and observed that the degradation of mitochondrial mCherry-degron reporter is selectively rescued in CBL-B-deficient T_{TEx}, whereas cytoplasmic counterpart is not affected by the loss of CBL-B (**Revised Figures 1j-k**). This finding supports the idea that CBL-B regulates mitochondrial protein degradation in exhausted T cells.

c. In addition to human T cells with different exhaustion severity, our result also showed that murine TILs accumulating depolarized mitochondria expressed higher levels of CBL-B compared to TILs with superior mitochondrial activity (**Revised Figures 2l**). Similar to human T cells, genetic ablation of CBL-B selectively prevented the degradation of mitochondrial mCherry-degron reporter, but no effect on cytoplasmic mCherry-degron reporter, in T cells accumulating depolarized mitochondria (**Revised Figures 2m-n**). Moreover, CBL-B-deficiency significantly reduced regulatory heme (RH) levels in T cells accumulation depolarized mitochondria (MDR/MG^{low}), but had no impact in MDR/MG^{high} subsets (**Revised Figures 2o-p**).

Taken together, these results unveil that the expression of CBL-B orchestrate a selective proteasome-dependent program that targets mitochondrial proteins for degradation in exhausted T cells. This CBL-b-driven pathway emerge as a crucial determinant of the preferential engagement of proteasomal degradation of mitochondrial proteins during the establishment of T cell exhaustion.

2) The proposed pathway implies that dysfunctional mitochondria trigger proteasome engagement. However, the upstream signaling axis responsible for this upregulation is neither tested nor hypothesized. It is unclear whether mitochondrial depolarization itself initiates proteasome activation or whether this is a consequence of an independent exhaustion-associated program.

Response: We sincerely appreciate the reviewer for raising this point. In response to your question regarding the upstream signaling axis responsible for the upregulation of proteasome activity, we have expanded on the data in the revised manuscript.

a. As shown in **Revised Figure 2a-b**, our proteomics data reveal that the MDR/MG^{low} population exhibits significant enrichment in proteasome components, suggesting that mitochondrial dysfunction is linked to proteasome activation. To further investigate this, T cells were treated with Mdivi-1 (mitophagy inhibitor) and Oligomycin A plus Antimycin A (mROS inducer to drive damaged mitochondria) within 4 hours. Notably, these treatments directly disturbed mitochondrial function and promoted accumulation of depolarized mitochondria. We further confirmed that T cells accumulating depolarized mitochondria under this treatment preferentially degrade mitochondria component by using degron reporters (**Revised Figures 2e-f**). These results revealed that accumulation of dysfunctional mitochondria is sufficient to drive mitochondrial protein degradation,
b. In addition to degron reporter, we assessed ubiquitinated protein profile in both MDR/MG^{high} and MDR/MG^{low} T cells treated with MG132 (**Revised Figure 2j**). Our analyses revealed that MDR/MG^{low} T cells exhibited a marked enrichment of ubiquitinated proteins associated with the mitochondrial compartment (**Revised Figure 2k**). These findings further support the idea that mitochondrial dysfunction leads to preferential ubiquitination process that leads to subsequent degradation of mitochondrial proteins.
c. We further investigated which E3 ligase can be engaged to drive mitochondrial protein degradation in T cell accumulating depolarized mitochondria. The role of CBL-B E3 ligase in this process. We observed that MDR/MG^{low} T cells upregulated CBL-B E3 ligase and genetic ablation of CBL-B preferentially prevented degradation of mitochondrial degron reporter and reduced RH levels in MDR/MG^{low} T cells (**Revised Figure 2l-p**). These results further suggest that mitochondrial depolarization triggers a CBL-B-dependent, proteasome-mediated degradation of mitochondrial proteins.

Taken together, these findings suggest that mitochondrial depolarization triggers proteasome activation via CBLB-mediated ubiquitination of mitochondrial proteins, rather than this being a consequence of an independent exhaustion-associated program. We propose that CBL-B E3 ligase plays a key role in the signaling axis linking mitochondrial dysfunction to proteasomal engagement, promoting the degradation of specific mitochondrial proteins. We hope this additional data provides a clearer mechanistic understanding of the upstream signaling axis regulating proteasome activation in the context of mitochondrial dysfunction. Moreover. We hope the reviewer agree that accumulation of depolarized mitochondria itself can drive mitochondrial protein degradation that in turn promotes T cell exhaustion.

3) The inclusion of circadian regulators such as REV-ERBα/β and BMAL1—while conceptually interesting—feels peripheral to the central heme-proteasome axis. These elements are not tested or shown to be essential for the phenotypes described. The authors may consider either strengthening this arm experimentally. The authors mention that heme alters the transcriptional activity of these circadian factors based on transcriptomic trends and gene knockout phenotypes. However, this lacks direct biochemical validation that regulatory heme binds to and functionally modulates these transcription factors. The following experiments could help address these points: a) Direct binding assays demonstrating interaction between heme and circadian TFs (REV-ERBs, CLOCK, or BMAL1). b) Reporter assays or chromatin occupancy analyses showing that heme modulates the transcriptional output of circadian targets in T cells. c) Rescue experiments using heme-binding mutants of these TFs to establish causality.

Response: We sincerely appreciate the reviewer's positive feedback on our findings in circadian regulator and the insightful suggestions. We agree with the reviewer that the data regarding the circadian rhythm components, such as REV-ERBα/β and BMAL1, is not sufficiently strong to support the conclusions drawn. While the potential link between heme and circadian regulation is conceptually interesting, as pointed out, we have not experimentally validated the direct interaction between heme and these transcription factor. In line with the editor's suggestion the suggestion raised by reviewers, we decide to remove this aspect from the manuscript to maintain a clear and focused narrative on the central proteasome-heme axis. We believe this will strengthen the overall impact of our findings. We also hope the reviewer find our new results and experiments indeed strengthen this direction and our claims.

4) Although the paper introduces RH as a mediator of transcriptional reprogramming (via Blimp1 induction and Bach2 degradation), key questions remain unanswered: What quantity or localization of RH is required to alter T cell fate? How is RH imported into the nucleus and how stable is it under exhaustion conditions? Are alternative pathways of heme degradation/export (e.g., HO-1, FLVCR1) active in T cells or the TME?

Response: We sincerely appreciate the reviewer's critical comments. Please see our replies below regarding to these points.

a. Localization and quantity of RH required to alter T cell differentiation:
   In our study, we did confirm Pgrmc2, a chaperon has been reported to be responsible for import RH into nucleus in mammalian cells (Galmozzi et al., Nature, 2019), is critical for driving T cell exhaustion. As shown in Revised Figure 5 of the revised manuscript, genetic ablation of Pgrmc2 significantly improved the effector function of $CD8^+$ T cells, restoring mitochondrial function and reducing depolarization. Moreover, both Pgrmc2-deficient P14 cells and Her2 CAR T cells can elicit stronger anti-tumor responses compared to control groups. These findings demonstrated that nuclear import of RH is a key determinant in enforcing the transcriptional and functional program of T cell exhaustion. Direct quantification of nuclear RH, however, remains technically

challenging. The limited yield of nuclear lysates from T cells precludes reliable RH measurement, and sensitive reported capable of monitoring RH dynamics within the nucleus are currently unavailable. Nevertheless, we hope the reviewer agree that our complementary approaches, including hemin treatment, the use of a heme-resistant Bach2 mutant, and genetic ablation of Pgrmc2, collectively provide compelling evidence that heme signaling axis is a critical driver of driving T cell exhaustion in response to the accumulation of depolarized mitochondria.

b. Degradation of RH:

Regarding the degradation of RH, heme oxygenase (HO) serves as the principal catabolic enzyme, converting heme into biliverdin, carbon monoxide, and free iron through an NADPH- and oxygen-dependent reaction. However, the TME is typically hypoxic, which can limit the activity of HO. Based on this, we hypothesize that RH is more stable in exhausted T cells because the hypoxic conditions limit the activity of HO. Further, HO-1, an enzyme that releases iron, can contribute to $CD8^+$ T cell dysfunction and ferroptosis (Xu et al., Immunity, 2021; Ma et al., Cell Metab, 2021). Consistent with this, we indeed found that overexpressing HO-1 in $CD8^+$ T cells can reduce RH levels in normoxic condition, but failed to reduce RH levels under the hypoxic condition. Moreover, HO-1 overexpression failed to reduce exhaustion severity and restore cytokine production in TILs (**Reviewer Figure 3**). Thus, we speculate that the hypoxic TME may sustain elevated RH levels in TILs accumulating depolarized mitochondria by limiting HO-1-mediated RH catabolism, thereby impairing an essential pathway for RH clearance.

For reviewer Figure 3

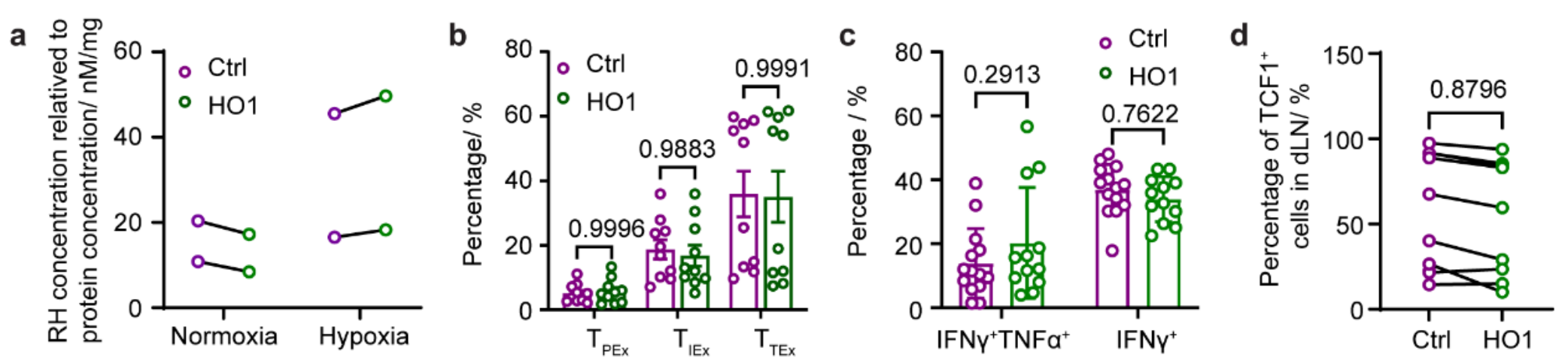

Reviewer Figure 3. a. Regulatory heme levels in $CD8^+$ T cells overexpressing HO1 and their vehicle control (Ctrl). b. Differentiation of P14 T cells with HO1 overexpression or control (Ctrl) in Yumm1.7-gp33 melanoma tumors. c. Cytokine production by P14 T cells with HO1 overexpression or control (Ctrl) in Yumm1.7-gp33 melanoma tumors. d. $TCF1^+$ P14 T cells in the draining lymph node (dLN) of Yumm1.7-gp33 melanoma-bearing mice.

c. Role of FLVCR1 and heme export in T cells within the TME:

FLVCR1 is a heme exporter that transports heme out of cells, following the heme gradient (Philip et al., J Immunol, 2015). However, the tumor interstitial fluid contains abnormally high amounts of heme (**Revised Extended Data Figure 3d**), which can be

caused by hemorrhage in tumors. This result suggests that $CD8^+$ TILs may be unable to effectively expel RH through FLVCR1 due to the less favorable heme gradient. Together with our observation that the elevated heme levels in the tumor interstitial fluid and the limited impact of HO1 overexpression, these findings suggest that heme degradation and export pathways may not be fully engaged in T cells residing within the TME. We have addressed this possibility in the Discussion and hope the reviewer find our interpretation well supported by our data.

5) The rationale for the last section of the paper -CD19 CARs- is relatively weak. Why would the CAR T cells become exhausted during manufacturing? Why would they exhibit proteasome dysregulation in this context? Isn't this induced by the TME or the general cancer macroenvironment?

Response: We sincerely appreciate the reviewer's critical comments and apologize for misunderstandings may be caused by the way we described our results. We appreciate the opportunity to clarify this point. As shown in Revised Figure 6g, we observed substantial variation in basal proteasome activity among individual CAR-T cell products prior to infusion. These results highlight that the intrinsic differences in proteasome activity at baseline, which are present before the cells ever encounter targets in patient, may critically influence subsequent therapeutic efficacy. That is why we decide to examine whether manipulating proteasome activity during production can empower human CAR-T cell products ability to resist to exhaustion in response to repeated antigen exposure.

We apologize for misleading the reviewer in our original manuscript. We did not monitor the expression of exhaustion markers directly in CD19 CAR-T cell products right after production. Instead, we treated the CAR-T cells with a proteasome inhibitor, Bortezomib, during manufacturing to reduce basal proteasome activity. The CD19 CAR-T cell products were then co-cultured with Nalm6 target cells to mimic repeated antigen encountering. We found that CD19 CAR-T cell products generated in the presence of Bortezomib exhibited markedly reduction of exhaustion markers, including PD1, TIM3, and CD39 (**Revised Figures 6h** and **Revised Extended Figure 6g**). Moreover, CAR-T cell products produced under Bortezomib exposure elicited superior anti-tumor responses in vivo, as reflected by enhanced tumor control and improved survival outcomes (**Revised Figure 6i-k**). Thus, our study proposes that proteasome activity prior to infusion could be a critical factor influencing the efficacy of CAR-T cells, and reducing this activity during the manufacturing process may help to mitigate exhaustion and enhance their anti-tumor response. We hope the reviewer find our interpretation well supported by our results and our reply address the concern.

6) The effects of bortezomib pre-treatment on CAR-T cell function are modest, with some improvement in tumor burden and survival. Importantly, minimal evidence is provided that the same exhaustion or proteasome dysregulation observed in TILs occurs during CAR-T

manufacturing. This is a critical assumption upon which the therapeutic implications rest. The authors should directly measure proteasome activity, mitochondrial fitness, and exhaustion markers in CAR-Ts during ex vivo expansion and progressively post-transfer. Also, it is important to note that Reference #61 compromises the therapeutic novelty of the current study.

Response: We appreciate the reviewer's thoughtful comments and suggestions. To evaluate whether proteasome inhibitor pre-treatment enhances CAR-T cell anti-tumor efficacy, we generated CAR-T cells in the presence of Bortezomib by using T cells from healthy donors. Although comparative data on proteasome activity between healthy donor-derived T cells and those from B-ALL patients are currently lacking, this remains an interesting direction for future study. The relatively modest improvements observed here may reflect the inherently lower proteasome activity of healthy donor T cells. However, this does not undermine our central finding: inhibiting proteasome activity can attenuate CAR-T cell exhaustion and enhance their anti-tumor activity in response to repeated antigen exposure, as demonstrated across our experimental systems.

Regarding the reviewer's suggestion to directly measure proteasome activity, mitochondrial fitness, and exhaustion markers during ex vivo expansion and post-transfer, we encountered technical challenges in analyzing $CD8^+$ CAR-T cells from peripheral blood. Despite our efforts, the cell count was too low for meaningful analysis. This may be due to $CD8^+$ CAR-T cells homing predominantly to the bone marrow following transfer in leukemic mice, and potentially due to the absence of $CD4^+$ CAR-T cells, which could support the persistence of $CD8^+$ CAR-T cells in peripheral blood. Although we were unable to directly measure exhaustion markers in $CD8^+$ CAR-T cells from peripheral blood, our in vitro co-culture experiments with target cells revealed that bortezomib pre-treated $CD8^+$ CD19 CAR-T cells expressed lower levels of exhaustion markers upon repeated antigen exposure (**Revised Figure 6h–k**), further supporting the functional improvement in these cells.

In addition, we performed integrated scRNA-seq and scATAC-seq to investigate the effects of bortezomib treatment on the epigenetic and transcriptional landscape of CAR-T cells. Chromatin accessibility analysis revealed that, in the bortezomib pre-treated group, several transcription factor (TF) binding motifs were downregulated, including KLF12, ETS1, RBPJ, and PRDM1, all of which are known to promote terminal exhaustion. These TFs exhibited decreased chromatin accessibility, suggesting that bortezomib pre-treatment promote memory associated epigenetic programs in CAR-T cells (**Revised Figure 6p**). Furthermore, CD19 CAR-T cells generated in the presence of bortezomib had higher frequency of the cell cluster with memory-like T cell features upon co-cultured with Nalm6 target cells. (**Revised Figure 6n**). In the exhaustion cluster, proliferation, cytotoxicity, and cytokine scores were significantly higher in the bortezomib pre-treated group compared to controls (**Revised Figure 6o**). Altogether, our results indicate that bortezomib pre-treatment endows CAR-T cells with memory-like characteristics while augmenting their functional competence, thereby contributing to superior anti-tumor responses observed in vivo.

In contrast to Reference #61, which investigated the effects of bortezomib in tumor-bearing mice and observed increased $CD8^+$ T cell infiltration and cytokine secretion, our study focuses on directly assessing the effects of proteasome inhibition on CAR-T cells themselves. The study in Reference #61 involved direct bortezomib treatment in mice, which could also lead to tumor cell apoptosis, potentially affecting tumor volume and T cell infiltration. This introduces confounding variables that do not allow for a clear distinction between bortezomib's direct effects on T cells versus indirect effects through tumor cell apoptosis. In contrast, our study directly demonstrates how proteasome inhibition in T cells enhances their anti-tumor efficacy, independent of the direct effects on tumor cells.

We hope these additional clarifications and new experimental results address the reviewer's concerns and further highlight the novelty of our findings.

7) Unclear how and why short-term pre-treatment with bortezomib could induce long-lasting effects in the transferred CAR-T cells. Could RH-edited CAR T cells be tested to define if they show improved therapeutic efficacy in vivo?

Response: We appreciate the reviewer' for raising this point. To investigate why short-term pre-treatment with bortezomib induces long-lasting effects in the transferred CAR-T cells, we performed integrated scRNA-seq and scATAC-seq (scMulti-omics) on CD19 CAR-T cells produced in the presence of bortezomib (pre_Bort) or control vehicle (pre_Ctrl). We also subjected them into a co-culture with Nalm6 target cells (co-cultured_Bort and co-cultured_Ctrl) (**Revised Figure 6l**). Our analysis revealed six distinct clusters of CAR-T cells (**Revised Figure 6m**). By using T cell-related marker genes (**Revised Extended Data Figure 6h**) and scores for memory, exhaustion, and cytotoxicity, we identified CAR-T_2 as the memory-like cluster and CAR-T_4 as the exhaustion cluster. Notably, the proportion of memory-like cells was significantly increased in the co-cultured_Bort group (**Revised Figure 6n**). Within the CAR-T_4 cluster (exhaustion cluster), proliferation, cytotoxicity, and cytokine scores were all significantly elevated in the co-cultured_Bort group compared to the co-cultured_Ctrl group (**Revised Figure 6o**). Moreover, analysis of transcription factor (TF) binding motifs showed that several TFs, including KLF12, ETS1, RBPJ, and PRDM1, were less enriched in control groups and all of these TFs are known to promote terminal exhaustion. In contrast, TFs associated with memory-like features, including KL2, EGR2, RUNX2, and FOXO1, were enriched in Bortezomib treated group (**Revised Figure 6p**). Further analysis of chromatin accessibility revealed that *PRDM1* and *PDCD1*, markers of exhaustion, showed decreased chromatin accessibility in the co-cultured Bortezomib group (**Revised Figure 6q**). Overall, our data indicate that the epigenetic modulation induced by bortezomib pre-treatment enhances the CAR-T cells' functional capacity, leading to long-lasting effects when they encounter repeated antigen exposure. Although the durability of these effects remains to be fully defined, the pronounced alterations in chromatin accessibility and TF-enriched motifs highlight that a short-term exposure of Bortezomib is sufficient to reprogram, CAR-T cells. Moreover, Bortezomib

has been shown to function as a slowly irreversible proteasome inhibitor with a long target-residence time, a property thought to underlie the persistence of proteasome inhibition even after plasma drug levels decline (Carlyn Rose C Tan, Clin Pharmacokinet., 2019). This pharmacodynamic profile likely contributes to the sustained molecular changes we observe.

In response to the second part of the reviewer's question regarding whether RH-edited CAR-T cells could improve therapeutic efficacy in vivo, we leveraged Pgrmc2-deficient T cells, which exhibit reduced nuclear transport of RH. We generated Her2 CAR-T cells from these Pgrmc2-deficient T cells and transferred them into Yumm1.7-Her2-bearing mice. Strikingly, Pgrmc2-deficient Her2 CAR-T cells exhibited markedly improved anti-tumor efficacy compared to the wild type Her2 CAR-T cells (**Revised Figure 5k–m** and **Extended Data Figure 5i**). We hope these additional clarifications and new experimental results address the reviewer's concerns and further highlight the novelty of our findings.

Additional Comments

• Please clarify whether immunoproteasome components are directly implicated.

Response: We thank the reviewer for raising this important point. Based on the point raised by the reviewer, we performed additional analysis of our proteomic results. Our updated proteomic analyses showed that T cells constitutively express immunoproteasome subunits at baseline. However, terminally exhausted $CD8^+$ T cells preferentially upregulate the catalytic subunits of the constitutive proteasome, including PSMB5, PSMB6, and PSMB7. These subunits are minimally expressed in naïve, effector, and memory T cells but become strongly induced during exhaustion, indicating that constitutive proteasome activity is specifically enhanced in terminally exhausted T cells (**Revised Extended data Figure 1e**). Moreover, all T cell subsets expressed similar levels of immunoproteasome specific subunits, including PSMB8, PSMB9, PSME1, and PSME2. Notably, our functional analyses reveal that the proteasome-driven protein degradation and the consequent RH accumulation depend on the constitutive proteolytic activity of the proteasome rather than immunoproteasome-restricted antigen-processing functions. Together, these findings demonstrate that exhausted T cells preferentially upregulate constitutive proteasome subunits, and the mitochondrial protein degradation–RH axis we identify is executed through an enhanced general proteolytic capacity of the proteasome complex. We hope these additional analyses and clarifications fully address the reviewer's concern.

• The manuscript has several typos and grammar errors that need attention.

Response: We thank the reviewer for pointing this out. We have thoroughly reviewed the manuscript and corrected all identified typos and grammar errors. We also conducted a careful revision of the manuscript to ensure that the language is clear, concise, and scientifically precise. We believe these changes improve the readability and clarity of the manuscript.

Referee #5 (Remarks to the Author):

The results presented do not demonstrate sufficient novelty to meet the publication standards for Nature. In particular, the in vivo data lack robustness (see reviewer Figure 7). Greater mechanistic insight may be needed to support the conclusions. The novelty here is not the connection of heme to Bach1 in T cells (Gupta Blood 2023). The central novel claim could be that mitochondrial depolarization leads to increased proteasome activity, which selectively degrades mitochondrial proteins to elevate heme levels. However, the mechanism behind this is unclear. Proteasome function is ATP-dependent, so it is surprising that membrane depolarization, typically associated with mitochondrial ATP depletion, would enhance proteasome activity. Furthermore, the basis for the selective degradation of mitochondrial proteins is not well explained. Moreover, the connection to clock genes is not clear. Heme-BACH1 makes sense but not sure how they got to BMAL1.

Response: We sincerely appreciate the reviewer's critical comments. Please see our replies below regarding to these points.

1. **In vivo data robustness (Reviewer Figure 7):**

Reviewer Figure 7 shows that Bach2^{MUT}-overexpressing P14 T cells failed to enhance cytokine secretion, and Bach2^{MUT} overexpression in anti-Her2 CAR-T cells did not improve survival. This suggests that Bach2^{MUT} preserves stem-like features at the expense of effector function. This is consistent with recent work by Philippa R. Barton et al. (Eur J Immunol. 2022), which shows that Bach2 overexpression blocks the acquisition of cytotoxic and effector functions in T cells. Moreover, this is also consistent with two studies will be published in Nature Immunology describing that sustained Bach2 expression is detrimental for mounting effector functions and anti-tumor responses in tumors (based on our communication with Tuoqi Wu and Yao Chen after our presentation in Keystone symposium). Thus, while T cell stemness is crucial, it is insufficient for optimal anti-tumor immunity. Effective $CD8^+$ T cells must balance stemness with differentiation into effector cells to achieve durable tumor control, as demonstrated by Zhou P. et al. (Nature, 2023), which has been discussed in our discussion section.

Beyond the overexpression of Bach2 mutant, we provide additional in vivo evidence demonstrating that modulating the proteasome-RH axis is sufficient to enhance CAR-T cell functions. Specifically, restricting nuclear import of heme via Prgmc2 ablation markedly enhanced the therapeutic efficacy of adoptive cellular immunotherapy (**Revised Figure 5k-m**). In parallel, pre-treating CAR-T cells with the proteosome inhibitor Bortezomib during production extended the survival of leukemia-bearing mice (**Revised Figure 6i-k**). Together, these results underscore the translational potential of targeting either heme trafficking or proteasome activity to boost CAR-T cell efficacy in vivo.

2. **Novelty of heme-Bach1 connection (Gupta Blood 2023):**

We believe the study referenced by the reviewer pertains to a meeting abstract from the 65th ASH Annual Meeting by Gupta et al. Their abstract reports that heme supplementation in CAR-

T cell manufacturing under non-stressful conditions (normoxia, nutrient sufficiency, and no repetitive TCR stimulation) without repeated antigen exposure decreased the proportions of LAG3⁺, TIM3⁺, CD62L⁺, and TCF1⁺ cells in both CD4⁺ and CD8⁺ subsets. However, their study focuses on steady-state features of CAR-T cells in a controlled environment, unlike our approach which aims to mimic the conditions encountered by tumor-specific T cells within the tumor microenvironment. In contrast, we primed CD8⁺ T cells, differentiated them into effector T cells, and then treated them with hemin plus anti-CD3 antibody for another 48 hours to mimic repeated antigen exposure (**Revised Extended Data Figure 3f**). Our results highlight that higher heme concentrations accelerate T cell exhaustion under conditions of repetitive TCR stimulation (**Revised Figure 3**). In fact, when we treated resting effector T cells with hemin for 48 hours *without repeated antigen exposure*, we observed partial similarities to Gupta et al.'s findings, but without the enhanced cytokine production or a decline in the TCF1⁺ population (**Reviewer Figure 4**). These experiments suggest that repetitive TCR stimulation is a key factor in determining how heme modulates T cell differentiation. We believe our experimental model better reflects the challenges that tumor-specific T cells face in vivo.

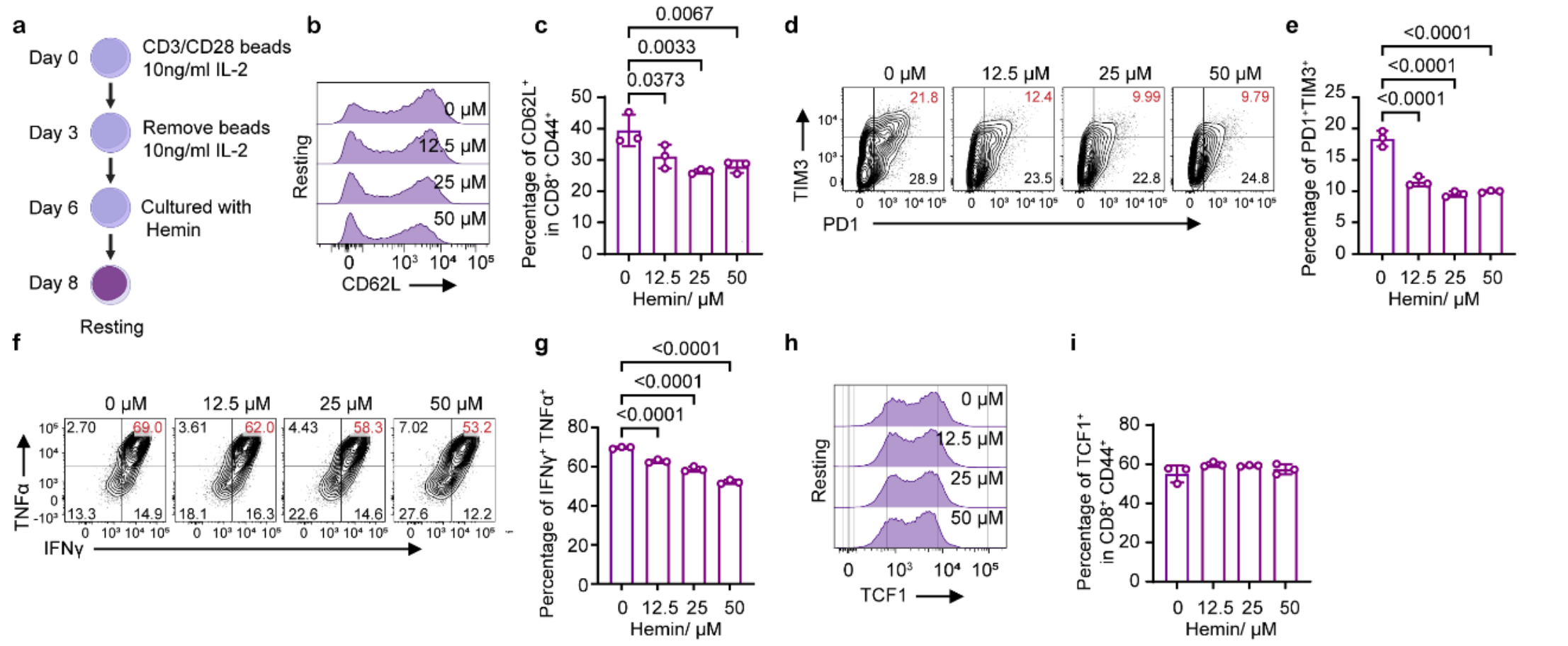

Reviewer Figure 4. The features of resting CD8⁺ T cells treated with Hemin.

a, Experimental workflow for in vitro treatment of resting CD8⁺ T cells with Hemin. **b,** Representative flow plots of CD62L expression of the resting CD8⁺ T cells. **c,** The quantification of CD62L expression in (b). n = 3. **d,** Representative flow plots of PD1 and TIM3 expression in the resting CD8⁺ T cells. **e,** The quantification of PD1⁺TIM3⁺ population in (d). n = 3. **f**, Representative flow plots of cytokine production in the resting CD8⁺ T cells. **g,** The quantification of IFNγ⁺TNFα⁺ population in (f). n = 3. **h,** Representative flow plots of TCF1 expression in the resting CD8⁺ T cells. **i,** The quantification of TCF1 expression in (h). n = 3. Data are representative of three independent experiments with similar results (c, e, g and i). Each symbol represents one individual. Data are means ± s.d. and were analyzed by one-way ANOWA (c, e, g and i).

3. Mechanism of mitochondrial depolarization and proteasome activity:

We appreciate that the reviewer raised an important question regarding how mitochondrial

depolarization leads to increased proteasome activity, especially considering that proteasome function is ATP-dependent. First, it has been reported that when intracellular ATP levels decline, cells can engage compensatory pathwas or recalibrate existing ones to preserve proteasome function. Moreover, physiological levels of ATP has been reported to suppress proteasome activity, whereas reduced ATP levels can enhance proteasome activity (Hongbiao Huang, Cell Res,. 2010). Thus, it is possible for T cells accumulating depolarized mitochondria to engage proteasome-mediated protein degradation. By analyzing proteomic results of TILs with different mitochondrial activity, our results revealed that MDR/MG^{low} subset significant increased expression of proteasome components (**Revised Figures 2a-b**), indicating that mitochondrial dysfunction is associated with proteasome activation. To further investigate this, we induced the accumulation of depolarized mitochondria in T cells by co-treating them with Oligomycin A, Antimycin A, and Mdivi-1. Under this condition, the MDR/MG^{low} subset displayed a higher proteasome activity toward mitochondrial component, as demonstrated by a proteasome reporter assay and corroborated by proteomic analyses (**Revised Figures 2c-f**).

To further strengthen the mechanistic understandings, we performed an additional set of experiments to identify ubiquitinated proteins in MDR/MG^{high} and MDR/MG^{low} T cells. We found that ubiquitinated substrates in MDR/MG^{low} T cells were predominantly enriched within the mitochondrial compartment (**Revised Figures 2j-k**). Moreover, we identified the E3 ligase CBL-B, upregulated in MDR/MG^{low} T cells (**Revised Figure 2l**), as a key mediator of mitochondrial protein degradation and the associated increase in RH release in MDR/MG^{low} T cells (**Revised Figures 2m-p**). Together, these findings indicate that mitochondrial depolarization initiates a CBL-B-dependent, proteasome-mediated, protein degradation that selectively degrades mitochondrial proteins.

4. Selective degradation of mitochondrial proteins:

In addition to points mentioned above, to address the selective degradation of mitochondrial proteins, we performed proteomics to analyze ubiquitinated proteins in human T_E, T_M, and T_{TEx} cells (**Revised Figure 1g**). We found that T_{TEx} cells had ubiquitinated proteins enriched in mitochondria, whereas T_M cells had ubiquitinated proteins enriched in the cytoplasm (**Revised Figure 1h**), indicating that T_{TEx} cells preferentially degrade mitochondrial protein. We further identified CBL-B as a key mediator of mitochondrial protein degradation in exhausted T cells (**Revised Figures 1j-k**). These findings suggest that a CBL-B-dependent, proteasome-mediated, protein degradation preferentially degrades mitochondrial proteins.

5. Proteasome activity despite mitochondrial ATP depletion:

We would like to clarify two aspects: 1) Proteasome activity under reduced ATP levels: While proteasome activity is ATP-dependent, studies (e.g., Huang et al., Cell Research, 2010) show that proteasome activity can still be maintained or even enhanced at low ATP levels (50–100 μM). This suggests that exhausted T cells may have sufficient ATP to support proteasomal activity, even when ATP is reduced. 2) Role of the 20S proteasome: Under conditions of oxidative stress, cells can increase the expression of the 20S proteasome, which does not require

the 19S regulatory particle or ATP for activity. This is especially relevant in exhausted T cells, which are known to experience oxidative stress. Our proteomic data and proteasome activity assays (**Revised Figures 1d, Extended Data Figure 1e**) also support this model, showing enhanced proteasome activity in terminally exhausted T cells, likely driven by the 20S proteasome.

6. Connection to circadian clock genes and BMAL1:

We appreciate the reviewer's comment regarding the connection between heme and circadian regulation. While heme-BACH1 interactions are well-established, the link to BMAL1 and other circadian regulators was not sufficiently validated in our study. Our analysis identified upregulation of circadian rhythm regulators such as BHLH40 and CLOCK (**Revised Figure 3j**), which influence mitochondrial dynamics and metabolic adaptation in response to heme availability. However, as the reviewer points out, we have not directly validated the interaction between heme and circadian transcription factors such as BMAL1 and REV-ERBα/β. In response to the editor's suggestion, we have removed this aspect from the manuscript to maintain a more focused narrative on how proteasome-heme axis orchestrate T cell exhaustion.

Response (Xu et al.)

We would like to thank the editor for your letter and are delighted by the decision to offer, in principle, publication of our manuscript entitled "Proteasome-guided heme signaling axis contributes to T cell exhaustion" in Nature. We are grateful to the editor and the referees for the careful evaluation of our work and for the constructive and encouraging comments. We have revised the manuscript to address all points raised by the reviewers, to incorporate the suggested editorial changes, and to ensure compliance with the Nature Guide to Authors, while retaining all peer-reviewed data.

Please find our "point-to-point" response (in blue) to the editor and reviewer's comments below. We also highlighted modifications in blue in the revised manuscript.

Issues:

1. Please add references to the abstract (if applicable).

Response: As requested, we have added the relevant references to the abstract.

2. The number of references should generally not exceed 60. Currently you have 70 references

Response: We have reduced the number of references in the main text to 60.

3. Please create a separate reference list for any methods references, making sure that the numbering continues from the main text references.

Resonse: We have created a separate reference list for the Methods section. All methods-only references have been moved to this list, with numbering continuing from the main text references.

4. Please remove the main figures from the article file and re-supply them individually in an acceptable format such as EPS, AI, PS, PDF, PPT, PSD or XLS (for graphs) with editable vector files.

Response: We have removed the main figures from the article file and resubmitted them individually in AI format as editable vector files.

5. Please re-supply the Extended data figures individually in EPS, JPEG or TIF format.

Response: We have resupplied the Extended Data Figures individually in TIF format.

6. Please ensure that the text size in all figures is at least 5 pt Arial.

Response: We have verified that all main figures use Arial font with text sizes of 5-7 pt, and all Extended Data Figures use Arial font with text sizes of 8-10 pt.

7. Please describe each figure panel in the figure legend.

Response: We have revised the figure legends to ensure that all panels are clearly explained.

8. Please note that the legends for the main figures should not exceed 300 words. If it is not possible to reduce the length accordingly, please ensure that the final legends are as close as possible to 300 words.

Response: We have made substantial efforts to shorten the main figure legends in accordance with the 300-word guideline. The legends for Figures 2, 3, and 4 have been reduced to fewer than 300 words. However, Figures 1 and 5 contain a large number of panels (20 and 18, respectively) that require detailed description for clarity, and their legends therefore remain slightly longer (404 and 317 words). We have ensured that these legends are as concise as possible while still providing the necessary information.

9. Please reduce subheadings to 40 characters (with spaces) or less.

Response: We have revised all subheadings to ensure that each is 40 characters or fewer, including spaces.

10. There are potential third party rights issues in the figures. Please check the sources of all illustrations and clarify whether permissions are needed to adapt or reproduce them. Please make sure to include the relevant details in third party rights table when you resubmit (more information below). If Biorender or a similar software has been used, please also ensure to provide relevant licenses. In particular please check Figures 1g, 2(a,j), 3k, 6(i,l) Extended data Figure 1(d,f), 2c, 3f, 6a.

Response: We have carefully reviewed all figures for potential third-party rights issues, including Figures 1g, 2a,j, 3k, 6i,l (revised Fig. 1g,l, 2k, 3a, 5i,5l) and Extended Data Figures 1d,f, 2c, 3f, and 6a (revised Extended Data Fig. 1d,f, 2e and 6a). All illustrations were either generated by the authors or created using BioRender. The relevant BioRender licenses have been obtained and are provided.

11. Please make sure to provide a third party rights table (more information below) when you resubmit. If Biorender or similar software has been used, please also ensure to provide relevant licenses.

Response: A completed third-party rights table has now been included with the resubmission, detailing the source of each figure and confirming that no additional permissions are required.

12. Please note a "Supplementary figure 2" is cited in the text (line 492), but there are no SI figures provided.

Response: We apologize for the error. The citation should refer to Extended Data Figure 3d[1] rather than Supplementary Figure 2, and this has now been corrected in the text (line 423).

13. There are 11 Extended Data tables, but it doesn't seem like all of them are cited - please check. Note, these Extended Data Tables should also be changed to "Supplementary Tables" - please update all the text accordingly.

Response: We have carefully checked all the tables and confirmed that they are correctly cited in the text. In addition, all instances of "Extended Data Tables" have been updated to "Supplementary Tables" throughout the manuscript.

14. Please include Source Data for all mouse experiments (see relevant section below).

Response: We have included the Source Data for all mouse experiments as requested.

15. Note, we can only have 5 main figures max. Please shift one of the main figures to the Extended Data and update the text/callouts accordingly.

Response: We have revised Figure 2 by redistributing panels between Figure 1 and Extended Data Figure 2, and updated the text and legends accordingly.

16. The text is currently too long (~5300 words) - please cut to 4500-5000 words max.

Response: We have substantially reduced the length of the manuscript. After revision, the main text is now 4,369 words, which is within the recommended word limit.

17. Please ensure that all deposited sequences are released in time for publication.

Response: All deposited sequences have now been released and are publicly available.

Referees' comments:

Referee #3 (Remarks to the Author):

I am satisfied that the authors have addressed my comments.

Response: We thank the referee for their positive assessment and are pleased that our revisions have addressed their comments.

Referee #4 (Remarks to the Author):

The authors have satisfactorily responded to most of my comments and concerns. The paper has improved and is now more cohesive and mechanistic. I would respectfully suggest changing the tittle to "contribute to" instead of "drive" and justifying more thoroughly the transition from the analysis of this pathway in TILs vs. CAR-T cell manufacturing in the main text. Overall, this is an interesting and exciting story.

Reponse: We thank the reviewer for their thoughtful and positive comments. In response to the suggestion, we have revised the title by replacing “drive” with “contribute to”. In addition, we have expanded and clarified the discussion in the main text to justify the transition between the analysis of proteasome-guided heme signaling in TILs and its relevance to CAR-T cell manufacturing. Please check Page Line.

Referee #5 (Remarks to the Author):

The authors have done a good job of responding.

Response: We thank the reviewer for their positive feedback and are pleased that our revisions have adequately addressed their comments.

1 An, N. *et al.* Construction of a new anti-CD19 chimeric antigen receptor and the anti-leukemia function study of the transduced T cells. *Oncotarget* **7**, 10638-10649 (2016). https://doi.org:10.18632/oncotarget.7079
