# Supplementary material for: Proteasome-guided haem signalling axis contributes to T cell exhaustion
Source: Nature. 2026 Mar 18;653(8114):548–57. doi: 10.1038/s41586-026-10250-y (PMC13658038; doi:10.1038/s41586-026-10250-y)
Supplement: Supplementary file 4 [file 41586_2026_10250_MOESM3_ESM.zip › 41586_2026_10250_MOESM3_ESM-updated/2024-06-12937C-s3/Supplementary_table_guide.docx]

**SI Guide**

**Proteasome-guided heme signaling axis drives T cell exhaustion**

Yingxi Xu ^1,2,3,4,*^, Yangguan Shangtao ^3,4^, Yu-Ming Chuang ^1,2^, Tzu-Hsuan Chang ^1,2^, Wenbing Liu ^3,4^, Jhan-Jie Peng ^1,2,5,6^, Josep Garnica ^1,2,7^, Leling Xie ^3,4^, Pei-Chun Hsueh ^1,2^, Mei-Chun Lin ^1,2,8^, Yi-Hao Wang ^1,2^, Karina Lobo Hajdu ^1,2^, Yibo Wu ^9^, Anna Kohl ^1,2^, Maryam Akrami ^10^, Chen Wang ^11,12,13^, Alfred Zippelius ^10,14^, Wei Qi ^15^, Min Wang ^3,4^, Bugi Ratno Budiarto ^16,17^, Shih-Yu Chen ^16^, Zhengtao Xiao ^18^, Panagiota Vardaka ^19,20^, Rahul Roychoudhuri ^19,20^, Zhiliang Bai ^21^, Rong Fan ^21^, Santiago Carmona ^1,2,7^, Christoph Scheiermann ^11,12^, Yi-Ru Yu ^1,2^, Jianxiang Wang ^3,4 *^, Ping-Chih Ho ^1,2,22 *^

* Correspondence
Authors Affiliations

^1^Department of Oncology, University of Lausanne, Lausanne, Switzerland.

^2^Ludwig Institute for Cancer Research, University of Lausanne, Epalinges, Switzerland.

^3^National Key Laboratory of Blood Science, National Clinical Research Center for Blood Diseases, Institute of Hematology and Blood Diseases Hospital, Chinese Academy of Medical Sciences and Peking Union Medical College, Tianjin, China.

^4^Tianjin Institutes of Health Science, Tianjin, China.

^5^Graduate Institute of Clinical Medical Sciences, Chang Gung University, Taoyuan, Taiwan

^6^Center for Molecular and Clinical Immunology, Chang Gung University, Taoyuan, Taiwan

^7^Swiss Institute of Bioinformatics, Lausanne, Switzerland

^8^Department of Otolaryngology, National Taiwan University Hospital, Taipei, Taiwan

^9^Chemical Biology Mass Spectrometry Platform (CHEMBIOMS), Faculty of Sciences, University of Geneva, Geneve, Switzerland.

^10^Laboratory of Cancer Immunology, Department of Biomedicine, University of Basel and University Hospital of Basel, Basel, Switzerland.

^11^Department of Pathology and Immunology, Faculty of Medicine, University of Geneva, Geneva, Switzerland

^12^Geneva Centre for Inflammation Research, Geneva, Switzerland

^13^National Key Laboratory of Immunity and Inflammation, Suzhou Institute of Systems Medicine, Chinese Academy of Medical Sciences & Peking Union Medical College, Suzhou, China

^14^Medical Oncology, University Hospital Basel, Basel, Switzerland

^15^Novogene Co, Ltd, China

^16^Institute of Biomedical Sciences, Academia Sinica, Taipei, Taiwan

^17^Taiwan International Graduate Program in Molecular Medicine, National Yang Ming Chiao Tung University and Academia Sinica, Taipei, Taiwan

^18^School of Basic Medical Sciences, Xi'an Jiaotong University, Xi'an, Shaanxi, China.

^19^Department of Pathology, University of Cambridge, Tennis Court Road, Cambridge CB2 1QP, UK.

^20^Laboratory of Lymphocyte Signaling and Development, Babraham Institute, Cambridge CB22 3AT, UK.

^21^Department of Biomedical Engineering, Yale University, New Haven, CT, USA.

^22^College of Medical Science and Technology, Taipei Medical University, Taipei, Taiwan

Correspondence to be addressed to Y. X. at: [xuyingxi@ihcams.ac.cn](mailto:xuyingxi@ihcams.ac.cn), J. W. at: [wangjx@ihcams.ac.cn](mailto:wangjx@ihcams.ac.cn) and P.-C. H.: [ping-chih.ho@unil.ch](mailto:ping-chih.ho@unil.ch).

Table of contents:

SI Table 1 | Homeostasis gene sets. Homeostasis relative functional pathways and their URL are listed.

SI Table 2 | Normalised protein group abundance in the indicated group of human CD8^+^ T cells. Naive T cell (T_N_), memory T cell (T_M_), effector T cell (T_E_), T_PEx_, and T_TEx_ human CD8^+^ T cells were collected and subjected to proteomic analysis. Normalized protein group abundances are presented.

SI Table 3 | Normalized protein group abundances in the indicated human CD8^+^ T cell groups. MDR/MG^high^ and MDR/MG^low^ tumor-infiltrating lymphocytes (TILs) were sorted from tumors and treated with or without cycloheximide. Cells were then collected and subjected to proteomic analysis. Normalized protein group abundances are presented.

SI Table 4 | Gene list of degraded ETC proteins in the MDR/MG^low^ population. Proteomic analysis comparing MDR/MG^low^ and MDR/MG^high^ tumor-infiltrating lymphocytes (TILs) revealed significant degradation of mitochondrial electron transport chain (ETC) components and assembly factors. This degradation is associated with disruption of ETC complex structures and potential release of prosthetic groups, such as heme.

SI Table 5 | The transcription factors that involved in regulating the differention of CD8^+^ T cells. Summary of experimentally validated transcription factors involved in the regulation of CD8^+^ T cell differentiation.

SI Table 6 | Gene list of hemoprotein. Summary of hemoproteins and their associated references.

SI Table 7 | Gene sets used for chromatin remodeling analysis. P14^+^ Bach2^WT^ tumor-infiltrating lymphocytes (TILs) and P14^+^ Bach2^MUT^ TILs were sorted and subjected to scRNA-seq. The gene sets used for analyzing the differences in chromatin remodeling between the two groups are listed.

SI Table 8 | Gene sets used in feature score. Summary of gene sets used for the Cytotoxicity, Exhaustion, Memory, Proliferation, Proteasome, Interleukins and Interferons scores and their associated genes.

SI Table 9 | The p value of feature scores between different clusters. Single-cell RNA-seq was performed on CAR T cells isolated from patient peripheral blood mononuclear cells (PBMCs). Four distinct molecular clusters were identified: “Memory-like”, “Cytot-MKI67”, “Cytot-GZMB” and “Cytot-GZMK”. To characterize the differentiation status of these clusters, Exhaustion_score, Memory_score, and Proteasome_score were calculated for each cluster, and p values for comparisons between clusters are presented.
